# Supplementary material for: Interprofessional collaboration between hospital-based palliative care teams and hospital ward staff: A realist review
Source: PLoS One. 2025 Dec 19;20(12):e0338132. doi: 10.1371/journal.pone.0338132 (PMC12716714; doi:10.1371/journal.pone.0338132)
Supplement: S1 File — (PDF) [file pone.0338132.s001.pdf]

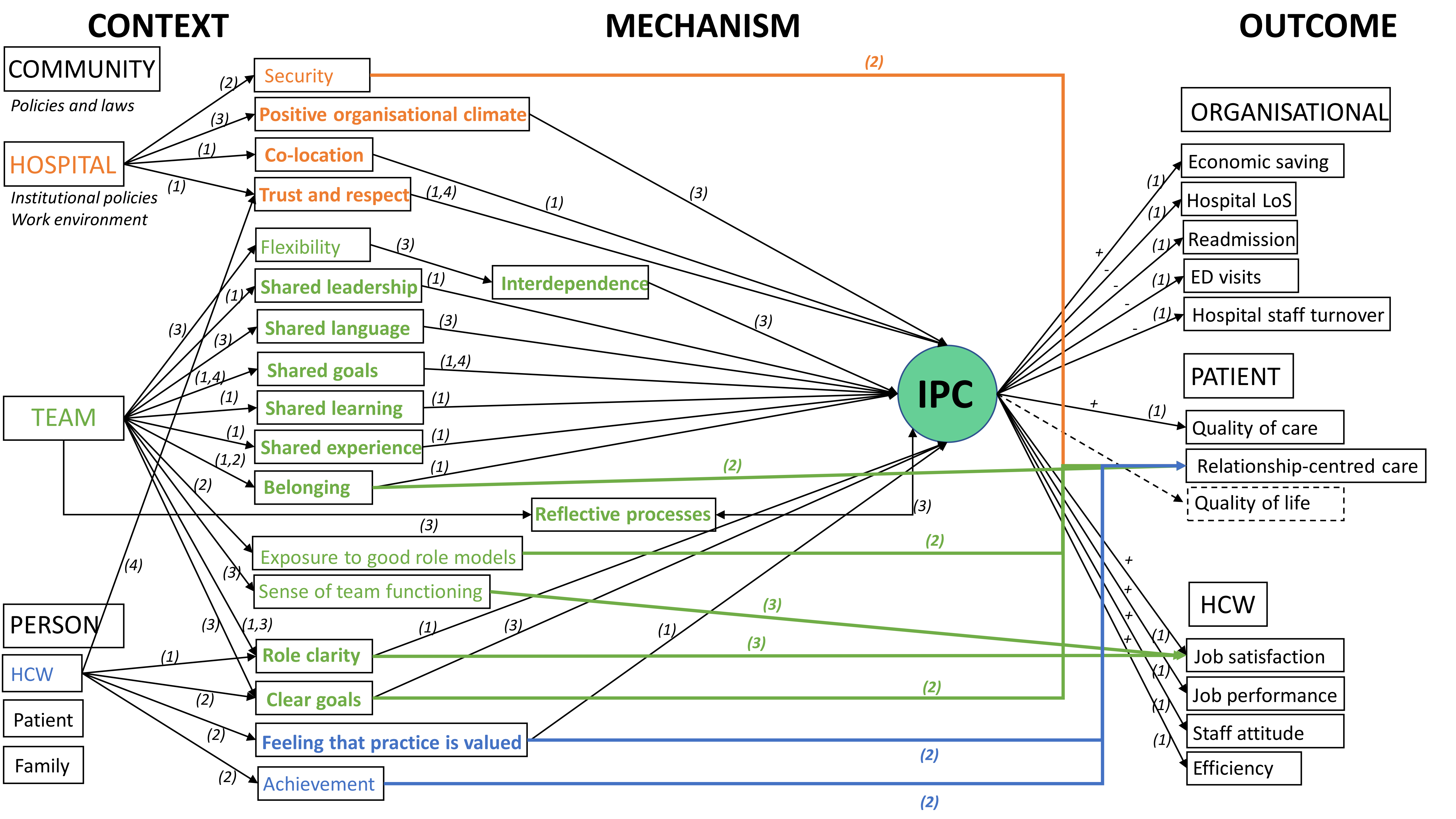
CONTEXT
MECHANISM
OUTCOME
COMMUNITY
Policies and laws
HOSPITAL
Institutional policies
Work environment
TEAM
PERSON
HCW
Patient
Family
Security
Positive organisational climate
Co-location
Trust and respect
Flexibility
Interdependence
Shared leadership
Shared language
Shared goals
Shared learning
Shared experience
Belonging
Reflective processes
Exposure to good role models
Sense of team functioning
Role clarity
Clear goals
Feeling that practice is valued
Achievement
IPC
ORGANISATIONAL
Economic saving
Hospital LoS
Readmission
ED visits
Hospital staff turnover
PATIENT
Quality of care
Relationship-centred care
Quality of life
HCW
Job satisfaction
Job performance
Staff attitude
Efficiency

References

(1) Holly Wei, Phyllis Horns, Samuel F. Sears, Kun Huang, Christopher M. Smith & Trent L. Wei (2022) A systematic meta-review of systematic reviews about interprofessional collaboration: facilitators, barriers, and outcomes. *Journal of Interprofessional Care*, 36:5, 735-749, DOI: 10.1080/13561820.2021.1973975

(2) Watson J. (2019). Developing the Senses Framework to support relationship-centred care for people with advanced dementia until the end of life in care homes. *Dementia (London, England)*, *18*(2), 545–566. https://doi.org/10.1177/1471301216682880

(3) Wittenberg-Lyles, E. M., & Oliver, D. P. (2007). The power of interdisciplinary collaboration in hospice. *Progress in Palliative Care*, *15*(1), 6–12. https://doi.org/10.1179/096992607X177764

(4) Barnard, A., Hollingum, C., & Hartfiel, B. (2006). Going on a journey: understanding palliative care nursing. *International journal of palliative nursing*, *12*(1), 6–12. https://doi.org/10.12968/ijpn.2006.12.1.20389
