# Supplementary material for: Interprofessional collaboration between hospital-based palliative care teams and hospital ward staff: A realist review
Source: PLoS One. 2025 Dec 19;20(12):e0338132. doi: 10.1371/journal.pone.0338132 (PMC12716714; doi:10.1371/journal.pone.0338132)
Supplement: S2 File — (DOCX) [file pone.0338132.s002.docx]

|  |
| --- |
| Supplementary file 2 |
| Search syntaxes |

| Moons et al., 2025 |
| --- |

**Search strategy for use in PubMed**

| ***"Hospitals"[MeSH Terms:noexp]*** *OR "hospital*"[Title/Abstract] OR "Hospital Medicine"[MeSH Terms] OR "Hospitalization"[MeSH Terms:noexp] OR "Hospital Departments"[MeSH Terms:noexp] OR "hospitals, general"[MeSH Terms] OR "oncology service, hospital"[MeSH Terms] OR "cardiology service, hospital"[MeSH Terms] OR "oncology ward*"[Title/Abstract] OR "oncological ward*"[Title/Abstract] OR "oncologic ward*"[Title/Abstract] OR "oncology department*"[Title/Abstract] OR "oncological department*"[Title/Abstract] OR "oncologic department*"[Title/Abstract] OR "oncology unit*"[Title/Abstract] OR "oncological unit*"[Title/Abstract] OR "oncologic unit*"[Title/Abstract] OR "geriatric ward*"[Title/Abstract] OR "geriatric department*"[Title/Abstract] OR "geriatric unit*"[Title/Abstract] OR "pneumology ward*"[Title/Abstract] OR "pneumology department*"[Title/Abstract] OR "pneumology unit*"[Title/Abstract] OR "pulmonology ward*"[Title/Abstract] OR "pulmonology department*"[Title/Abstract] OR "pulmonology unit*"[Title/Abstract] OR "cardiology ward*"[Title/Abstract] OR "cardiology department*"[Title/Abstract] OR "cardiology unit*"[Title/Abstract] OR "cardiological ward*"[Title/Abstract] OR "cardiological department*"[Title/Abstract] OR "cardiological unit*"[Title/Abstract] OR "cancer ward*"[Title/Abstract] OR "cancer department*"[Title/Abstract] OR "cancer unit*"[Title/Abstract]*  AND  ***"Palliative Care"[MeSH Terms]*** *OR "palliat*"[Title/Abstract] OR "Terminal Care"[MeSH Terms] OR "Terminal Care"[Title/Abstract] OR "end of life care*"[Title/Abstract] OR "eol care*"[Title/Abstract] OR "symptomatic treatment*"[Title/Abstract] OR "euthanasia*"[Title/Abstract] OR "resuscitation order*"[Title/Abstract] OR "resuscitation decision*"[Title/Abstract] OR "assisted suicide*"[Title/Abstract] OR "assisted death*"[Title/Abstract] OR "Advance Care Planning"[MeSH Terms:noexp] OR "Advance Care Planning"[Title/Abstract] OR "advance health care planning"[Title/Abstract] OR "advance medical planning"[Title/Abstract] OR "Hospice and Palliative Care Nursing"[MeSH Terms] OR "Hospices"[MeSH Terms] OR "hospice*"[Title/Abstract] OR "bereavement care"[Title/Abstract] OR "bereavement support"[Title/Abstract] OR "grief support"[Title/Abstract] OR "Palliative Medicine"[MeSH Terms] OR "Terminally Ill"[MeSH Terms] OR "terminally ill*"[Title/Abstract] OR "terminal ill*"[Title/Abstract] OR "terminal patient"[Title/Abstract:~2] OR "terminal patients"[Title/Abstract:~2] OR "terminally patient"[Title/Abstract:~2] OR "terminally patients"[Title/Abstract:~2] OR “dying patient*”[Title/Abstract] OR "Withholding Treatment"[MeSH Terms] OR "withholding treatment*"[Title/Abstract] OR "withdrawing treatment*"[Title/Abstract] OR "treatment withdrawal*"[Title/Abstract] OR "withholding care"[Title/Abstract] OR "withdrawing care"[Title/Abstract] OR "care withdrawal"[Title/Abstract] OR "treatment cessation"[Title/Abstract] OR “cessation of treatment”[Title/Abstract] OR "cessation of care"[Title/Abstract]*  AND  ***"Patient Care Team"[MeSH Terms****] OR "health care team*"[Title/Abstract] OR "healthcare team*"[Title/Abstract] OR "patient care team"[Title/Abstract:~2] OR "patient care teams"[Title/Abstract:~2] OR "medical care team"[Title/Abstract:~2] OR "medical care teams"[Title/Abstract:~2] OR "collaborative team"[Title/Abstract:~2] OR "collaborative teams"[Title/Abstract:~2] OR "interdisciplinary team"[Title/Abstract:~2] OR "interdisciplinary teams"[Title/Abstract:~2] OR "inter-disciplinary team"[Title/Abstract:~2] OR "inter-disciplinary teams"[Title/Abstract:~2] OR "multidisciplinary team"[Title/Abstract:~2] OR "multidisciplinary teams"[Title/Abstract:~2] OR "multi-disciplinary team"[Title/Abstract:~2] OR "multi-disciplinary teams"[Title/Abstract:~2] OR "transdisciplinary team"[Title/Abstract:~2] OR "transdisciplinary teams"[Title/Abstract:~2] OR "trans-disciplinary team"[Title/Abstract:~2] OR "trans-disciplinary teams"[Title/Abstract:~2] OR "decision making, shared"[MeSH Terms] OR "SDM"[Title/Abstract] OR "shared decision*"[Title/Abstract] OR "sharing decision*"[Title/Abstract] OR "shared learning"[Title/Abstract] OR "collaborative learning"[Title/Abstract] OR "Cooperative Behavior"[MeSH Terms] OR "cooperative behavior*"[Title/Abstract] OR "cooperative behaviour*"[Title/Abstract] OR "compliant behavior*"[Title/Abstract] OR "compliant behaviour*"[Title/Abstract] OR "teamwork"[Title/Abstract] OR "team work"[Title/Abstract] OR "collective decision*"[Title/Abstract] OR "Referral and Consultation"[MeSH Terms:noexp] OR "referral*"[Title/Abstract] OR "consultation*"[Title/Abstract] OR "Interprofessional Relations"[MeSH Terms] OR "interprofessional relation*"[Title/Abstract] OR "inter professional relation*"[Title/Abstract] OR "interdisciplinary communication"[Title/Abstract] OR "inter-disciplinary communication"[Title/Abstract] OR "multidisciplinary communication"[Title/Abstract] OR "multi-disciplinary communication"[Title/Abstract] OR "cross disciplinary communication"[Title/Abstract] OR "transdisciplinary communication"[Title/Abstract] OR "interdisciplinary collaboration*"[Title/Abstract] OR "inter-disciplinary collaboration*"[Title/Abstract] OR "multidisciplinary collaboration*"[Title/Abstract] OR "multi-disciplinary collaboration*"[Title/Abstract] OR "cross disciplinary collaboration*"[Title/Abstract] OR "transdisciplinary collaboration*"[Title/Abstract] OR "trans-disciplinary collaboration*"[Title/Abstract]* |
| --- |

**Search strategy for use in Embase**

| *'hospital'/de OR 'hospital*':ti,ab,kw OR ‘hospital medicine'/exp OR 'hospitalization'/exp OR 'hospital department'/exp OR 'general hospital'/exp OR 'ward'/de OR 'oncology ward'/exp OR 'geriatric ward'/exp OR ((('oncolog*' OR 'geriatr*' OR 'pneumolog*' OR 'pulmonolog*' OR 'cardiolog*' OR 'cancer') NEAR/2 ('ward*' OR 'department*' OR 'unit*')):ti,ab,kw)*  AND  *'palliative therapy'/exp OR 'palliat*':ti,ab,kw OR 'terminal care'/exp OR 'terminal care':ti,ab,kw OR 'end of life care*':ti,ab,kw OR 'eol care*':ti,ab,kw OR 'symptomatic treatment*':ti,ab,kw OR 'euthanasia*':ti,ab,kw OR 'resuscitation order*':ti,ab,kw OR 'resuscitation decision*':ti,ab,kw OR 'assisted suicide*':ti,ab,kw OR 'assisted death*':ti,ab,kw OR 'advance care planning':ti,ab,kw OR 'advance health care planning':ti,ab,kw OR 'advance medical planning':ti,ab,kw OR 'hospice nursing'/exp OR 'palliative nursing'/exp OR 'hospice'/exp OR 'hospice*':ti,ab,kw OR 'bereavement support'/de OR 'bereavement support':ti,ab,kw OR 'bereavement care':ti,ab,kw OR 'grief support':ti,ab,kw OR 'terminally ill patient'/exp OR 'terminally ill*':ti,ab,kw OR 'terminal ill*':ti,ab,kw OR (('terminal*' NEAR/3 'patient*'):ti,ab,kw) OR 'dying patient*':ti,ab,kw OR 'treatment withdrawal'/de OR ((('withhold*' OR 'withdraw*' OR 'cessation' OR 'ceas*') NEAR/2 ('treatment*' OR 'care')):ti,ab,kw)*  AND  *'collaborative care team'/exp OR ((('patient' OR 'medical' OR 'collaborative' OR 'interdisciplinary' OR 'inter-disciplinary' OR 'multidisciplinary' OR 'multi-disciplinary' OR 'transdisciplinary' OR 'trans-disciplinary') NEAR/3 'team*'):ti,ab,kw) OR 'health care team*':ti,ab,kw OR 'healthcare team*':ti,ab,kw OR 'shared decision making'/exp OR 'sdm':ti,ab,kw OR 'shared decision*':ti,ab,kw OR 'sharing decision*':ti,ab,kw OR 'shared learning':ti,ab,kw OR 'collaborative learning':ti,ab,kw OR 'teamwork'/exp OR 'teamwork':ti,ab,kw OR 'team work':ti,ab,kw OR 'collective decision*':ti,ab,kw OR 'cooperative behavio$r*':ti,ab,kw OR 'compliant behavio$r*':ti,ab,kw OR 'patient referral'/exp OR 'referral*':ti,ab,kw OR 'consultation*':ti,ab,kw OR 'interprofessional relation*':ti,ab,kw OR 'inter professional relation*':ti,ab,kw OR 'interdisciplinary communication'/exp OR ((('interdisciplinary' OR 'inter-disciplinary' OR 'multidisciplinary' OR 'multi-disciplinary' OR 'cross disciplinary' OR 'transdisciplinary' OR 'trans-disciplinary') NEAR/2 ('communication' OR 'collaboration*')):ti,ab,kw)* |
| --- |

**Search strategy for use in CINAHL**

| ***(MH "Hospitals")*** *OR* ***(MH "Hospital Medicine")*** *OR* ***(MH "Hospitalization")*** *OR* ***(MH "Hospital Units")*** *OR* ***(MH "Oncology Care Units")*** *OR* ***TI (“****Hospital*” OR ((“oncolog*” OR “geriatr*” OR “pneumolog*” OR “pulmonolog*” OR “cardiolog*” OR “cancer”) W2 (“ward*” OR “department*” OR “unit*”))****)*** *OR* ***AB (****“Hospital*” OR ((“oncolog*” OR “geriatr*” OR “pneumolog*” OR “pulmonolog*” OR “cardiolog*” OR “cancer”) W2 (“ward*” OR “department*” OR “unit*”))****)***  **AND**  ***(MH "Terminal Care+")*** *OR* ***(MH "Advance Care Planning")*** *OR* ***(MH “Hospice and palliative care nursing”)*** *OR* ***(MH "Hospices")*** *OR* ***(MH "Terminally Ill Patients+")*** *OR* ***TI (****“palliat*” OR “terminal care” OR “end of life care*” OR “eol care*” OR “symptomatic treatment*” OR “euthanasia*” OR “resuscitation order*” OR “resuscitation decision*” OR “assisted suicide*” OR “assisted death*” OR “advance care planning” OR “advance health care planning” OR “advance medical planning” OR “hospice*” OR “bereavement support” OR “bereavement care” OR “grief support” OR “terminally ill*” OR “terminal ill*” OR (“terminal*” W3 “patient*”) OR “dying patient*” OR ((“withhold*” OR “withdraw*” OR “cessation” OR “ceas*”) W2 (“treatment*” OR “care”))****)*** *OR* ***AB (“****palliat*” OR “terminal care” OR “end of life care*” OR “eol care*” OR “symptomatic treatment*” OR “euthanasia*” OR “resuscitation order*” OR “resuscitation decision*” OR “assisted suicide*” OR “assisted death*” OR “advance care planning” OR “advance health care planning” OR “advance medical planning” OR “hospice*” OR “bereavement support” OR “bereavement care” OR “grief support” OR “terminally ill*” OR “terminal ill*” OR (“terminal*” W3 “patient*”) OR “dying patient*” OR ((“withhold*” OR “withdraw*” OR “cessation” OR “ceas*”) W2 (“treatment*” OR “care”))****)***  **AND**  ***(MH "Multidisciplinary Care Team")*** *OR* ***(MH "Decision Making, Shared")*** *OR* ***(MH "Cooperative*** ***Behavior")*** *OR* ***(MH "Referral and Consultation")*** *OR* ***(MH "Interprofessional Relations+")*** *OR* ***TI (****((“patient” OR “medical” OR “collaborative” OR “interdisciplinary” OR “inter-disciplinary” OR “multidisciplinary” OR “multi-disciplinary” OR “transdisciplinary” OR “trans-disciplinary”) W3 (“team*”)) OR “health care team*” OR “healthcare team*” OR “SDM” OR “shared decision*” OR “sharing decision*” OR “shared learning” OR “collaborative learning” OR “Cooperative Behavio#r*” OR “Compliant Behavio#r*” OR “teamwork” OR “team work” OR “collective decision*” OR “referral*” OR “consultation*” OR “interprofessional relation*” OR “inter professional relation” OR ((“interdisciplinary” OR “inter-disciplinary” OR “multidisciplinary” OR “multi-disciplinary” OR “cross disciplinary” OR “transdisciplinary” OR “trans-disciplinary”) W2 (“communication” OR “collaboration*”))****)*** *OR* ***AB (****((“patient” OR “medical” OR “collaborative” OR “interdisciplinary” OR “inter-disciplinary” OR “multidisciplinary” OR “multi-disciplinary” OR “transdisciplinary” OR “trans-disciplinary”) W3 (“team*”)) OR “health care team*” OR “healthcare team*” OR “SDM” OR “shared decision*” OR “sharing decision*” OR “shared learning” OR “collaborative learning” OR “Cooperative Behavior*” OR “Compliant Behavior*” OR “teamwork” OR “team work” OR “collective decision*” OR “referral*” OR “consultation*” OR “interprofessional relation*” OR “inter professional relation” OR ((“interdisciplinary” OR “inter-disciplinary” OR “multidisciplinary” OR “multi-disciplinary” OR “cross disciplinary” OR “transdisciplinary” OR “trans-disciplinary”) W2 (“communication” OR “collaboration*”))****)*** |
| --- |

**Search strategy for use in WoS Core Collection**

| ***TS=(****“hospital*” OR ((“oncolog*”OR “geriatr*” OR “pneumolog*” OR “pulmonolog*” OR “cardiolog*” OR “cancer”) NEAR/2 (“ward*” OR “department*” OR “unit*”))****)***  **AND**  ***TS=(****“palliat*” OR “terminal care” OR “end of life care*” OR “eol care*” OR “symptomatic treatment*” OR “euthanasia*” OR “resuscitation order*” OR “resuscitation decision*” OR “assisted suicide*” OR “assisted death*” OR “advance care planning” OR “advance health care planning” OR “advance medical planning” OR “hospice*” OR “bereavement support” OR “bereavement care” OR “grief support” OR “terminally ill*” OR “terminal ill*” OR ((“terminal*”) NEAR/3 (“patient*”)) OR “dying patient*” OR ((“withhold*” OR “withdraw*” OR “cessation” OR “ceas*”) NEAR/2 (“treatment*” OR “care”))****)***  **AND**  ***TS=(****((“patient” OR “medical” OR “collaborative” OR “interdisciplinary” OR “inter-disciplinary” OR “multidisciplinary” OR “multi-disciplinary” OR “transdisciplinary” OR “trans-disciplinary”) NEAR/3 (“team*”)) OR “health care team*” OR “healthcare team*” OR “sdm” OR “shared decision*” OR “sharing decision*” OR “shared learning” OR “collaborative learning” OR “teamwork” OR “team work” OR “collective decision*” OR “cooperative behavior*” OR “compliant behavior*” OR “referral*” OR “consultation*” OR “interprofessional relation*” OR “inter professional relation*” OR ((“interdisciplinary” OR “inter-disciplinary” OR “multidisciplinary” OR “multi-disciplinary” OR “cross disciplinary” OR “transdisciplinary” OR “trans-disciplinary”) NEAR/2 (“communication” OR “collaboration*”))****)*** |
| --- |

**Search strategy for use in Scopus**

| ***TITLE-ABS(****“hospital*” OR ((“oncolog*”OR “geriatr*” OR “pneumolog*” OR “pulmonolog*” OR “cardiolog*” OR “cancer”) W/2 (“ward*” OR “department*” OR “unit*”)))* ***OR AUTHKEY(****“hospital*” OR ((“oncolog*”OR “geriatr*” OR “pneumolog*” OR “pulmonolog*” OR “cardiolog*” OR “cancer”) W/2 (“ward*” OR “department*” OR “unit*”))****)***  AND  ***TITLE-ABS(****“palliat*” OR “terminal care” OR “end of life care*” OR “eol care*” OR “symptomatic treatment*” OR “euthanasia*” OR “resuscitation order*” OR “resuscitation decision*” OR “assisted suicide*” OR “assisted death*” OR “advance care planning” OR “advance health care planning” OR “advance medical planning” OR “hospice*” OR “bereavement support” OR “bereavement care” OR “grief support” OR “terminally ill*” OR “terminal ill*” OR ((“terminal*”) W/3 (“patient*”)) OR “dying patient*” OR ((“withhold*” OR “withdraw*” OR “cessation” OR “ceas*”) W/2 (“treatment*” OR “care”))****)*** ***OR AUTHKEY(****“palliat*” OR “terminal care” OR “end of life care*” OR “eol care*” OR “symptomatic treatment*” OR “euthanasia*” OR “resuscitation order*” OR “resuscitation decision*” OR “assisted suicide*” OR “assisted death*” OR “advance care planning” OR “advance health care planning” OR “advance medical planning” OR “hospice*” OR “bereavement support” OR “bereavement care” OR “grief support” OR “terminally ill*” OR “terminal ill*” OR ((“terminal*”) W/3 (“patient*”)) OR “dying patient*” OR ((“withhold*” OR “withdraw*” OR “cessation” OR “ceas*”) W/2 (“treatment*” OR “care”))****)***  AND  ***TITLE-ABS(****((“patient” OR “medical” OR “collaborative” OR “interdisciplinary” OR “inter-disciplinary” OR “multidisciplinary” OR “multi-disciplinary” OR “transdisciplinary” OR “trans-disciplinary”) W/3 (“team*”)) OR “health care team*” OR “healthcare team*” OR “sdm” OR “shared decision*” OR “sharing decision*” OR “shared learning” OR “collaborative learning” OR “teamwork” OR “team work” OR “collective decision*” OR “cooperative behavior*” OR “cooperative behaviour*” OR “compliant behavior*” OR “compliant behaviour*” OR “referral*” OR “consultation*” OR “interprofessional relation*” OR “inter professional relation*” OR ((“interdisciplinary” OR “inter-disciplinary” OR “multidisciplinary” OR “multi-disciplinary” OR “cross disciplinary” OR “transdisciplinary” OR “trans-disciplinary”) W/2 (“communication” OR “collaboration*”))****)*** ***OR AUTHKEY(****((“patient” OR “medical” OR “collaborative” OR “interdisciplinary” OR “inter-disciplinary” OR “multidisciplinary” OR “multi-disciplinary” OR “transdisciplinary” OR “trans-disciplinary”) W/3 (“team*”)) OR “health care team*” OR “healthcare team*” OR “sdm” OR “shared decision*” OR “sharing decision*” OR “shared learning” OR “collaborative learning” OR “teamwork” OR “team work” OR “collective decision*” OR “cooperative behavior*” OR “cooperative behaviour*” OR “compliant behavior*” OR “compliant behaviour*” OR “referral*” OR “consultation*” OR “interprofessional relation*” OR “inter professional relation*” OR ((“interdisciplinary” OR “inter-disciplinary” OR “multidisciplinary” OR “multi-disciplinary” OR “cross disciplinary” OR “transdisciplinary” OR “trans-disciplinary”) W/2 (“communication” OR “collaboration*”))****)*** |
| --- |
