# Supplementary material for: Interprofessional collaboration between hospital-based palliative care teams and hospital ward staff: A realist review
Source: PLoS One. 2025 Dec 19;20(12):e0338132. doi: 10.1371/journal.pone.0338132 (PMC12716714; doi:10.1371/journal.pone.0338132)
Supplement: S3 File — (DOCX) [file pone.0338132.s003.docx]

|  |  |
| --- | --- |
| Supplementary file 3 | |
| Data-extraction forms of included studies |  |

| Moons et al., 2025 |
| --- |

| Author, country | Aim | Research design | Participants | Setting | Intervention | Outcomes | Limitations | Conclusion | Relevance |
| --- | --- | --- | --- | --- | --- | --- | --- | --- | --- |
| Alsirafy,  2015,  Saudi-Arabia  [1] | To describe the LoS of terminal hospital-ization  among in-hospital cancer deaths managed by a hospital-based  PC program comprising a PCC service and an inpatient PCU. | Retrospective  observational study  Apr 2009 - Dec 2011 | **N = 328**  Inpatients with cancer  **Inclusion**:  - Cancer diagnosis  - Died in-hospital  - Managed by PC service during terminal hospitalization | **King Fahad Specialist Hospital-Dammam**  **Discipline:** Oncology  **Hospital size:**  Not reported  **Hospital type:** Tertiary referral hospital where the only PC program in the Province is located. | **Co-management** of patients by the PCT in collaboration with the primary caring service.  PCT provides advice  regarding symptom control. | **LoS:**  - Shorter in PCU group (p<0.001)  **PCU group:**  **1. Univariate analysis:**  age, primary cancer, referring specialty and direct admission to PCU correlated significantly with LoS  **2. Multi-variate analysis:**  Age > 35 years and direct admission to PCU was independently associated with shorter LoS | - Only focus on terminal patients.  - Single-center retrospective study  - Use of hospital specific criteria for transfer to PC, which might differ from other institutions.  - No European setting, so how useful/ generalizable are findings | In conclusion, at least in our setting, the terminal hospitalization LoS of patients with cancer admitted by PC service  directly to PCU is shorter than that of those admitted by other  specialties and comanaged by a PCT. | Low |
|  |  |  |  |  | **Data collection** |  |  |  | **Rigour** |
|  |  |  |  |  | **Intervention group (PCC)** **(n=257):** patients admitted by services other than the PC service and referred to the PCT  **Control group (PCU)** **(n=71):** patients admitted by the PCT directly to the PCU.  Data of patients were obtained from the PC service database and review of the EHRs.  LoS = period from date of terminal hospitalization until the date of in-hospital death. |  |  |  | Low |

| Author, country | Aim | Research design | Participants | Setting | Intervention | Outcomes | Limitations | Conclusion | Relevance |
| --- | --- | --- | --- | --- | --- | --- | --- | --- | --- |
| Amano,  2014,  Japan.  [2] | To explore the effectiveness of a PCT by investigating potential differences in opioid  Prescription and LoS between patients who had had PCT involvement before admission to an inpatient hospice and those who had not. | Retrospectivecohort study  Dec 2012 – Nov 2013 | **N = 221**  Patients with cancer, who were transferred to the inpatient hospice  **Inclusion**:  - Adult  - No cognitive impairment  - Died in hospice during study  **Exclusion:**  - Died from unexpected complications | **Discipline:**  Oncology  **Hospital size:**  Large  (>1.000 beds)  **Hospital type:**  General hospital | PCT acts as an **advisory role** for other health care professionals to patients with palliative cancer.  Patients were followed every weekday and the PC physician prescribed or gave recommendations of medication. | **1) Daily dose of opioid converted to oral morphine before admission to hospice:**  significantly higher (p<0.001)  **2) Difference between the maximum opioid dose and the initial dose:**  not significantly different  **3) Rate of increase in opioids until death:**  not significantly different  **4) LoS in hospice:**  not significantly different  **5) LoS in hospice in a subgroup of patients transferred from a general hospital ward:**  Tends to be longer in the intervention group in the subgroup, however there was no significant difference (p=0.16). | - Retrospective study  - lack of  inter-rater reliability  - real effectiveness of PCT was not investigated, as it should not be assumed that any differential in opioid prescription is due only to the efficiency of a PCT. | A PCT contributes to not only higher but also more appropriate  use of opioids, which allows more rapid adjustment. | Low |
|  |  |  |  |  | **Data collection** |  |  |  | **Rigour** |
|  |  |  |  |  | **Intervention group (n=140):** patients receiving support from the PCT.  **Control group (n=81):** patients not receiving support from the PCT.  Opioids included in this study were those that were administered at regular intervals (morphine, oxycodone, and fentanyl). The principal investigator investigated them by chart review. To confirm the reliability and validity, another attending physician verified all evaluations. These items were  routinely recorded by physicians and nurses in our hospice. |  |  |  | Low |

| Author, country | Aim | Research design | Participants | Setting | Intervention | Outcomes | Limitations | Conclusion | Relevance |
| --- | --- | --- | --- | --- | --- | --- | --- | --- | --- |
| Anandan et al.,  2022,  USA  [3] | To determine if a partnership between hospital-medicine and  specialized palliative care would increase identification of patients with an advanced illness. | Retrospective observational, chart review study with 2 time periods.  **Time period 1:** 1 May , 2018 - 28 Feb 2019  **Time period 2**: 1 May 2019 – 29 Feb 2020 | **N = 3395**  Patients with advanced illness hospitalized on a medicine unit.  **In/exclusion:**  Not reported | **Discipline:**  Geriatrics  **Hospital size:**  Large  (802 staffed beds)  **Hospital type:**  quaternary-level teaching center  **Medicine unit** of 35 beds with:  - Care Model A = right side  - Care Model B = left side | **GaP consult service**  - Use of **identification triggers** to analyze need for GaP consultation  - Biweekly **rounding**  - **Triggers** for PC needs to consider during rounds | **Outcomes:**  **1) PCC**  **2) Time from admission to consult (days)**  **3) Time from consult to discharge (days)**  **4) Discharge disposition**  **5) Median LoS**  **6) Pain scores >6**  **7) 30-day readmission**  **8) DNR status**  🡪 Except from PC consult, no significant difference in any group comparison.  **Group comparisons:**  **1) Care Model A vs. Care Model B for time-period 2:**  - More PC consults in Care Model A (p=0.0013)  **2) Time-period 1 vs. time-period 2 for Care Model A:**  - More PCCs in time period 2 (p=0.0005)  - More discharge to subacute rehab in time period 2 (p=0.0312)  **3. Time-period 1 vs. time-period 2 for Care Model B**  - More discharge to subacute rehab in time period 2 (p=0.0027)  **4) Comparing timeframes**  GaP-triggered collaboration with “Care Model A,” was associated  with a more complex population (p=0.0056).  “Care Model B,” saw a trend down in the complexity of their patients (p=0.0215). | - The study did not capture the number of cases where PC needs were identified and deemed to be managed by the primary team.  - Certain patients were not consulted in a timely manner, due to the biweekly rounding structure.  - Setting: single institution with a culture receptive for PC before the intervention. The same intervention in a different culture may yield different results  - COVID19 cutted the intervention phase short, which may have influenced the results. | This study tested a  palliative care intervention on a resident-run staff hospitalist  team which led to increase identification of patients with  palliative care needs complex enough to increase GaP consultation. However, the study did not show a significant  decrease in 30 days readmissions, length of stay, orders for  DNR, or >6 pain scores. | Medium |
|  |  |  |  |  | **Data collection** |  |  |  | **Rigour** |
|  |  |  |  |  | **Data was collected from the enterprise EHR.**  **Time period 1 – preintervention phase (n=1707)**  1) Care Model A (n=946)  2) Care Model B (n=761)  **Time period 2 – intervention phase (n=1688)**  1) Care Model A – intervention group (n=857): GaP consult service.  2) Care Model B – control group (n=831): Call consults as per their prerogative. |  |  |  | Low |

| Author, country | Aim | Research design | Participants | Setting | Intervention | Outcomes | Limitations | Conclusion | Relevance |
| --- | --- | --- | --- | --- | --- | --- | --- | --- | --- |
| Artioli et al.,  2019,  Italy  [4] | To evaluate a new training program and its impact on trainees within a  hospital setting. | Mixed-method evaluation with concurrent triangulation | **N = 80**  Health professionals who represented all professional categories:  - physicians  - nurses  - head nurses  - nurse assistants  - technicians  - biologists  **In/exclusion:**  Not reported | **Discipline:**  Radiotherapy, Geriatrics and Nephrology/  Dialysis  **Hospital size:**  Large  (900 beds)  **Hospital type:**  General, public hospital | **Training program for PC:**  - Taught by PC specialists  - Duration of 4hours  - Focus on vision of PC, purpose of PCU, and sharing PC needs in hospital wards. | **Qualitative data:**  **Theme 1: Relationships between I- and II-levels, passing from obstacles to synergies:**  - Hospital professionals became aware of the broader picture and organization of PC  - Having a better comprehension of the roles and profiles of the PC specialists allowed the hospital professionals to be more attentive and sensitive in facilitating PCC  - Having clarified that PC needs can actually be met by the PC specialist once a PC plan is activated led the participants to feel a sense of synergy with colleagues that  seemed to facilitate inter-professional relations.  **Theme 3: Clinicians’ competences in EOL care: ‘becoming competent clinicians of the EOL’:**  **-** Physicians, after the training, were more likely to involve PC doctors, when before they felt more embarrassed.  **Theme 4: Integration between I- and II-levels: ‘a possible integrative model with the PC Unit’:**  **-** Participants’ understanding of the organization within the PCU seemed to have facilitated the involvement of PCC within the care.  - Participants became aware of the practical possibility of activating PC and showed the need for greater integration of PC specialists within the examined operating units. | - The number of questionnaires collected was not high, although the statistical analysis allowed us to identify significant differences.  - We measured only the impact on professional competencies and not effectiveness on patients and health outcomes. | The results obtained highlighted the significant amount  of knowledge acquired by the participants after training,  The qualitative analysis showed that meaning shifts emerged both as an interesting result of the training and as an innovative proposal for training evaluation. | Medium |
|  |  |  |  |  | **Data collection** |  |  |  | **Rigour** |
|  |  |  |  |  | Before-after evaluation  **Quantitative (n=77):**  Open-ended questionnaire about the  comprehension of the WHO definition of PC.  **Qualitative:**  Focus groups pre training (n=33)  Focus groups post training (n=29)  for qualitative feedback on PC knowledge and learning  Data triangulation to compare quantitative and qualitative results. |  |  |  | High |

| Author, country | Aim | Research design | Participants | Setting | Intervention | Outcomes | Limitations | Conclusion | Relevance |
| --- | --- | --- | --- | --- | --- | --- | --- | --- | --- |
| Atayee et al.,  2018,  USA  [5] | 1) To describe an inpatient PC pharmacist’s intervention and outcomes  2) To evaluate the impact on LoS, length from admission to PCC, and time from consult to discharge and death. | Retro-spective study between September 1, 2015, and March 30, 2017 | **N = 341**  Hospitalized patients seen by a part-time PC specialist pharmacist  **In/exclusion:**  Not reported | **Discipline:**  Not reported  **Hospital size:**  Not reported  **Hospital type:**  University of California, San Diego academic health system | **PC specialist pharmacist** as part of the PCT:  - Clinical responsibility 2 days/week  - Guides transdisciplinary PCT on pharmacology topics  - Recommendations but as per primary team request or permission  - Liaison between PCT an department of pharmacy  - Transmural function | **Most common PC pharmacist interventions:**  - Optimizing palliative medication regime  - Providing education to patient/providers  **Reasons for PC pharmacist consult:**  - 80.9% pain management  **Outcomes:**  - No significant difference in overall LoS between groups  - Time from consult to discharge or death and date of PCC were significantly longer for group 1 (p<0.005 for both outcomes)  **Subanalysis (n=110):**  - Pharmacist visit to discharge time was shorter than that for the overall PCT time  - If first PC pharmacist visit happened before dat 3, total hospital LoS, date of PCC, and time from consult to discharge or death were significantly reduced compared to patients seen on or after day 3 (p<0.005, p<0.05, p<0.005 respectively) | Retrospective, single center study  Limited focus on collaboration aspects, so rather lowly relevant  Part-time coverage PC pharmacist  Differences in patients seen by PC pharmacist may have led to observed results.  Data may be affected by reporter bias | This study provides the types of PC pharmacist  interventions as well as the documented outcomes a  PC pharmacist can provide on an inpatient palliative care  consult service as part of trans-disciplinary team | Low |
|  |  |  |  |  | **Data collection** |  |  |  | **Rigour** |
|  |  |  |  |  | Data collected from a **EHR flow sheet**, which is connected to the PCT’s participation in the PC Quality Network.  Pharmacist’s interventions and outcomes were self-reported  **2 Cohorts:**  Group 1: Patients with pharmacist consult (n=?)  Group 2: Patients with PCC but without pharmacist (n=?) |  |  |  | Low |

| Author, country | Aim | Research design | Participants | Setting | Intervention | Outcomes | Limitations | Conclusion | Relevance |
| --- | --- | --- | --- | --- | --- | --- | --- | --- | --- |
| Autor et al.,  2013  USA  [6] | to better understand whether nurses in an acute care hospital accurately understand PC. | Cross-sectional, survey study | **N = 143**  Nurses in  - oncology (n=38)  - intensive care (n=62)  - heart failure (n=43)  **In/exclusion:**  Not reported | **Discipline:**  Oncology, intensive care, and heart failure.  **Hospital size:**  Large  (800 beds)  **Hospital type:**  Tertiary care referral hospital | None | They link knowledge of PC (amount of correct answers) to some contextual factors such as:  - experience as oncology nurse  - experience as hospice nurse  - knowing the institution has a PCT  - total years of nursing experience  - … | Cross-sectional study in 1 setting  No in- or exclusion criteria reported  - self-developed questionnaire, however good reporting on testing of this questionnaire | Our study  suggests that as many as 7 of 10  nurses do not understand the fundamental tenet that PC is compatible with curative, treatment. This finding alone suggests that nurses are likely to  inadvertently overlook patients who might otherwise benefit from PC. | Low |
|  |  |  |  |  | **Data collection** |  |  |  | **Rigour** |
|  |  |  |  |  | Online questionnaire: the Palliative Care Quiz for Nurses for assessing knowledge and identify misconceptions about PC.  8 demographic questions |  |  |  | Low |

| Author, country | Aim | Research design | Participants | Setting | Intervention | Outcomes | Limitations | Conclusion | Relevance |
| --- | --- | --- | --- | --- | --- | --- | --- | --- | --- |
| Barrat et al.,  2018,  UK  [7] | To assess the effectiveness of a novel MDT meeting on the assessment of a patient’s PC needs. | Retrospective chart review study  Jan - July 2016 | **N = 72**  Patients with ILD  **In/exclusion:**  Not reported | **Discipline:**  Pulmonology (ILD)  **Hospital size:**  Not reported  **Hospital type:**  Not reported | **Collaborative MDT meeting** between PC, psychology, and ILD teams:  - Once every 6 weeks  - To assess and provide support to patients and their caregivers. | Statistically significant increases in documentation of cardiopulmonary resuscitation discussions (pre-MDT 38.5% vs post-MDT 78.3%)  Increased referrals to the hospice-delivered fatigue and breathlessness course (pre-MDT 30.8% vs post-MDT 67.4%)  Increased referrals to the specialist palliative care services (pre-MDT 38.5% vs post-MDT 73.9%) | - Small sample  - Retrospective study  - Setting is not discussed  - Results are limited, with no p-values | This study describes a novel model of care that coordinates, allied health professional input to address patients’ and caregivers’ needs with the provision of a platform for high-quality discussions and subsequent communication to community teams. Significant increases in ACP discussions were observed, in conjunction with increased referrals to community courses and teams to address unmet care needs | Low |
|  |  |  |  |  | **Data collection** |  |  |  | **Rigour** |
|  |  |  |  |  | **Post-MDT cohort (n=46):**  EHRs of all patients discussed in the meeting were retrospectively analysed for patients’ care needs according to documented evidence.  **Pre-MDT cohort (n=26):**  Comparison cohort of patients who died 8 months prior to the introduction of MDT meetings. |  |  |  | Low |

| Author, country | Aim | Research design | Participants | Setting | Intervention | Outcomes | Limitations | Conclusion | Relevance |
| --- | --- | --- | --- | --- | --- | --- | --- | --- | --- |
| Beck et al.,  2016,  USA  [8] | To determine the attitudes of LT clinicians towards  PCC for ESLD patients awaiting LT  To identify any potential perceived barriers toward involving PC in these very ill patients. | Survey study between October 2012 and January 2013. | **N = 88**  LT clinicians:  - PGY1 physician trainees  - Nurse practitioners  - ICU nurses  - Fellows  - Attending physicians  **Inclusion:**  - cared for hospitalized LT patients over a one-year period from July 2011 to June 2012. | **Discipline:**  Hepatology (liver transplantation in ESLD)  **Hospital size:**  Not reported  **Hospital type:**  Academic center | **PCC** for ESLD patients awaiting liver transplantation. | **Barriers and benefits to PCC:**  - While one-third of attendings (38%) perceived that PCC was usually or always discussed on rounds, no nurses (0%) or PGY1 (0%) concurred ( p = 0.0001).  - Many respondents cited lack of clear criteria for involving PC services and difficulty prognosticating EOL.  - Nurses and PGY1 felt that it was not their place to suggest a consultation.  - Attendings were more likely than PGY1 and nurses to describe PCC services to their patients as EOL care ( p = 0.03).  - Attendings were least likely to perceive PCS as improving QOL  - Most participants reported that patients and families welcomed GoC discussions and were grateful for PCC  - The majority of respondents in all groups agreed that PCS helped the LT service provide higher quality of care to their patients. | - Only males participated, interpretation of results for females not possible.  - Small sample size  - 44% RR  - Possibility for recall bias. There may also have been a bias toward putting the liver transplant service in a better light. | Overall, few patients were referred for PCS despite the overwhelming recognition of the benefit of PCS by clinicians.  The main barriers to involving PCS in the care of LT patients were:  (1) confusion over optimal timing and referral criteria  (2) describing PC service as EOL care  (3) a lack of open dialogue on rounds. | Medium |
|  |  |  |  |  | **Data collection** |  |  |  | **Rigour** |
|  |  |  |  |  | Survey using a secure, web-based data capture tool. |  |  |  | Low |

| Author, country | Aim | Research design | Participants | Setting | Intervention | Outcomes | Limitations | Conclusion | Relevance |
| --- | --- | --- | --- | --- | --- | --- | --- | --- | --- |
| Berglund et al.,  2020,  USA  [9] | To describe the development of a social worker-led primary PC clinical model  To describe the patient population served by the program, rates of documented GoC discussions, and healthcare utilization of this population | Program evaluation with retro-spective cohort in 2017 | **N = 323**  Patients with serious illness admitted to hospital medicine  **Inclusion:**  - Receiving the intervention | **Discipline:**  Wide  **Hospital size:**  Large  (1144 beds)  **Hospital type:**  Quaternary-care teaching facility | **Social worker-led primary PC model** embedded within the hospital medicine service:  - model with specific aims  - model development through PC training and embedding of PC social worker  **Workflow:**  1. Screen at time of referral  2. Initial visit = exploration  3. Subsequent/ FU visit = alignment  4. Prior to discharge/closing visit = confirm | **Delivery pattern:**  - 14.7% (34) were referred directly by hospital medicine  - Patients were seen by the primary PC social worker a median of 3 days after admission  - After social worker PCC, 89% (207) patients had GoC discussions documented in the EHR, compared with 4.3% (10) before the consultation  **Healthcare utilization and discharge disposition:**  - 13% (29) were admitted to the ICU during the hospitalization, of which 22 occurred before the primary PCC and 7 occurred after the PCC  - 24.1% (56) were discharged with hospice  - 7.3% (17) were discharged with community-based PC services  - 10% (23) died during hospitalization  - 30 day readmission rate was 22 with a median of 11 hospital-free days | - retrospective study, single center  - Primary PC social work was unable to address physical symptom management needs and therefore referred 13% of patients to specialty PC  - Discussion reveals new information of what was not reported in results = confusing  - No data collected on willingness to discuss GoC and cost of the program.  **Strengths:**  - ‘Control’ group (see discussion) | This social worker-led primary PC program was feasible, expanded the reach of PC , increased GoC documentation, and maintained a hospice referral rate comparable with a specialty PC inpatient consultation service. | Medium |
|  |  |  |  |  | **Data collection** |  |  |  | **Rigour** |
|  |  |  |  |  | Data collected from the **hospital databases.** To evaluate the intervention **data was collected about:**  - clinical patient information  - primary PC utilization  - discharge disposition  - hospital-level demographics  - healthcare utilization data |  |  |  | Low |

| Author, country | Aim | Research design | Participants | Setting | Intervention | Outcomes | Limitations | Conclusion | Relevance |
| --- | --- | --- | --- | --- | --- | --- | --- | --- | --- |
| Böling et al.,  2020,  Sweden  [10] | To examine how PCC in hospitals are practiced, as perceived by consultants and health care professionals on receiving wards. | Focus group study with interpretative description from April 2016 through May 2018 | **N = 40**  **Healthcare professionals from PCTs:**  - Physicians  - RNs  - Social worker  **Healthcare professionals from wards receiving** **PCCs:**  - Physicians  - RNs  - Social worker  - Assistant nurse  **In/exclusion:**  Not reported | Four public hospitals within one Swedish region.  **Discipline:**  Not reported  **Hospital size:**  Medium -Large  (from 350-650 beds to 1.950 beds)  **Hospital type:**  - 1 University hospital  - 3 local hospitals | **PCC**  - No additional information reported | **1) Variations in PCC practice and its functions:**  - Most common way of conducting PCCs was to have a scheduled palliative round.  - Receiving wards occasionally were confused about the focus of PCC  - Factors affecting structure, process and outcomes of PCCs occur on different levels  - PCC services choose whatever methods of consultations they felt comfortable with or considered favorable from experience  - Perception of which professions should be included in the PCC varied  - PCC creates awareness and attention to PC  - PCC contributes to holistic approach, which is appreciated by HCWs  - PCC enhance cooperation between hospital wards and specialized PC organizations, which may create the prerequisite for safer and better prepared discharges.  **2) No ordinary consultation: employing a pro-active approach:**  - Confusion and lack of knowledge among non-specialized HCWs regarding the concept of PC. This appeared to influence what expectations they had of the palliative consultant/  - Staff turnover seen as an obstacle to reaching a consistent level of knowledge about PC and awareness of the PCC service.  - Importance of regular visits to the wards and a good relationship between PCT and the wards as prerequisites to receiving referrals and increasing the demand for PCCs  - A collaborative and attentive approach was seen as more conducive to facilitating good relationships than an overbearing one. | - 5 wards were contacted, 3 chose to participate in two focus groups, 1 preferred to contribute with written comments, 1 ward declined.  - Due to focus groups: Risk that potentially divergent views might have been left unsaid if perceived to be too controversial.  - participants from  the receiving wards were selected by their manager and some reported being part of a PC group, causing potential bias for a positive view of the consultations  **Strengths:**  - Purposive sapling seeking to include HCWqs from PCC services with different ways of conducting PCCs.  - Solid data enhances the credibility of this study.  - Differing professional backgrounds in different hospital contexts add to the represent-ativeness | Introducing PCC was a process whereby PCTs and receiving wards  successively found ways to collaborate. Important to such collaboration are shared views on  how PC is conceptualized as well as what a PCC can  contribute.  PCC were largely a bottom-up  driven development by PC specialists. Nevertheless, PCCs were  primarily perceived to make palliative needs visible, to contribute palliative knowledge, to  strengthen cooperation between specialized PC and hospital care, and to create  opportunity to ameliorate transition. | High |
|  |  |  |  |  | **Data collection** |  |  |  | **Rigour** |
|  |  |  |  |  | Focus group discussions with staff from PCTs and wards receiving PCCs, separately.  **Total focus groups (n=11):**  - Consultation service A: key informant (n=4)  - Consultation service B (n=1)  - Consultation service C (n=1)  - Consultation service D (n=1)  - Consultation service managers (n=2)  - Receiving ward A (n=1)  - Receiving ward B + C (n=1)  **Contributed with written comments (n=1):**  - Receiving ward D |  |  |  | High |

| Author, country | Aim | Research design | Participants | Setting | Intervention | Outcomes | Limitations | Conclusion | Relevance |
| --- | --- | --- | --- | --- | --- | --- | --- | --- | --- |
| Braus,  2016,  USA  [11] | To evaluate  the effects of a palliative care intervention on clinical and family  outcomes, and palliative care processes. | Prospective, before-and-after interventional study from June 2013 to June 2014. | **N = 203**  Patients admitted to the medical critical care service.  **Exclusion**:  - awaiting a solid organ transplantation | **Discipline:**  Intensive care  **Hospital size:**  Large  (566 beds)  **Hospital type:**  Academic medical center with 24-bed ICU | A member of the PCC team was **relocated** from the PCU to the ICU | **1) Proportion of patients with a family meeting:**  - significantly higher likelihood of a documented family meeting (p=0.001)  **2) Time between ICU admission and the occurrence of a family meeting:**  - significantly shorter (p<0.001)  **3) ICU LoS:**  - Not significantly different.  **4) Hospital LoS:**  - Significantly shorter (p<0.001)  **5) ICU mortality:**  - Not significantly different.  **6) In-hospital mortality:**  - Not significantly different  **7) Family satisfaction with care**:  - Similar between groups (p=0.52)  **8) Burden of psychological symptoms experienced by family members:**  - No significant differences between groups. | This study was underpowered to detect change in family-reported outcomes. The use of self-administered surveys delivered by mail may result in nonresponse bias and may limit the willingness of respondents to disclose sensitive information about psychological symptoms.  Unclear what aspect of the intervention was effective | Proactive PC involvement on ICU rounds for high-risk  patients to prompt ICU physician attention to PC needs  and to nudge the ICU physicians to better address these  needs was associated with more and earlier ICU family  meetings and shorter hospital LoS**.** | Medium |
|  |  |  |  |  | **Data collection** |  |  |  | **Rigour** |
|  |  |  |  |  | **Usual care phase** (n=100)  - Investigators identified patients with one or more of the prespecified clinical trigger criteria, but did not inform the clinical treating team.  **Intervention phase** (n=103)  - Investigators identified patients with one or more of the prespecified clinical trigger criteria and informed the relocated PC clinician.  - The PC clinician reviewed the EHR, informed the medical team and participated in interdisciplinary morning bedside ICU rounds.  - On subsequent days, the PC clinician would make suggestions about addressing PC needs, including recommending interdisciplinary family meetings when appropriate. |  |  |  | High |

| Author, country | Aim | Research design | Participants | Setting | Intervention | Outcomes | Limitations | Conclusion | Relevance |
| --- | --- | --- | --- | --- | --- | --- | --- | --- | --- |
| Cannon et al.,  2022,  USA  [12] | To describe the palliative care consultation practices in an academic head and neck surgery practice. | Retrospective review study from February 2019 to October 2020. | **N = 10**  Patients with advanced head and neck cancer.  **Inclusion:**  - received PCC  **Exclusion:**  - No diagnosis of advanced head and neck cancer. | **Discipline:**  Oncology, head and neck surgery service  **Hospital size:**  Not reported  **Hospital type:**  Not reported | **PCC** | **1)** **Time of admission to PCC: 9days**  **2) Median LoS: 13.5days**  **3) Timing of PCC**:  - Preoperatively (n=1)  - Transitioned care (n=1)  - Average of 8 days post-operatively (n=8)  **4) Location of PCC:**  - ICU (n=7)  - Surgical floor (n=3)  **5) Mortality rate: 90%**  - Died at home (n=4)  - Died in ICU (n=2)  - Died in hospice facility (n=1)  - Died in long-term care hospital (n=1) | - Small sample size over long period of time  - Retrospective study with no form of control group | The advanced head and neck cancer team consulted PC on 12% of patients with advanced head and neck cancer while on the otolaryngology service.  PCC was commonly late  but associated with overall treatment de-escalation, emphasis on patient  values, and goal-congruent care. | Medium |
|  |  |  |  |  | **Data collection** |  |  |  | **Rigour** |
|  |  |  |  |  | Review of a PC database and the EHR for all PCC of patients suffering from advanced stage head and neck cancer within a 21-month period. |  |  |  | Low |

| Author, country | Aim | Research design | Participants | Setting | Intervention | Outcomes | Limitations | Conclusion | Relevance |
| --- | --- | --- | --- | --- | --- | --- | --- | --- | --- |
| Connolly et al.,  2021,  Ireland  [13] | To evaluate the role of the SPC CNS in an acute hospital setting | Mixed-methods study with sequential explanatory design | **N = 121**  (Phase 1)  - Hospital managers  - Nurses  - Nurse managers  - Doctors  - Allied health professionals  **N = 10**  (Phase 2)  **In/exclusion:**  Not reported | **Discipline:**  Wide  **Hospital size:**  Not reported  **Hospital type:**  Not reported | **SPC CNS role:**  - SPC nurses = reference point for clinical advice and guidance.  - CNS involvement in general practice, counseling staff, and providing clinical leadership through consultative role and is in regular contact with nursing and hospital management. | **PHASE 1:**  - 53.7% had referred a patient to the SPC CNS in the past 12 months  **Reasons for advice sought from SPC CNS:**  - 71% symptom management  - 15.7% education of staff  - 9.1% staff support  **Few respondents had any collaboration with the SPC CNS concerning:**  - audit (5%)  - research (2.5%)  - quality improvement (7.4%)  **PHASE 2:**  **1. Role of SPC CNS:**  - varied understanding  - educative, supportive  - sense of continuity of care  - a resource to support the care of patients with complex needs | - Majority of participants were nurses. An increased response from other HCWs would have added to the results.  - Despite encouragement and planning, the number of participants who were available to attend focus groups was fewer than anticipated  - Lack of patient’s voice in the evaluation. | No definition of the SPC CNS role exists in practice and this vacuum creates differences in expectations and perceptions of care provision.  The SPC CNS service should be fully embedded in the hospital including the provision of infrastructure to support the clinical, advocacy, consultation, education, audit and research work of the SPC CNS. | High |
|  |  |  |  |  | **Data collection** |  |  |  | **Rigour** |
|  |  |  |  |  | **Phase 1: QUANT**  - anonymized **survey** of key stakeholders  **Phase 2: QUAL**  **- Focus groups (n=6)** with multi-professional staff who had completed the questionnaire  - Additional **individual interviews (n=4)** with key stakeholders  - Interview schedule guided by questions from the survey and informed by initial quantitative analysis. |  |  |  | High |

| Author, country | Aim | Research design | Participants | Setting | Intervention | Outcomes | Limitations | Conclusion | Relevance |
| --- | --- | --- | --- | --- | --- | --- | --- | --- | --- |
| Courtright et al.,  2020,  USA  [14] | To understand hospitalists’ decision-making about PCC  for patients with dementia and to describe factors that influence their  likelihood of referral. | Descriptive qualitative study between January and July 2018. | **N = 28**  Hospitalist physicians  **Inclusion:**  - Valid e-mail | Seven hospitals within **a national nonprofit health system** across seven states.  **Discipline:**  Not reported  **Hospital size:**  Not reported  **Hospital type:**  - 2 teaching hospitals  - 5 community hospitals | An established **PC program** ranging in duration from 5 to 18 years. | **Facilitators for PCC**  **1) Patient factors**  - advanced disease  - receipt of aggressive interventions  **2) Family caregiver factors**  - Lack of understanding the disease-trajectory  - Intra-family disagreements  **3) Hospitalist factors**  - Insufficient time  **4) Organization factors**  - response of PCT in timely manner  **Barriers for PCC**  **1) Patient factors**  - quick recovery  **2) Family caregiver factors**  - Limited family presence or involvement  - Family resistance  **3) Organization factors**  - busy PCT | - Not sure if full range of perspectives was captured, as hospitalists with less favorable views might be less likely to participate.  - Unable to explore if or how variation in the volume of patients with dementia that hospitalists cared for influenced the consultation decision process | Hospitalists described their decision process for and barriers to PCC for patients with dementia, based on patient, family, hospitalist and organizational factors. Therefore, this study provides an in-depth framework of hospitalists’ decision-making regarding PC referral for PCC. | Medium |
|  |  |  |  |  | **Data collection** |  |  |  | **Rigour** |
|  |  |  |  |  | Individual, semi-structured, telephone interviews to explore factors that influence hospitalists’ decision to consult PC for patients with dementia.  A trained interviewer conducted all interviews using a standardized guide. Median interview duration was 26 minutes.  An iterative process of thematic analysis was used to synthesize the data, identify patterns, and develop themes across interviews. |  |  |  | High |

| Author, country | Aim | Research design | Participants | Setting | Intervention | Outcomes | Limitations | Conclusion | Relevance |
| --- | --- | --- | --- | --- | --- | --- | --- | --- | --- |
| Coym et al.,  2020,  Germany  [15] | To explore physicians’  perceptions on the impact of PCC, its triggers, challenges and limits, and their suggestions for future service  improvements | Multi-perspective qualitative study between January 2017 and May 2018 | **N = 19**  **PC physicians** (n=10)  **Inclusion:**  - Provision of PCC on a regular basis within the last 12 months  **Requesting physicians** (n=9)  **Inclusion:**  - Provision of day-to-day care for inpatients with advanced, life-limiting diseases  - Having requested PCC regularly within the last 12 months | **Discipline:**  Intensive care, oncology, gynecology, dermatology and nephrology  **Hospital size:**  Large  (1.700 beds)  **Hospital type:**  Maximum care hospital with about 500.000 treated patients/year | **Inpatient PCC**  - Service since 2008  - Team with physicians and nurses since 2017  - Available throughout the whole hospital and all departments via an online form in the patient’s EHR | **Issues leading to PCC request**  - Physical symptom burden  - Over-straining  - Organization of further care  - Decision-making  - Change of therapeutic goal  - Limited staff resource  **Impact of PCC:**  - Transfer of knowledge  - Relief  - Better patient coping  - Improvement in symptom burden and further care  **Barriers for request, conduct and implementation:**  - Refusal  - Resistance  - Limited time  - Lack of knowledge  - Assumption of missing benefit  - No adequate setting | - It is not reported if there was saturation.  - Single institution, thus transferability of findings is limited.  - Requesting physicians were known for including PCC in their treatment. So, they generally have a positive attitude towards PC. This could lead to bias. | Findings show that there is a gap between the  requesting physicians’ idea of why and how they might  need support and the PC specialists’ ideas. PCC showed positive effects in supporting physicians, but are also limited due to  structural problems, lack of knowledge, insecurity, and  skepticism by the requesting physicians. At the same  time, more resources for both teams are needed to ensure continuity for patient treatment and further care. | High |
|  |  |  |  |  | **Data collection** |  |  |  | **Rigour** |
|  |  |  |  |  | Following interviewer training and a pilot interview, face-to-face, semi-structured interviews were conducted by 3 female interviewers, who were neither involved in PCC nor known by the interviewee.  Semi-structured interview guide with open-ended  Qualitative content analysis with inductive coding approach. |  |  |  | High |

| Author, country | Aim | Research design | Participants | Setting | Intervention | Outcomes | Limitations | Conclusion | Relevance |
| --- | --- | --- | --- | --- | --- | --- | --- | --- | --- |
| De Meritens et al.,  2017,  USA  [16] | To describe practice patterns, attitudes, and barriers to the integration of  PC services by gynecologic oncologists. | Observational  survey study  Jul - Aug 2015 | **N = 145**  Gynecologic oncologists which were members of the SGO  **In/exclusion:**  Not reported | **Discipline:**  Gynecologic oncology  **Hospital Size:**  Not reported  **Hospital type:**  - University hospitals (n=85)  - Community hospitals (n=17)  - Hybrid practice types (n=32)  - Private practices (n=9)  - Other (n=3) | **PC services** | **Demographics:**  - Most respondents worked in a university-affiliated hospital (58%)  **Reasons to consult PC**  - symptom control (75%)  - life expectancy less than 6 months (42%)  - Time of diagnosis (17%)  - Few were ‘very likely’ to consult PC providers to communicate prognosis (9%), DNR status (10%), or discuss GoC (21%)  **Competence in PC**  - Respondents perceived themselves ‘very competent’ to discuss prognosis (86%), DNR status (91%) and GoC (82%). This is consistent with not being ‘very likely’ to consult PC for these tasks = statistically significant negative association (p<0.001)  **Perceived benefits of PCC:**  - Transition to EOL-care (95%)  - Grief counseling (87%)  - Spiritual support (81%)  - Reduction of futile interventions (74%)  - Discussions of GoC (73%)  - Communication of prognosis (54%)  **Barriers to consulting PCT:**  - Fear that family would assume oncologist was giving up on the treatment (90%)  - Family resistance (80%)  **5) Not seen as significant barriers:**  - Lack of availability (22%)  - Lack of timely access (20%)  - Fear of increasing length of hospital stay (11%)  - Prior inpatient conflict with the palliative care team (11%)  **6) Feelings regarding collaboration**  - Gynecologic oncologists overwhelmingly  (97%) reported that PCTs are a useful adjunct to patient care.  - 21% of respondents reported that they have had a negative experience with a PCT that has made them reluctant to consult the team in the future.  - 30% reported that they either often or sometimes feel reluctant to call a PCC for a patient for whom they would have otherwise called a consultation. | - No data from PC specialists perspective was obtained.  - Low RR (20%).  - Possible selection bias  - Limited generaliz-ability  - Cross-sectional study so no follow-up questions possible. | PC services are seen as a useful adjunct to  patient care, yet they are underused.  The main barrier for consulting PC  specialists is the perception of the gynecologic oncologist  that patients and their families will feel abandoned.  Respondents demonstrated interest in PC collaboration for symptom control and EOL discussions, which can  serve as the impetus for early referral. | Low |
|  |  |  |  |  | **Data collection** |  |  |  | **Rigour** |
|  |  |  |  |  | 27-item survey to evaluate practice patterns, attitudes, and barriers to the incorporation of PC services.  The survey was piloted in attending physicians at Colombia and reviewed by members of the gynecologic oncology and palliative care divisions at the institution. After ethical approval the survey was sent to all members of the SGO who had e-mail addresses available in the SGO directory. |  |  |  | Low |

| Author, country | Aim | Research design | Participants | Setting | Intervention | Outcomes | Limitations | Conclusion | Relevance |
| --- | --- | --- | --- | --- | --- | --- | --- | --- | --- |
| Economos et al.,  2020,  France  [17] | To explore the physicians’ perceptions of palliative care and factors influencing reasons to refer to specialist PC. | Qualitative study  Nov 2014 - Jun 2015 | **N = 18**  Physicians  **Inclusion:**  - Medical oncologists  - Medical specialists working in the oncology field  - Access to PC  **Exclusion:**  - No clinical involvement  - Pediatric oncologists  - Hemato-logists  - Surgical oncologists  - Radio-therapists | **Discipline:**  Oncology  **Hospital size:**  Not reported  **Hospital type:**  - Tertiary university hospitals (n=2)  - Regional cancer center (n=1) | Access to PC through the existence of **a mobile PCT** in the institution | **1) Most** **important benefit of PC interventions**: alleviation of patient’s symptoms  **2) Triggers for referral:**  - Distressing symptoms  - Worsening of clinical status  - Greater experience and skills in PC  - Feeling overwhelmed  - Psycho-social support  - Multi-disciplinary approach  **3) Key points in collaboration:**  - Absence of judgement  - Real dialogues | **Limitations:**  - Sample was mainly young physicians, so results might reflect only the point of view of a selected population.  - Exploration of facilitators and barriers might be subject to recall bias or cognitive distortions.  - Results might not be reflecting practices from non-academic public hospitals.  **Strengths:**  - interviews were performed until data saturation.  - interviews carried out by people not directly involved in the studied fields. | PC referral was perceived as helping in  addressing communicational and decision-making  issues, as well as in helping to manage refractory symptoms. | Medium |
|  |  |  |  |  | **Data collection** |  |  |  | **Rigour** |
|  |  |  |  |  | Semi-structured, face-to-face, individual interviews guided by an interview guide to explore physicians’ perceptions of PC and their opinion on how to improve collaboration between clinicians in PC and oncology. |  |  |  | High |

| Author, country | Aim | Research design | Participants | Setting | Intervention | Outcomes | Limitations | Conclusion | Relevance |
| --- | --- | --- | --- | --- | --- | --- | --- | --- | --- |
| Firn et al.,  2018,  USA  [18] | To explore and develop a theory of ward social workers’ perceptions of what facilitates or hinders collaboration with PC social workers in the hospital. | Qualitative study with grounded theory design from February 2014 through January 2015. | **N = 14**  Masters trained social workers  **Inclusion:**  - English speaking  - working with adult patients  **Exclusion:**  - Working at military or children hospitals  - Working at hospitals without PC social worker  - Social work students  - Not masters trained  - PC social workers | 6 hospitals located in the State of Michigan.  **Discipline:**  All areas of inpatient wards  **Hospital size:**  Medium to Large  (300 – 1100beds)  **Hospital type:**  For-profit and non-for-profit hospitals.  2 small community hospitals  2 larger urban hospitals  2 inner city hospitals | **Interprofessional collaboration** between generalist social workers and specialist social workers | **1. Trust**  ***Ability:***  - Perceptions of the PC social worker’s abilities impact the word social worker’s willingness to collaborate  - When PC social worker is perceived to be a strong, capable clinician, trust is high and collaboration is facilitated.  ***Benevolence;***  - Ward social workers identify PC social workers as demonstrating benevolence towards them when they are helpful, supportive, respecting of skills, and sharing the burden of caring.  - Lack of benevolence undermines trust and collaboration  ***Integrity***  - The ward social worker does not trust or want to collaborate with the PC social worker who fails to uphold patient-centered values. Lacking integrity is a barrier to collaboration.  **2. Information Sharing**  - For communication to work it must be two-way, timely and consistent.  - When communication is lacking, delayed, inconsistent, or perceived as burdensome to the patient and family collaboration is obstructed.  **3. Role Negotiation**  - No formal organization differentiation between the ward and PC social worker skill sets and roles.  - Comfort levels also contribute to how roles are negotiated  - Negotiation decisions are made based on time, comfort, institutional priorities, and therapeutic relationship with the patient.  - Willingness to be flexible about which social workers does what task is essential for effective role negotiation.  - When role negotiation does not take place or when it fails, collaboration cannot be achieved. | The theoretical model may change or expand if respondents from other States, countries, ethnicities , and cultures were included.  Social work managers served as gatekeepers for accessing the line staff, they may have introduced bias by the way they identified or requested participants.  Participants who had a predominantly positive or a predominantly negative view of collaborating with specialist PC social workers may have chosen to participate, thereby skewing the data in a particular direction. | The key constructs of collaboration are: Trust, Information Sharing, and Role Negotiation.  When all three constructs are in place and operating well, the ward social worker perceives interactions with the PC social worker as collaborative. When one or more pieces are missing the ward social worker does not experience interactions with the PC social worker as collaborative. | High |
|  |  |  |  |  | **Data collection** |  |  |  | **Rigour** |
|  |  |  |  |  | In-person interviews with open-ended question from an interview guide. |  |  |  | High |

| Author, country | Aim | Research design | Participants | Setting | Intervention | Outcomes | Limitations | Conclusion | Relevance |
| --- | --- | --- | --- | --- | --- | --- | --- | --- | --- |
| Friedrichsen et al.,  2021,  Sweden  [19] | To study the meeting and interaction of two different organizational care cultures, PC and acute care, when a PCT introduces consulting services to acute wards regarding end-of-life PC. | Pheno-menological interview study  2010 - 2012 | **N=136**  Team members of acute care wards:  - Physicians  (n=21)  - Nurses  (n=73)  - Assistant nurses (n=39)  - Others (n=3)  **In/exclusion:**  Not reported | **Discipline:**  Internal medicine and surgical care  **Hospital size:**  Not reported  **Hospital type:**  Local hospital with 2400 employees | **PCT was available for**  **1 – 8 daytime hours/week for 1 year.**  **Tasks:**  - Visiting the wards and taking part in reports, rounds, and communications.  - Identifying patients at risk for poor outcomes.  - Supporting primary healthcare team members.  The primary team could ask for specific education for all team members in the wards. | **1) Anticipation meets reality:**  - Primary team members had different expectations about the PCT.  - Nurses and assistant nurses expected that the PCT would give them support in their thinking about PC.  - Physicians have a lack of knowledge or skills  **2) Valuation of time and prioritizing:**  - hectic ward environment  - value of fast work because of lacking time  - Hesitant for use of tools/scales to identify and measure PC symptoms because they are time-consuming.  **3) The content and creation of PC:**  - Content of PC is interpreted differently  - primary care team wants to do as much as possible before ‘giving up’  - fear of being criticized for making wrong decisions regarding PC  - fear of not having done enough. | Possibility of having other cultures on internal medicine wards and surgical wards.  No other limitations of the study discussed. | the PCT and acute care have different knowledge and skills regarding PC and there is resistance regarding PC in the acute wards when it comes to changing routines.  The challenges for the intervention were: primary team members’ views of skills regarding  PC, their internal values as lack of time as well as the  content of PC. This led to a collision between the PCT and them, as PCT noticed the lack of a holistic view on dying patients. | High |
|  |  |  |  |  | **Data collection** |  |  |  | **Rigour** |
|  |  |  |  |  | **- Participatory observations**, which focused on almost all situations where the PCT were involved but not when working with patients or family members.  **- Interviews,** which lasted between 3min to 1,5 hour.  **- Diary entries** written by the PCT during 1 year. |  |  |  | High |

| Author, country | Aim | Research design | Participants | Setting | Intervention | Outcomes | Limitations | Conclusion | Relevance |
| --- | --- | --- | --- | --- | --- | --- | --- | --- | --- |
| Gatta et al.,  2018,  USA  [20] | To explore the beliefs and practices of medical ICU physicians in regard to providing an integrative model of PC in the medical ICU. | Semi-structured interview study | **N = 17**  Fellows and attending physicians from the medical ICU  **Inclusion:**  - having served in the medical ICU in the past year  - being available for a 30- to 60-mintue in-person interview | **Discipline:**  Intensive care  **Hospital size:**  Not reported  **Hospital type:** Academic hospital and its affiliated Veterans Affair hospital. | **PCTs** consisting of physicians, nurse practitioners, nurses and social workers. | **1) Embracing primary PC**  - Each participant believed that providing PC was a part of their practice.  **2) The added value of the PC consultant**  - Participants recognized PC consultants as a comprehensive team that could provide both acutely needed services and long-term value.  **3) Motivations for moving from primary PC to initiating PC consultations**  - To provide satisfactory PC, the clinician needs to have time. Time was the most frequently mentioned motivation for obtaining a PCC. | - Does not report extensively on interaction processes and PCT components (see checklist).  - Limited generaliz-ability of results.  - Future interview studies should include other stakeholders to better understand which PC interventions might be most successful for each institution. | Meeting the PC needs of critically ill patients clearly requires  cooperation between specialist PC providers and non-specialist  clinicians. This integrative model is most likely to be successful after considering the particular needs and culture of an  individual institution. | Medium |
|  |  |  |  |  | **Data collection** |  |  |  | **Rigour** |
|  |  |  |  |  | **Audio-recorded, semi-structured interviews about:**  - The meaning or content of PC.  - How PC is currently practiced.  - Potential hesitancies about PC. |  |  |  | Low |

| Author, country | Aim | Research design | Participants | Setting | Intervention | Outcomes | Limitations | Conclusion | Relevance |
| --- | --- | --- | --- | --- | --- | --- | --- | --- | --- |
| Hill et al.,  2022,  USA  [21] | To assess the clinical and financial outcomes associated with an inpatient PC clinical pharmacy specialist on  an inpatient PCT | Retrospective cohort study between October 1, 2016 and February 28, 2019 | **N = 1543**  Hospitalized patients  **Inclusion:**  - Inpatient stay  - PCC  - > 6months KPCO membership before index date  **Exclusion:**  - Patients with outpatient PC consult during the 180-day FU period | 4 contracted hospitals of the KPCO  **Discipline:**  Not reported  **Hospital size:**  Not reported  **Hospital type:**  Not reported | **PC clinical pharmacy specialist** on inpatient PCT:  - acts as liaison between other clinical staff and PCT’ is one.  - Has several essential and/or common responsibilities  - Service provided in-person, telephonically, or electronically | Intervention group had a greater median change in - Daily total healthcare expenditures (p=0.003)  - 180-day change in daily morphine milligram equivalents (p=0.007)  Intervention group had a higher mean of LoS (p=0.003) but lower **rates of:**  - hospitalization (p=0.010)  - urgent care visits (p=0.024)  And lower **counts of:**  - hospitalizations (p=0.010)  - urgent care visits (p=0.002) | Patients with less symptom burden may have been more likely to be in the control group since PC pharmacists may have been consulted on patients with a higher symptom burden.  Study conducted in one integrated healthcare delivery system.  Missed or incorrect events may have led us to underestimate true event rates. | This retrospective analysis of patients who received inpatient PC services identified that the addition of an inpatient PC pharmacist specialist  to a PCT was associated with decreased health care expenditures and increased pain management without an increase in opioid-related adverse events. | Low |
|  |  |  |  |  | **Data collection** |  |  |  | **Rigour** |
|  |  |  |  |  | **Intervention group (n=228):** PC from clinical pharmacy specialist  **Control group (n=1315):** Usual PC  **EHR** medical, pharmacy, and membership administrative records were used to identify patients, treatments, and outcomes. |  |  |  | Low |

| Author, country | Aim | Research design | Participants | Setting | Intervention | Outcomes | Limitations | Conclusion | Relevance |
| --- | --- | --- | --- | --- | --- | --- | --- | --- | --- |
| Huang et al.,  2019  Taiwan  [22] | To explore any correlations between healthcare staffs’ knowledge, attitudes, and practices regarding PCC | Cross-sectional study from June 1, 2018 to September 30, 2018. | **N = 210**  Health care staff:  - Physicians and nurse practitioners (n=39)  - Nurses (n=140)  - Other (n=31)  **Inclusion:**  - 20 years or older  - Working in ICU or in the ward  - Able to speak Mandarin/Taiwanese  **Exclusion:**  - Unable to provide informed consent  - Cognitive impairment | **Discipline:**  Intensive care, internal medicine, surgery, obstetrics and pediatrics  **Hospital size:**  Not reported  **Hospital type:**  Regional, teaching hospital | **PCC service:**  - encourages collaboration between PCT members and medical team members and is comprised of physicians, nurse specialists, social workers, psychologists, pharmacists, dietitians, religious workers, and nursing staff.  - Provides PC advice to primary health professionals according to the patient’s condition. | - More knowledge towards PCC service engendered more favorable practices and more positive attitudes towards PCC services  (p<0.01)  - More positive attitudes towards PCC services indued more favorable practices towards PCC services (p<0.01)  **Predictors of practices toward PCC services:**  - Knowledge towards PCC services  - Attitudes towards PCC services  - Experience of a family member(s) or friend(s) passing away  - occupation category  (p<0.001) | - Not specified in which hospital wards the health care staff was working.  - High RR 31.3%. However, survey was distributed by researcher himself, can cause bias? | Four predicators for practices towards PCC services were found. In addition, health care staff’s knowledge and attitudes towards PCC services are positively correlated with their practices towards PCC services. | Low |
|  |  |  |  |  | **Data collection** |  |  |  | **Rigour** |
|  |  |  |  |  | **Structured, self-reported questionnaire KAP-PCCSI** including:  1) Knowledge: 15-items  2) Attitude: 10 items  3) Practice: 10 items |  |  |  | Low |

| Author, country | Aim | Research design | Participants | Setting | Intervention | Outcomes | Limitations | Conclusion | Relevance |
| --- | --- | --- | --- | --- | --- | --- | --- | --- | --- |
| Jackson & Minick  2016,  USA  [23] | To examine the experiences of hospitalist physicians as it related to a hospital’s PC program with the following research questions:  (a) What are hospitalist physicians’ experiences with PC?  (b) What does it mean to refer patients to PC ?  (c) What barriers and facilitators do physicians experience with PC referral?  (d) What patient characteristics are associated with PC referrals? | Qualitative study with interpretative pheno-menology design | **N = 6**  Hospitalist physicians  **Inclusion:**  - referred patients to PC services > 3 times in the last year.  **Exclusion:**  - specialists/ consultants (cardiologists, nephrologists, surgeons, etc.) | **Discipline:**  Not reported  **Hospital size:**  Not reported  **Hospital type:**  Community hospital  - 24h emergency services along with major medical, surgical and diagnostic care | **PC program**  - Established for 5 years  - Staffed by 1 collaborating physician (off hospital site) and 3 nurse practitioners (on hospital site) | **1) When hospitalists physicians want help**  Hospitalist physicians consulted PC when they needed help when:  - They could not do it any more  - GoC needed to be renegotiated  - Time was needed to listen  - Pain management was desired  **2) Barriers to referral:**  - Family refusal  - Oncologists being a barrier to hospitalist physicians initiating a PC referral  - Meaning of ‘palliative’ | - Single center  - Small sample  However, participants’ spread between ages, years of being a hospitalist physician, and  years of practicing at the study site did provide a reasonable representation of hospitalist physician  - Participants had referred many patients to PC before, so they had more positive attitudes toward PC than other hospitalist physicians  - No participant demographic table | This study has shown that hospitalist physicians who have  referred patients to PC generally find these services helpful and save them time.  This study also revealed that the need for renegotiating patients’ goals, time to listen, and pain management facilitated PC referrals,  whereas biases from patients, families, and other physicians about PC and the meaning of ‘‘palliative’’  seemed to be the greatest barriers to referral. | Low |
|  |  |  |  |  | **Data collection** |  |  |  | **Rigour** |
|  |  |  |  |  | Individual face-to-face interviews lasting about 30-60 minutes.  Field notes taken during each interview  Open-ended interview question:   - Can you tell me about a time when you referred a patient to PC? - How do you decide if and when to refer patient(s) to PC? - Can you tell me about a time when it was difficult to initiate a PCC for one of your patients? |  |  |  | Low |

| Author, country | Aim | Research design | Participants | Setting | Intervention | Outcomes | Limitations | Conclusion | Relevance |
| --- | --- | --- | --- | --- | --- | --- | --- | --- | --- |
| Jacobsen et al.,  2017,  USA  [24] | To address PC workforce shortages by teaching clinicians how to provide primary PC through peer coaching  To describe the pilot coaching intervention and report on participant perceptions of usefulness of the approach and lessons  learned. | Pilot quality improvement study over 6 weeks | N = 28  Health professionals:  - Internal medicine residents  - Attending physicians  - Nurse practitioners  - Physician assistants  **In/exclusion:**  Not reported | **Discipline:**  Not reported  **Hospital size:**  Large (1000beds)  **Hospital type:**  Urban, academic medical center | **Peer coaching** **program** for PC-related questions.  - Inpatient service delivery model  - Offered to clinicians  - Coaches (n=5): PC attending physicians with clinical practice > 2 years  - Coaching approach:  ° speaking with coachee  ° brief meeting with  patient  ° teaches the coachee  the needed skill or  information,  ° Joint visits if needed  ° Recommendations  were documented | **28 coaching encounters on 24 patients**  **32 different learning goals about:**  - Pain and symptom management (44%)  - Communication (34%)  - Hospice (22%).  **Coaching encounters resulted in:**  - Symptom management medication recommendations (50%)  - Teaching of communication skills (39%)  - Assistance  with care transitions (29%)  - A joint visit with the coach, learner, and patient (21%)  - Recommendation for traditional PCC or consult with different service (11%)  **Usefulness of peer coaching:**  - Easy access to expertise  - Tailored teaching  - Being in partnership  **Point for improvement:**  - Make it easier to figure out who is the coach | - Not clear how many nurse practitioners and physicians assistants as they are reported as “mid-level providers”  - Single Center study with small sample  - No control  - Future work is needed to define fidelity of the intervention across consults | Peer coaching at care delivery does two things:  1) supports clinicians as they build primary PC skills  2) creates a new model of  consultation that extends the PC workforce. | High |
|  |  |  |  |  | **Data collection** |  |  |  | **Rigour** |
|  |  |  |  |  | **Audit** of peer coaching encounters and coachee feedback.  **Primary outcome:** number of coaching encounters  Process measures and descriptive data.  **Qualitative data** about coachee perception of usefulness were collected through REDCap Surveys. |  |  |  | High |

| Author, country | Aim | Research design | Participants | Setting | Intervention | Outcomes | Limitations | Conclusion | Relevance |
| --- | --- | --- | --- | --- | --- | --- | --- | --- | --- |
| Kawabata & Nin,  2021,  Japan  [25] | to examine whether periodic assessments of PCTs from attending physicians and ward nurses, make the teams more effective. | Two-step quantitative survey research in from October 2019 to March 2019 | **N = 519**  Physicians and nurses from all hospital wards  **Inclusion:**  - Physicians had a history of PCT requests  **Exclusion:**  - Physicians from the departments: radiology, orthopedics, ophthalmology, dermatology, pediatrics, and rehabilitation | **Discipline:**  Wide  **Hospital size:**  Large  (678 beds)  **Hospital type:**  Not reported (Osaka Rosai Hospital) | **PCC**  - Provide assistance for symptom management, establishing care goals, treatment decisions, ACP, and other issues associated with living with serious illness. | **1) Beliefs and experiences with PCTs:**  - Physicians who recognized the PCT as a useful consultant experienced troubles in symptom management, felt free to request the PCT, and had required workshops about PC.  - Ward nurses who had easily accessed the PCT had found them helpful.  **2) Needed improvements identified from first (baseline) survey:**  - Publicity of PCT and PC for client candidates were insufficient. Although user satisfaction was high (91.7%)  - Difficulty in requesting the PCT (11.3%)  **3) Strategies to address these issues:**  - Bimonthly internal newsletters on PC and the PCT activity  - Daily rounds to the customers for in-person communication and acquaintance.  **4) Results of the improvements (survey 2):** - No significant changes in a notice regarding changes in PCT activities  - No significant changes in easy accessibility to the PCT for physicians. However ward nurses, did show an improvement. | - Sample size at baseline is smaller than sample size after 5 months. More participants after 5 months, so RR improved. Is this a good thing? Or does this make results difficult to interpret?  - Reproduc-ibility and reliability of the results obtained using a non-standard questionnaire which was constructed specifically for this PCT in this specific context ??  - Solutions to address problems uncovered by surveys cannot be perfect. | Statistical improvements over 5 months in accessing the PCT application regulated by the government. In addition, ward nurses indicated signs of easier access to the PCT. No change in other answers to the second questionnaire worked as non-interventional control. | Medium |
|  |  |  |  |  | **Data collection** |  |  |  | **Rigour** |
|  |  |  |  |  | Characteristics of the clients of the PCT and issues needing improvements were collected through a questionnaire at:  **- Baseline:**  Physicians (n=101)  Ward nurses (n=394)  **- 5 months later:**  Physicians (n=115)  Ward nurses (n=404)  In between surveys new strategies and tactics were developed to resolve the problems highlighted in survey 1.  Duration of both data-collection periods:  2 weeks. |  |  |  | Low |

| Author, country | Aim | Research design | Participants | Setting | Intervention | Outcomes | Limitations | Conclusion | Relevance |
| --- | --- | --- | --- | --- | --- | --- | --- | --- | --- |
| Kennedy et al.,  2019,  USA  [26] | To assess the views of nurses, resident doctors, and attending physicians of the use of a readily available pain and PC specialty at their institution while assessing their ability to recognize terminal noncancer illnesses | Cross-sectional survey study | **N = 90**  - Internal medicine residents (n=31)  - Nurses (n=49)  - Nurse practitioners (n=2)  - Internal medicine attending physicians (n=8)  **Exclusion:**  - Nurses and attending physicians who were pain and palliative specialists | **Discipline:**  Internal medicine ward, ICU, and cardiac medical unit  **Hospital size:**  Not reported  **Hospital type:**  Community hospital | **Referral to the PC service**  - In addition to direct consult requests by a primary attending, nurses are allowed to make referrals to the onsite PC service. These referrals are then made into official consults after agreement by the primary attending. | **Reasons for referral/consult:**  - Hospice care  - Terminal cancer  - Uncontrolled pain  - Regarding situations where noncancer diseases deemed adequate for referral/consultation responses were variable!  **Most appropriate time for PC/referral:**  - Most frequent response: ‘at the point of diagnosis of terminal illness’  **Benefit of PC service in acute care:**  - 45,2% of residents, 75% of nurses and 50% of attending physicians disagreed to some degree that there is very little benefit.  **Reasons for delay in consult:**  - PC service too aggressive in withdrawal of care  - No involvement of primary team in decision-making  **Barriers to effective service:**  - Primary attending reluctance to consult  - Late consultation/referral  - The belief that PC means discontinuation of live-saving measures  - Patients’/relatives’ lack of understanding PC  **PC service utilization and recognition:**  - 75% of physicians were unsure whether PC service was adequately used  -44% of nurses disagreed that it its adequately used  - 32% of residents disagreed that it is adequately used | - Single center  - Small sample size  - Reporting of data-collection is insufficient  - Selection of participants was not randomized, therefore selection bias should be accounted for  - Lengthy questionnaire | Barriers to effected utilization are multifactorial, mostly relating to perception of the specialty as well as ineffective communication within specialties. With respect to noncancer-related illnesses, major barriers exist regarding consideration for specialty referral.  PC is not universally a recognized subspecialty, and its role in acute hospital care is under-appreciated by medical professionals. | Medium |
|  |  |  |  |  | **Data collection** |  |  |  | **Rigour** |
|  |  |  |  |  | Survey to highlight:  - referral/consultation rate  - conditions for which referral/consultation Is considered  - Condition for referral for non-cancer related terminal illnesses  - Opinions on the relevance of pain and PC in acute care  - Reasons for consult/referral delay  - Barriers to effective referral |  |  |  | Low |

| Author, country | Aim | Research design | Participants | Setting | Intervention | Outcomes | Limitations | Conclusion | Relevance |
| --- | --- | --- | --- | --- | --- | --- | --- | --- | --- |
| Khateeb,  2018,  USA  [27] | To determine the impact of a novel inter-professional intervention with co-rounding PC specialists and hospitalists on PC utilization, time to consultation and to explore whether the intervention would have more impact on noncancer patients. | Pre- post- intervention study from July 1, 2013 through June 30, 2014. | **N = 6.896**  Patients admitted to the 11 hospitalist teams  **Exclusion:**  - Not assigned a team because of overnight admission by a float physician and morning discharge.  - Immediate transfer to another service.  **Hospitalists**  **(n=25)** | **Discipline:**  Wide (all hospitalized patients)  **Hospital size:**  Large  (1000-beds)  **Hospital type:** Quaternary care academic medical center.  **Hospitalist services** **(n=11):**  each staffed by an attending physician. | **Enhancing the scope of discharge rounds** by adding an attending and social worker from the PCT twice weekly.  **During enhanced rounds:** All patients currently on the hospitalist’s service  were discussed.  **Informal recommendations:**  - whether a patient would benefit from formal inpatient consultation.  - symptom management  - goals of care.  In all cases, hospitalists made the final decision whether or not to pursue an inpatient consultation. | **1) Change over time in proportion of admitted patients receiving PC consult:**  - Significant increase after implementation in the intervention group (p=0.0008)  - Multivariate analysis shows no increase attributable to the intervention among cancer patients. It did show PCC increased among noncancer hospitalizations by 1.38%  over the study period for the intervention patients, compared with a 0.38% increased proportion receiving consults among the control patients.  **2) Time to consult**:  - The cumulative proportion of patients receiving a consultation demonstrates the least  time-to-consult in the postintervention study group.  - Controlling for all covariates, time-to-consult to achieve  5% consult was reduced by the intervention in noncancer patients. For cancer patients, the intervention did  not affect the days to consult for intervention  or control teams.  **3) Hospitalists’ survey:**  - 60% RR (15/25)  - All respondents (15/15) reported that the intervention facilitated easier communication with PC consultants  - 83% agreeing that it added value to patient care.  - Only one hospitalist found the intervention to  be too time consuming  - 55% said the intervention improved their own PC skills. | - Possible bias in findings towards no result, because patients never directly receiving the intervention were also included.  - Limited generalizability of findings  **Strength:**  - use of both concurrent and historic controls to capture a difference in change over time. | Scheduled shared inter-professional  rounds between hospitalists and PC specialists increased  utilization and timeliness of PC consultations, particularly among noncancer patients. Regular discussions  between hospitalists and PC providers improved appropriate use of PC services and hospitalist PC skills | High |
|  |  |  |  |  | **Data collection** |  |  |  | **Rigour** |
|  |  |  |  |  | **Intervention group**  **(n=2 hospitalist services)**  - Hospitalists were surveyed to elicit perceived value and/or burden of the interventions and change in utilization of PC services.  **Control group (n=9 hospitalist services):** usual care: PCC was requested ad hoc by hospitalists.  **Change over time in proportion of admitted patients receiving PC consult**: measured as the absolute difference  in percentage of hospitalizations during the intervention  year (2014) minus the prior year (2013)  **Time to consult:** calculated as the time between date of admission to the  date of the first PC consultation. |  |  |  | High |

| Author, country | Aim | Research design | Participants | Setting | Intervention | Outcomes | Limitations | Conclusion | Relevance |
| --- | --- | --- | --- | --- | --- | --- | --- | --- | --- |
| Kyeremanteng et al.,  2019,  Canada  [28] | To determine HCWs perceived need for ICU-PC integration and their perceived quality of EOL care  To identify potential barriers to ICU-PC integration. | Cross-sectional survey study | **N = 203**  - ICU staff physicians (n=16)  - ICU RNs (n=154)  - ICU Fellows (n=5)  - PC staff physicians (n=6)  Other (n=22): Respiratory therapists, ICU administrators, and PC nurses | **Discipline:**  Intensive care  **Hospital size:**  Not reported  **Hospital type:**  - an academic tertiary center with:  TOH-Civic (n=48)  TOH-General (n=85)  - university-affiliated community hospital (Montfort) (n=28)  - University of Ottawa Heart Institute (CSICU) (n=42) | ICU-PC integration  No further information provided because intervention seemed not in place during study period. | - 72,4% reported being comfortable with providing EOL care. However, only 46% rated overall quality of EOL care as good or excellent  - Emotional and organization support for clinicians was generally viewed as suboptimal.  There were clear divisions in opinion regarding whether or not increasing integration of PC will improve quality of EOL care. Overall:  - 78.4% rated increasing ICU-PC integration as beneficial  - 10.3% strongly disagreed with this  A minority of ICU physicians (14%) and ICU nurses (10%) were opposed to ICU-PC integration. On the contrary, al PC physicians were proponents for the integration.  **Potential strategies for integration rated as most feasible:**  - criteria triggered consults  - role modeling in EOL care communication by PCT  **Potential barriers for integration:**  - Lack of understanding of the role of PC  - inadequate communication  - ICU clinicians’ confidence in their own abilities to deliver good PC | - Low RR (45%) and ICU RNs were approx.. 85% of the sample.  - RR of ICY physicians was low likely due to time constraints and the belief that EOL care was already done well.  - Results may be object to observation bias.  - Reporting of participants was done wrong, so how reliable are reported percentages?  - Not clear if there was a PCT/PC specialist already functioning in/with ICU | This study served as a first step toward developing quality improvement intervention. It was able to identify potential barriers toward integration and appropriate strategies to assist the integration of ICU-PC. | Medium |
|  |  |  |  |  | **Data collection** |  |  |  | **Rigour** |
|  |  |  |  |  | 41-item, self-developed questionnaire with open- and closed-ended (likert scale) questions exploring:  - current quality of EOL care in the ICU  - possible benefits if ICU-PC integration  - potential barriers to integration  - appropriate methods to achieve ICU-PC integration |  |  |  | (very) Low |

| Author, country | Aim | Research design | Participants | Setting | Intervention | Outcomes | Limitations | Conclusion | Relevance |
| --- | --- | --- | --- | --- | --- | --- | --- | --- | --- |
| Lehn et al.,  2019,  USA  [29] | To establish a ROI for a pharmacist position on a hospital-based PCT. | Retrospective chart review study from January 2015 through April 2016 | **N = 1660**  Patients with PCC  **In/exclusion:**  Not reported | **Discipline:**  Not reported  **Hospital size:**  Facility 1: Large (634beds)  Facility 2:  Medium (410beds)  **Hospital type:**  Facility 1:  Tertiary referral center/urban teaching hospital  Facility 2:  Not reported | **PC pharmacist integration in PCT:** | - Statistically significant difference in rates of pADEs between PC pharmacists and non-PC pharmacists at facility 1 (p=0.001)  - Statistically significant difference in rates of pADEs between pharmacists at facility 1 compared at facility 2 (p<0.001)  - Contribution of the pharmacist as a physician extender was demonstrated by time savings and effectively extending the reach of the PCC service.  - Total annualized ROI of 1.2 to 2.9million dollars for a PC pharmacist position. | - Retrospective study  - There may be differences in facilities and between non-PC pharmacists that introduced bias.  - Inability to track reduced LOS, prevented ED visits, or avoided hospital 30-day readmissions | This study showed a ROI that  more than justifies a pharmacist’s annual salary and should  validate acquisition of these positions for PCTs nationally | Low |
|  |  |  |  |  | **Data collection** |  |  |  | **Rigour** |
|  |  |  |  |  | Intervention group (n=): patients with PCC from PCT with pharmacist in Facility 1  Control group (n=): patient with PCC from PCT without pharmacist in Facility 2 |  |  |  | Low |

| Author, country | Aim | Research design | Participants | Setting | Intervention | Outcomes | Limitations | Conclusion | Relevance |
| --- | --- | --- | --- | --- | --- | --- | --- | --- | --- |
| Ma et al.,  2019,  USA  [30] | To assess the impact of early triggered palliative care consultation on the outcomes  of high risk ICU patients. | Single-center cluster randomized crossover trial between August 2017 and May 2018. | **N = 199**  Patients admitted to the medical ICU  **Inclusion:**  - 18 y/o  - Positive for at least 1 palliative care criteria  **Exclusion:**  - History of stem cell transplant  - Solid organ transplant within 1 year  - Non-English speaking  - No capacity to participate in PC discussions with no identifiable surrogate  - PCC earlier during the same hospitalization  - Patients already determined to be DNR/DNI. | **Discipline:**  Intensive care  **Hospital size:**  Large  (1250beds)  **Hospital type:**  Not reported  Barnes-Jewish hospital.  ICUs are closed units staffed 24/7 by separate teams of:  - physicians  - nurses  - pharmacists  - respiratory therapists.  The teams are supervised by an intensivists board, certified in critical care, and have traditionally managed PC needs in the ICU including symptom management and discussion regarding goals of care. | **PCC by the PCT within 48 hours of admission**.  PCT consisting of: - Physician board-certified in PC - Nurse practitioners - PC clinical fellow - Social worker - Chaplain  PCC included:  - Chart review - Meeting with patient and available healthcare proxies - Identification of physical and emotional needs of patient and family  - Discussion with primary team on how to best meet those needs  - Communication between all parties with respect to goals, values and treatment decisions | **Primary outcome**  **1) Change in code status:**  - Significantly higher (p<0.0001)  **Secondary outcomes**  **1) Discharge to hospice**  - Significantly more often (p=0.0026)  **2) Duration of mechanical ventilation**  - Significantly shorter (p=0.0415)  **3) Tracheostomy**  - Less performed (p=0.0354)  **4) Post discharge ED visits within 30 days:**  - Fewer presented to the ED after the intervention (p=0.0028)  **5) ICU LoS, Hospital LoS, Hospital mortality, 30-day mortality, and operating costs:**  - No significant differences  **6) Duration of vaso-pressors, cardio-pulmonary resuscitation, hospital readmissions:**  - Not reported | - Study was not  powered for the highly skewed nature of financial data.  - single-center study so results may not be transferrable.  - Potential source of bias in results because of absence of data on potentially eligible patients that were excluded because of staffing limitations.  - QOL or patient/family satisfaction was not measured. | An early directed multifaceted PC intervention led by experienced  clinicians board-certified in PC significantly influenced code status, hospice  referrals, and medical resource utilization. | High |
|  |  |  |  |  | **Data collection** |  |  |  | **Rigour** |
|  |  |  |  |  | **Intervention group (n=97)**  **Control group (n=102):** PCC at the discretion of the medical ICU clinicians.  Process and outcome data was independently collected by two researchers from the EHR |  |  |  | High |

| Author, country | Aim | Research design | Participants | Setting | Intervention | Outcomes | Limitations | Conclusion | Relevance |
| --- | --- | --- | --- | --- | --- | --- | --- | --- | --- |
| McDarby,  2018,  USA  [31] | To identify factors that hinder or facilitate the PCT’s successful collaboration  with other providers from the perspectives of both PCT and nonpalliative specialists. | Semi-structured interview study between July 2017 and July 2018 | **N = 48**  PCT members (n=19)  Non-PCT specialty providers (n=29):  - Nurse practitioner (n=1)  - Physicians (n=28)  **In/exclusion:**  Not reported | **Discipline:**  Not reported  **Hospital size:**  Not reported  **Hospital type:**  - Urban university medical center (n=1)  - Suburban community hospitals (n=3) | **PCC process** | **1) Interactions between providers:**  - Participants reported interactions between PCT and non-PCT providers that were inconsistent and superficial, limiting opportunities for deeper collaboration.  Facilitators  - The majority of interactions were described as more informal, brief, and patient focused.  - key role of visibility of PCT  **2) Patient and family perceptions about PC**  - Providers described patient and family confusion about PC as an impediment to successful collaboration  - A family’s active resistance to the  PCT’s involvement in care could prevent providers from  seeking out a consultation  **3) Provider attitudes toward PC:**  - Negative attitudes and beliefs about PC among non-PCT providers. Some rooted in misinformation and lack of knowledge about the true scope of PC, yet others reflected differences in philosophy about medicine and practice  - Importance of marketing the utility of the PCT/value of demonstrating their relevance.  **4) Education and training about PC**  - misunderstanding of PC as synonymous with hospice care  - education about palliative care and its utility as the best way to correct misconceptions  **5) The role of the PCT**  - discrepancies in descriptions of toles and responsibilities of PCT.  - facilitating communication as key rol of the PCT  **6) PCT’s recommendation implementation:**  - FU strategies to maximize the likelihood of implementation, such as following up “with a phone call, or face to face.” | It could be that providers who chose not to participate in this study had other beliefs, which are not portrayed in this study. | This study highlights barriers to collaboration between PCT  and non-PCT providers, including lack of knowledge,  varying perceptions about the PCTs role, and a  general belief among providers that PCT expertise, while  clearly present, is not always necessary. However, providers  also pointed to strategies that facilitate collaboration.  Meaningful inter-professional teamwork and collaboration is essential, relying on the contributions of all  providers involved. | High |
|  |  |  |  |  | **Data collection** |  |  |  | **Rigour** |
|  |  |  |  |  | **Semi-structured, individual interviews** with questions designed to elicit information about:  - team interactions  - qualities of successful and unsuccessful PCC  - reasons providers might consult PCT  - responsibilities performed by a PCT  - types of recommendations requested from and made by the PCT |  |  |  | High |

| Author, country | Aim | Research design | Participants | Setting | Intervention | Outcomes | Limitations | Conclusion | Relevance |
| --- | --- | --- | --- | --- | --- | --- | --- | --- | --- |
| Mertens,  2020,  Belgium  [32] | To explore healthcare professionals’ experiences regarding the communi-cative aspects of inter-professional collaboration and the involvement of patient and family members. | Phenomeno-logical focus group study between March  and May 2016, | **N = 53**  Healthcare professionals from different care settings and a diverse professional background:  - Physicians (n=10) (GP, oncologist, hematologist, anesthetist)  - Nurses (n=31) (community nurse, specialized PC home nurse, nursing home nurse, nursing assistant nursing home, PC reference nurse, clinical nurse specialist, head nurse)  - Psycho-logists (n=4)  - Social workers (n=4)  - Dieticians or spiritual workers (n=4)  **In/exclusion:**  Not reported | **Discipline:**  Various  **Hospital size:**  Not reported  **Different care settings participated in the study:**  - Hospitals (n=2) all with a PCT and the largest hospital with a PCU of 9 beds.  - Nursing homes (n=2)  - Palliative home care team (n=1)  PC network in Belgium, with focus on one of the 15 PC regions in Flanders. | NA | **1) Information exchange**  Both hospital staff and home care professionals stated that this often did not meet the informational needs. Regarding the method, the absence of a shared EHR to exchange information between professionals within or across care settings was a perceived barrier. Availability of an efficient, digital platform, allowing for information sharing between professionals within and between settings, was deemed essential to overcome barriers of timely, relevant and complete information exchange.  **2) Multidisciplinary team meetings**  Considered valuable to keep all professionals updated on a patient’s case and to discuss shared care goals. However, all participants agreed that the implementation of multi-disciplinary team meetings remained a challenge.  **3) Preparing palliative home care**  NA  **4) Provider-patient communication**  Physicians’ openness about the patients’ condition and prognosis directly affected the organization of appropriate patient care and influenced inter-professional collaboration.  **5) ACP**  In general, participating professionals regarded ACP important for the delivery of high-quality care. | - Choice for homogeneity regarding setting but a heterogeneity regarding professions can maximize the exploration and understanding of setting-specific experiences. By contrast, too much heterogeneity may inhibit discussion. Heterogeneity regarding professions however, may influence the group discussion too due to status difference of the professionals or sensitive topics being discussed and individuals feeling uncomfortable to respond.  - The use of vignettes to initiate the focus group discussion. A limitation could be the distance between the vignette and the social reality. However, we have tried to anticipate this by using concrete patient cases aligned with the care settings of the participants.  - Another limitation may be that participants initially provide socially desirable responses. This was avoided through the use of probing questions. | This study highlighted several areas of improvement on different levels of care and collaboration, such as ineffective  information flow across settings and professionals  due to the absence of a shared EHR,  ineffective inter-professional and provider-patient communication, and a lack of shared care goals. Support from policymakers and researchers is required to  achieve integrated PC in regional networks. | Low |
|  |  |  |  |  | **Data collection** |  |  |  | **Rigour** |
|  |  |  |  |  | Focus group interviews about experiences regarding information exchange, interprofessional communication, and provider-patient communication. |  |  |  | High |

| Author, country | Aim | Research design | Participants | Setting | Intervention | Outcomes | Limitations | Conclusion | Relevance |
| --- | --- | --- | --- | --- | --- | --- | --- | --- | --- |
| Morikawa et al.,  2015  Japan.  [33] | To assess hematologists and PC  specialists’ perception about the roles of the hospital-based PCT and the barriers to collaboration  between hematologists and PCTs on relapse or refractory leukemia and malignant lymphoma patients’ care. | Qualitive interview study from August to December 2011. | **N = 21**  Physicians:  - Hemato-logists (n=11)  - PC specialists (n=10)  **Inclusion:**  - Been working in the area of expertise for a minimum of 5 years | **Discipline**:  Hematology  **Hospital size:**  Not reported  **Hospital type:**  - 1 university hospital  - 1 cancer center  - 1 general hospital | NA | **Perceptions about roles of PCT**  **Perceptions about expectations for PCT**  **Barriers to collaboration**  1) Don’t feel the need to refer  2) Difficulty of timing the referral  3) Lack of aggressive approach by PCT  4) Negative image of PCT  5) Need for specific management in hemato-logical malignancy  6) Lack of communication  7) Others:  - lack of PCT manpower, so hesitation to refer  - Hematologists’ lack of knowledge about PC | - Snowball sampling  - Focus on specific physicians taking care of specific patient population, so not sure if these findings are the same for other populations  - Context is not well defined  - Nothing is said about saturation. Was sample size big enough to have reliable results? | The difference in perception of the HPCT’s  roles is one of barriers to collaboration. Furthermore, based  on hematologists’ and PC specialists’ viewpoints significant barriers to collaboration exist that should be addressed by both the hematology team and the PCT for a better collaboration. | Medium |
|  |  |  |  |  | **Data collection** |  |  |  | **Rigour** |
|  |  |  |  |  | In-depth, face-to-face, semi-structured interviews about the roles and expectations of the PCT, and the barriers to collaboration |  |  |  | High |

| Author, country | Aim | Research design | Participants | Setting | Intervention | Outcomes | Limitations | Conclusion | Relevance |
| --- | --- | --- | --- | --- | --- | --- | --- | --- | --- |
| Nevadunsky et al.,  2013  USA  [34] | To evaluate the association of inpatient palliative medicine consultation with ACE scores and direct inpatient hospital costs of patients with gynecologic malignancies. | Retrospective medical records review study from June 2005 until February 2010 | **N = 100**  Women with gynecologic malignancies  **Inclusion:**  - Treated during the last year at a singly institution  - Died from their primary gynecologic malignancy | **Discipline:** Gynecologic oncology  **Hospital size:**  Large  (1062 beds)  **Hospital type:**  Urban community academic medical center | **Timely PCC**  = formal inpatient PCC 30 days or more from death.  **Criteria for defining PCC:**  - Consultation requestion by attending physician  - patient seen and evaluated by PCT for one or more visits  - at least one set of recommendations was made by PCT for the primary care team. | **Demographics:**  - 49% had inpatient PCC  - 18% had timely inpatient PCC (intervention)  - No differences in referral patterns between junior and senior level gynecologic oncologists  **ACE-scores:**  - Most common aggressive measure at EOL: death in acute care setting for both patients with timely (28%) versus no/untimely (45%) consultation.  - aggressive measures were lower in every category for those with timely intervention except for more than 1 ED visit.  **Hospital costs:**  - The median direct hospital cost for the last 30 days of life for patients with timely consultation was significantly less (p = 0.01). This difference persisted in comparison of median direct costs for the last 14 days of life (p = 0.01). | Cost data of three patients were missing from the hospital accounting database.  Retrospective study, small sample size, single institution  Patients who were not identified by the Tumor Board Registry were not included  Outpatient referrals were not considered.  Savings from inpatient direct hospital costs should be interpreted with caution as there was no accounting for outpatient costs to insurers and costs to the family. | PCC resulted in lower ACE scores when compared to patients who did not receive a timely PCC.  Patients who had timely PCC had decreased interventions in all domains except for ED visits. | Low |
|  |  |  |  |  | **Data collection** |  |  |  | **Rigour** |
|  |  |  |  |  | Consecutive sampling of patients identified from the Gynecologic Oncology Tumor Board Registry.  Data were abstracted from inpatient as well as outpatient medical records for the last year of life. |  |  |  | Low |

| Author, country | Aim | Research design | Participants | Setting | Intervention | Outcomes | Limitations | Conclusion | Relevance |
| --- | --- | --- | --- | --- | --- | --- | --- | --- | --- |
| Nieder et al.,  2016,  Norway  [35] | To address the question: “does early PC in addition to standard oncology care or late additional PC improve patterns of terminal care in patients who died from non-small cell lung cancer?” | Retrospective single-institution study from January 1, 2006 to December 31, 2014. | N = 286  Patients who died from non-small cell lung cancer  **In/exclusion:**  Not reported | **Discipline:**  Pneumo-oncology  **Hospital size:**  Not reported  **Hospital type:**  NHT  all lung cancer care is prescribed and guided by the staff at NHT.  NHT is owned by the Ministry of Health and Care services and administered through a regional trust. | **Early PC provided by PCT:**  - Arbitrarily defined cutoff of 3 months before death | - 8% (22) received early additional PC by PCT  - 65% was not in contact with PCT at all  - patients who received early or late PC were  more likely to be treated with systematic anticancer drugs and with more lines of therapy (p=0.001) and less likely to lack a documented DNR.  - Likelihood of active anticancer treatment in the last month of life was lowest in the early PC group (p=0.03)  - In the early PC group (n=22): 61% had their DNR preference documented earlies than in the last month of life (p=0.0001) and patients in this group were less likely to be hospitalized in the last 3 months of life (p=0.003). | - Proportion of patients who received early PC provided by PCT was much lower than anticipated (8%), which results in limited statistical power.  - Retrospective and single institution.  - Possible selection bias  - Small size of subgroups | Early additional PC provided by PCT resulted in relevant improvements.  The optimal timing of this intervention should be examined prospectively. | Low |
|  |  |  |  |  | **Data collection** |  |  |  | **Rigour** |
|  |  |  |  |  | Medical records available through the patients’ EHR were reviewed retrospectively.  Cohort divided into **3 subgroups:**  **1) early additional PC:** PCT involved > 3months before death  **2) late additional PC:** PCT involved < 3months before death  **3) No additional PC** |  |  |  | Low |

| Author, country | Aim | Research design | Participants | Setting | Intervention | Outcomes | Limitations | Conclusion | Relevance |
| --- | --- | --- | --- | --- | --- | --- | --- | --- | --- |
| Oertel,  2022,  Germany  [36] | To analyze the symptom burden of palliative patients on a radiation oncology ward.  To describe a multi-disciplinary palliative concept and its integration.  To assess educational demands and the acceptance of the proposed model. | Retrospective single-center  cohort study  5 Jan 2015 – 6 Aug 2021 | **N = 1.018**  Patients with advanced, life-limiting and progressive disease  Inpatients (n=941)  Outpatients (n=77)  **In/exclusion:**  Not reported  **N = 15**  Residents in the radiation oncology ward  - filled out an 18-item questionnaire after their obligatory ward rotation.  **In/exclusion:**  Not reported | **Discipline:**  Radiation oncology  **Hospital size:**  Not reported  **Hospital type:**  Not reported | **PCT as consultation service**  Weekly ward rounds by PCT, treating team to identify patients with need for specialized PC.  After referral, a detailed assessment was performed, which resulted in a supportive treatment plan carried out by the PCT, simultaneously to radiation treatment.  Daily interventions could  include a wide spectrum.  The PCT did not interfere directly with decisions on the RO treatment schedule  and duration, but could formulate recommendations | **Pain intensity:**  Of the 192 patients presenting with pain at time of admission, 177 improved (after 72 h). Pain intensity decreased from a median value of 6 on the numeric rating scale (range 2–10) to 2**.**  **Time to integration:** reduced significantly by regular ward rounds (p<0.001)    **Number of patients treated cooperatively:** increased from 97 before implementation to 251 after implementation.  **Number of patients dying on the radiation oncology ward:** decreased significantly (p=0.009)  **Survey**:  -PCT was well known (100%).  -PCT was used often (7.7%) or very often (84.6%) during ward rounds.  -Indications for consultations: pain medication (92.3%), organization of further care (92.3%), and psycho-oncological support (84.6%).  -PCT was seen as: positively, enriching, empathic, collegial, professionally founded and low threshold for consultation.  -Collaboration with PCT results in more extensive and profound knowledge of PC (100%). | Only symptom burden that is described in the results section is ‘pain’, which does not cover everything. So objective one of this study is doubtful.  Follow-up data on mortality  after discharge were incomplete which prevented a decisive survival analysis.  No simultaneous control group, which hampers estimation of the precise impact.  Possible selection bias.  Due to the close cooperation between the two departments, an indirect influence cannot be excluded. | The hereby presented analysis demonstrates feasibility and  efficacy of a structured cooperation between a radiation oncology department and a PCT. | High |
|  |  |  |  |  | **Data collection** |  |  |  | **Rigour** |
|  |  |  |  |  | PC needs were identified by the ward physician, if necessary patients were referred to PCT.  18-item questionnaire filled out by residents in radiation oncology after their obligatory ward rotation aimed at palliative knowledge as well as use of and views on PCC service |  |  |  | Low |

| Author, country | Aim | Research design | Participants | Setting | Intervention | Outcomes | Limitations | Conclusion | Relevance |
| --- | --- | --- | --- | --- | --- | --- | --- | --- | --- |
| Pan et al.,  2017,  Taiwan  [37] | To elucidate the effect of path modeling on the knowledge, attitude and practice toward PCC in  Taiwanese nursing staff. | Cross-sectional descriptive study  2013 | **N = 284**  Nurses  **Inclusion:**  - RNs  - working in the ward or ICU  - able to communicate in Mandarin  - willing and able to participate in the study | **Discipline:**  Wide (see Table1)  **Hospital size:**  Not reported  **Hospital type:** Medical Center | Not applicable | **1) Significant predictors of Knowledge:**  - ward (hematology and oncology)  - participation in education related to PC  - frequency of contact with PCC service  **2) Significant predictors of Attitude:**  - ward (hematology and oncology)  - experience of death  - frequency of contact with PCC service  **3) Significant predictors for Practice:**  - participation in education related to PC  - frequency of contact with PCC service  **4) Path modelling:**  - having a master’s degree significantly affected practice through knowledge and attitude compared to colleagues with an undergraduate degree (p=0.004)  - educational level, marital status, ward, and the frequency of contact with PCC service directly affected practice. | - Cross sectional design, unicentric, only nurses. However, relatively big sample size  - Taiwanese nursing staff may be reluctant to report their true opinions due to cultural differences, which might lead to over-reporting. | We suggest nurses should have access to a PCC service team for consultation and the opportunity to discuss patients with requiring PC. This may allow nurses to appreciate the importance of PC, improve palliative nursing practices, and enhance the quality of care for patients receiving EOL care. | Medium |
|  |  |  |  |  | **Data collection** |  |  |  | **Rigour** |
|  |  |  |  |  | **Questionnaire**  KAP-PCCSI composed of 3 scales measuring:  - Knowledge  - Attitude  - Practice  regarding PCC service.  All items rated on a 1-5 score  Path modelling for examining causal patterns among variables |  |  |  | High |

| Author, country | Aim | Research design | Participants | Setting | Intervention | Outcomes | Limitations | Conclusion | Relevance |
| --- | --- | --- | --- | --- | --- | --- | --- | --- | --- |
| Rocque et al.,  2015,  USA  [38] | To evaluate feasibility and to identify the impact of implementing triggered PCC as part of standard care  for patients admitted to the solid-tumor oncology service with advanced cancer | **Prospective, pre-post , sequential, three-cohort study**  1 Apr 2012 - 15 Jul 2013 | **N = 203**  Patients admitted to the solid tumor oncology service with advanced cancer    **Inclusion:**  - Unplanned admission | **Discipline:**  Solid tumor oncology  **Hospital size:**  Not reported  **Hospital type:**  Academic medical center | **Triggered PCC**  - focus on illness understanding, prognostic awareness, physical symptoms and GoC  - written summary provided to primary outpatient oncologist  **Cohort 1 (n=65):**  pre-implementation patients  **Cohort 2 (n=70):** implementation patients  **After analysis of Cohorts 1 and 2, barriers to implementation were identified and intervention was modified:**  - 1 physician responsible for the triggered PCC and the inpatient unit + attending multi-disciplinary rounds  - second physician staffed non oncology consults.  **Cohort 3 (n=68):**  Adjusted implementation | **PCC:**  - 16% in cohort 1  - 60% in cohort 2  - 62% in cohort 3  **Patient outcomes:**  - Disease understanding was improved, but there was no change in health-related QOL, symptom burden or satisfaction (p>0.05)  **Resource utilization and survival:**  - No differences in ACP or hospice use  - No differences in enrollment on hospice for > 3weeks  - Resource utilization was similar between cohorts  - No significant difference in overall survival between cohorts (p=0.32)  **Physician perspectives:**  - For discharges in which patients had a triggered PCC, the oncologist felt that patient care was enhanced in 62% of cases and indicated that, in 24% of cases, the management was altered as a result of the PC consult.  - Improvement in the physician satisfaction with the discharge outcome after triggered PCC initiation (P=0.03).  - 9 out of 34 times the oncologist felt that the triggered PCC saved time | - Single institution  - Patients with language barriers: included, but not provided with survey.  - To improve survey completion rates, patients were allowed to complete survey at any time, including before or after the triggered PCC for cohorts 2 and 3  - 26 missed patients in cohort 3  - patient survey data limited due to low completion rates  **Strengths:**  - waiting period before implementing triggered consults to minimize the number of patients in Cohort 1 who were readmitted during collection of Cohort 2 data.  - Longitudinal, pre-post  - Takes into account many outcomes  - Sample size quite big for single institution and specific population. | Triggered PCC significantly improved patients’ prognostic awareness, oncologists’ satisfaction, and patient care generally improved.  Triggered PCC had minimal measurable impact on patient reported outcomes and service utilization | High |
|  |  |  |  |  | **Data collection** |  |  |  | **Rigour** |
|  |  |  |  |  | **Feasibility:**  - monitoring of patients who were eligible but did not receive the intervention.  - In Cohort 3 also reasons were monitored.  **Patient reported outcomes**:  - baseline surveys  - FU surveys at 2 weeks and 2 months after discharge  **Resource utilization and survival:**  - data from EHR  **Physician perspectives:**  - survey about impact of the intervention |  |  |  | High |

| Author, country | Aim | Research design | Participants | Setting | Intervention | Outcomes | Limitations | Conclusion | Relevance |
| --- | --- | --- | --- | --- | --- | --- | --- | --- | --- |
| Sarradon-Eck et al.,  2019,  France  [39] | To describe French oncologists’ perceptions of early PC and their effects on referral practices before a clinical early PC trial was launched. | **Qualitative, semi-structured, interview study**  Oct 2015 - May 2017 | **N = 32**  - Oncologists (n=13)  - PC specialists (n=19)  **In/exclusion:**  Not reported | **Discipline:**  Oncology  **Hospital size:**  Not reported  **Hospital type:**  University hospital  - PC specialists (n=4)  Comprehensive Cancer Center:  - Oncologists (n=13  - PC specialists (n=15 | **Early PC** | **Avoidance of the word ‘PC’:**  - All oncologists said they would be more likely to refer patients for PC at an earlier stage if it was renamed ‘supportive care’  **Making a change of name:**  - because of stigmatization of PC  **Effects on referral practices:**  - Oncologists’ feared that announcing referral to PC might have negative effects on patients, this could be why oncologists are reluctant to refer.  **Early PC - a poorly understood concept:**  - Some PC specialists think the value of PC is not properly recognized  - PC specialists are regarded as service providers rather than delivering care as part of integrated care  - PC specialists find it difficult to work together with practitioners, since the specialization is not unanimously recognized at French hospitals | - Single institution, generaliz-ability of findings?  - Oncologists who were totally opposed to referring their patients were not interviewed  **Strengths:**  - To assure robustness of analysis, strategies used: saturation, reflexivity, peer review. | Findings bring to light the fact that referral to PC service early after the diagnosis of advanced cancer increases the terminological barriers, induces avoidance patterns, and makes early disclosure of poor prognosis harder for oncologists. The main reason for this situation is the restrictive picture of PC as terminal care. | Medium |
|  |  |  |  |  | **Data collection** |  |  |  | **Rigour** |
|  |  |  |  |  | **Semi-structured, individual, face-to-face interviews.**  **Sociological approach** was used to unravel social and cultural factors underlying oncologists’ attitudes to early referral and working together with PC specialists.  **Semi-structured interview guide covered areas of physicians perceptions** of PC, communicating prognoses, current referral practices, perceived barriers to patients’ access to early PC. |  |  |  | High |

| Author, country | Aim | Research design | Participants | Setting | Intervention | Outcomes | Limitations | Conclusion | Relevance |
| --- | --- | --- | --- | --- | --- | --- | --- | --- | --- |
| Sasahara et al.,  2014,  Japan  [40] | To investigate, through the use of a standard format, the activities performed by hospital PCTs in Japan | Prospective, multicentric, observational study  Aug 2010 - Oct 2011 | **N = 21**  Hospital PCTs  **Inclusion:**  - hospital-based PCTs  - performing PCC  - able to register at least 50 consecutive inpatients referred to PCT. | **Discipline:**  Wide  **Hospital size:**  **Median:** Large (621beds)  **Range:** Medium (327beds) – Large (1182beds )  **Hospital type:**  16 out of 21 hospitals were designated cancer hospitals, other 5 not specified. | NA | **Hospital and patient backgrounds:**  Not relevant  **Reasons for referral and problems identified by PCTs:**  Not relevant  **Recommendations by PCTs:**  - 74% pharmacological treatment  - 49% care for patients’ physical symptoms  - 38% support for patients’ decision-making  **Activities performed by PCTs:**  - 90% Comprehensive assessment  - 77% care for patients’ physical symptoms  - 74% Pharmacological treatment  - 28% Staff support, such as information, education, and emotional support. Although little is known about the type of staff support PCTs provide, data that are available show a positive effect. So, staff support is an important role for PCTs | - Components of PCT’s activities were quantitatively determined, which gives limited insights in CMOs  - Only descriptive, no control, no outcome measures  - Only mature teams. Percentages of activities performed in newly established PCTs might be different.  - Form might be limited in relevance and comprehensiveness  - Data on all activities might not be completely collected. | The results of this study and the format for reporting PCT activity could be effective for improving PCT practice and for the education of new hospital PCT members. | Low |
|  |  |  |  |  | **Data collection** |  |  |  | **Rigour** |
|  |  |  |  |  | PCTs were asked to recruit 50 consecutive referred patients and to fill out a standard form for reporting its activities for each patient.  **This form included:**  - Cover sheet  - Reasons for referral/problems identified by PCT  - Activities  **Patients recruited (n = 1055):** |  |  |  | Low |

| Author, country | Aim | Research design | Participants | Setting | Intervention | Outcomes | Limitations | Conclusion | Relevance |
| --- | --- | --- | --- | --- | --- | --- | --- | --- | --- |
| Selvaggi,  2014,  USA  [41] | To report on a quality improvement program in which a PC service was created and PC providers were embedded into a hematological malignance unit where no PC program previously existed | A pilot feasibility study  Aug 2006 - May 2010 | **N = 256**  unique  patients with hematological malignancies.  **In/exclusion:**  Not reported  **N = 18**  HM-BMT physicians  **In/exclusion:**  Not reported | **Discipline:**  Hematology  **Hospital Size:**  Not reported  **Hospital type:** The Western Pennsylvania Hospital in Pittsburg  **HM-BMT unit staff:**  - 20 inpatient beds  - >500 admissions/  year  - 6 attending physicians  - 12 fellows  - 30 nurses  - 1 care coordinator  - 1 social worker  - 2 transplant coordinators. | **PC program**  **1. Didactic education:** sessions given by PC team. 10 sessions  Duration: 1h/session.  **2**. **Clinical consultation and education:**  PC team:  - joined on rounds  - had discussions with the primary physician  - attended weekly inter- disciplinary meetings  - made recommendations and communicated these to the assigned professional.  - one member was present 2h every day for informal discussions and face-to-face communication. | **Total amount of consultations = 392** (in 256 unique patients.)  **Reasons for referral for all consults (392)**  - 71% (278) pain control  - 44% (172/392) goals of care discussions  **Pain level acceptability in consults for pain control (278)**  - 70% (194) had (very) unacceptable pain at baseline  - 66% (129) had (very) acceptable pain levels within 48hours of consultation.  **Goals of care discussion**  - 41% (104) of the unique patients (256) had DNR/DNI order entered over the course of the program.  **Satisfaction survey**  RR: 78% (n=14)  - 100% were ‘extremely satisfied’ with overall experience with PC service and found PC service “extremely helpful”  - 60% identified management of delirium, depression, and anxiety as needing improvement | Limited data was collected to assess the success of the program.  No financial data was collected.  Leader of this initiative had expertise both in hematological malignancies and palliative care | The PC consultation service led to - an increase of the number of hospice referrals  - pain control for many patients  - increased numbers of documented goals of care discussions  The PC program was well received by the HM-BMT physicians especially in the areas of commu-nication  skills, psychosocial support, and EOL care discussions. | High |
|  |  |  |  |  | **Data collection** |  |  |  | **Rigour** |
|  |  |  |  |  | **1) Needs assessment** through semi-structured interviews with unit staff  **2) Satisfaction survey** among HM-BMT physicians 18months after program initiation for:  **acceptability, usefulness**  **effectiveness** of the program |  |  |  | High |

| Author, country | Aim | Research design | Participants | Setting | Intervention | Outcomes | Limitations | Conclusion | Relevance |
| --- | --- | --- | --- | --- | --- | --- | --- | --- | --- |
| Spalding et al.,  2016,  USA  [42] | To examine whether the diction and phrasing of consultation recommend-ations in the EHR  influence their implement-ation. | Retrospective EHR review study  1 Jan 2009 – 31 Dec 2010 | **N = 198**  Patients with PCC  **Inclusion:**  - recommend-ation suggested an action be taken by primary care team  **Exclusion:**  - Consultation requests from psychiatry and hospice  - PCT never having the opportunity to see the patients, because the patient was discharged, transferred, or death. | **Discipline:**  Not reported  **Hospital size:**  Not reported  **Hospital type:**  Urban Veterans Affairs Medical Center | **Consultation recommendations** by PCT in EHR.  - PCT consisting of a physician, nurse practitioner, psychologist, social worker, and part-time chaplain.  - recommendations of first visit | - Presence of certain words within the consultation recommendation was associated with the likelihood that recommendations were implemented.  - When recommendations included any **conditional words**, they were **significantly less likely to be implemented** (p=0.005)  - Several words had a **positive impact** on recommendation implementation, namely: ‘encourage’, ‘recommend’, ‘would’, and ‘should’. However, none were statistically significant. Most likely due to the relatively low frequency of their use. | - PCT was consulted on 657 unique patients, a random sample of 200 was chosen for analyses. No control patients.  - Single center, small sample  - Small effect size  - Results do not provide insight into how providers interpret recommend-ations that include different type of phrasing. | This study demonstrates that the words PCTs use to convey their recommend-ations influence the likelihood of their implement-ation. | Low |
|  |  |  |  |  | **Data collection** |  |  |  | **Rigour** |
|  |  |  |  |  | EHR review until patient was discharged or died to determine whether each recommendation had been implemented.  Details of patient treatment and care guidelines were read to observe whether they aligned with the recommendations from PCT consultations.  **N = 288 PCT consultation recommendations** |  |  |  | Low |

| Author, country | Aim | Research design | Participants | Setting | Intervention | Outcomes | Limitations | Conclusion | Relevance |
| --- | --- | --- | --- | --- | --- | --- | --- | --- | --- |
| Van der stap et al.,  2022,  The Netherlands  [43] | To identify the barriers and facilitators to multidimensional symptom management and potential solutions to improve clinical practice by exploring stakeholders’ experiences. | Qualitative research with focus groups  2019 | **N=51**  6 focus groups:  - 6 patient reps  - 12 community nurses  - 8 hospital nurses  - 8 general practitioners  - 9 hospital physicians  - 8 PC specialists  **Inclusion:**  Broad range of stakeholders who are involved in palliative care symptom management | **Discipline:**  Wide  **Hospital size:**  Not reported  **Hospital type:**  Academic hospitals | NA | **Barriers:**  **1. Multidimensional symptom assessment**  **2. Initiating management of nonphysical problems**  - Lack of PC affinity and skills among generalist psychologists  - Lack of systematic referrals to psychosocial clinicians like psychologists  **3.** **Multidisciplinary collaboration:**  - Hierarchical difficulties in the role division between generalist physicians and generalist nurses/PC specialists  **4. Health-care organization**  - Lack of time to assess and manage all symptoms and problems during the standard time frame for patient consultations  - Lack of reimbursement for referrals to psychosocial or spiritual caregivers  **Facilitators:**  **3. Multidisciplinary collaboration:**  Generalist nurses and physicians collaborating with PC specialists. | Purposive and convenience sampling (nonresponse bias) | Symptom management can improve by helping clinicians improve their communication skills. Generalist clinicians should be encouraged to use systematic approaches to help identify physical symptoms and nonphysical problems that would otherwise be overlooked. | Medium |
|  |  |  |  |  | **Data collection** |  |  |  | **Rigour** |
|  |  |  |  |  | Invitations to participate were distributed nationwide.  **Topic guide:**  - What does symptom management evoke, what little attention, how do you assess, how do you initiate intervention and monitor symptoms, attention for all 4 dimensions?  - How do different disciplines approach symptom management  - Which situations are difficult  - What do you need to cope better  - If you were in charge, what would symptom management look like  Focus groups lasted +/- 2h  **Thematic analysis with an inductive approach** |  |  |  | High |

| Author, country | Aim | Research design | Participants | Setting | Intervention | Outcomes | Limitations | Conclusion | Relevance |
| --- | --- | --- | --- | --- | --- | --- | --- | --- | --- |
| van der Stap et al.,  2021,  The Nether-lands  [44] | To assess the self-perceived barriers, educational needs and awareness of available palliative  care support options among our hospital primary care teams.  To evaluate PC referral patterns. | Single-center mixed methods study  Jan 2012 - Dec 2017 | **Part A**  **N = 486**  - Baseline:  n = 291  - Follow-up:  n = 195  Nurses and physicians  **In/exclusion:**  Not reported  **Part B**  **N = 1404**  Patients referred by clinicians to PCT  **In/exclusion:**  Not reported  **Part C**  **N = 434**  Patients for which referring physicians predicted survival  **In/exclusion:**  Not reported | **Discipline:**  Wide  **Hospital size:**  Not reported  **Hospital type:**  University Medical Center | **PCT implementation** *(initiated in 2012)*  Inpatients can be referred by physicians and nurses of primary care teams, and by self-referral.  Weekly multi-disciplinary team meetings. | **Part A**  Open-ended question 1: **Self-perceived barriers to PC:**  1. Late initiation (25% in 2012 and 20% in 2016):  - Disease-directed treatment is continued too long  - Patients are often identified too late  2. Logistical issues (19% in 2012):  - Lack of time  - Insufficient resources  3. Insufficient general PC knowledge (14% in 2016)  Open-ended question 2: **Self-perceived educational needs:**  - management of physical symptoms (30% in 2012; 21% in 2016).  - basic PC principles ((20% in 2012; 19% in 2016)  Multiple choice questions on **awareness of PC support options:**  - Familiarity with PCT increased from 56% to 85% (p<0.001)  - In 2012, 8% of respondents appraised their experiences with the PCT as excellent and 71% as good, and this increased to 40% and 49% of respondents in 2016 (p<0.001).  **Part B**  1. PC referrals :  - increased by a mean of 28% per year  - Patients were referred most frequently by clinicians working in medical oncology, radiation oncology, pulmonology, and general surgery  2. Timing of referral:  - 26% early (OS after referral ≥3 months)  - 37% late (OS after referral ≥2 weeks and <3months)  - 37% in the dying phase (OS after referral <2 weeks)  - proportion of early referrals increased over time only in referrals made by medical oncology clinicians (P=0.016)  **Part C**  - Survival was correctly predicted in 50% of patients and overestimated in 44%. | - Single center  - RR low in survey study with differing sample size at baseline and FU  - PCT members analyzed quality survey data on their own performance, which may be a source of bias.  - Clinicians who filled out the survey may have had a higher affinity to PC than non-responders which may have affected the survey results.  - Registration bias may be present due to retrospective nature of referral analysis in part B. | Despite increased awareness and use of available PC  support options, self-perceived barriers and educational  needs of primary care team clinicians persisted after 5 years  of clinical and nonclinical PCT activities.  Primary care  teams usually referred late for specialist PC and tended to overestimate survival at referral. Therefore, we recommend that PCTs focus their  nonclinical activities on improving general PC  knowledge among primary care team clinicians. | High |
|  |  |  |  |  | **Data collection** |  |  |  | **Rigour** |
|  |  |  |  |  | **Part A:** **baseline (2012) and follow-up (2016) survey** on barriers,  educational needs and awareness of PC  support options:  - 8 open-ended questions  - 26 multiple choice questions  ! This study reports on 2 open-ended and 6 multiple choice questions  ! Open-ended question:  - RR at baseline: 46% (134/291)  - RR at FU: 56% (110/195)  ! Open-ended question 2:  - RR at baseline: 21% (61/291)  - RR at FU: 37%  (72/195)  **Part B: cohort study**  Data on all patients referred to the PCT from January 2012 to December 2017 were retrospectively collected.  **Part C: clinical prediction of survival at referral**  From June 2014 onwards, all physicians were asked at referral if they would be surprised if their patient would die  within 1 year, 3 months or 2 weeks to assess their ability to prognosticate. |  |  |  | High |

| Author, country | Aim | Research design | Participants | Setting | Intervention | Outcomes | Limitations | Conclusion | Relevance |
| --- | --- | --- | --- | --- | --- | --- | --- | --- | --- |
| Wyshamet al., 2017, USA  [45] | To explore clinician attitudes and beliefs about ICU-based palliative care delivery | Cross sectional study  May - Nov 2015 | **N = 303**  - 150 nurses  - 114 intensivist physicians  - 39 advanced practice providers (APPs)  **Inclusion:**  - Nurses, physicians and APPs from closed model adult medical and surgical ICU’s  **Exclusion:**  - Resident or intern physician training level | **Discipline:**  Intensive care  **Hospital size:**  Large (not specified)  **Hospital type:**  Academic hospitals:  - private urban hospital  - public urban hospital  - private non-profit hospital | NA | **Preferences for integrating PC:**  - 75% felt that PCC was underutilized  - 63% believed that protocolized PCC was effective;  - 73% reported high interest in developing novel systems of PC  - nurses were more likely to characterize PC as underutilized  **Beliefs about criteria for eligibility**  - 68% of nurses and 77% of physicians felt that a PC needs assessment should be a component of a candidate PC system  - 95% reported screening should be based on PC service itself and not only on order of ICU physician  **Factors to enhance clinician interest:**  - 67% felt ICU team should approve trigger lists before implementation  - nurses more frequently agreed that they should be able to initiate referral  **Acceptability of screening trigger:**  - Most favored triggers: family needs; conflict; metastatic malignancy; unrealistic GoC; and help needed with GoC discussions  Respondents described conflict about provider roles in future collaborative PC systems. | - Cohort does not include less resourced and smaller ICUs    - No primary physicians were included such as oncologists or cardiologists | ICU clinicians support the development of protocolized, collaborative palliative care systems and are willing to give up substantial autonomy to promote them. Respondents reported disagreement about the role of ICU nurses in these systems. | Low |
|  |  |  |  |  | **Data collection** |  |  |  | **Rigour** |
|  |  |  |  |  | **Survey** designed by authors to assess:  - clinicians attitudes how to integrate PC specialists - preferences for eligibility screening  - factors that would enhance acceptability  - agreement regarding 23 PC trigger criteria |  |  |  | Low |

| Author, country | Aim | Research design | Participants | Setting | Intervention | Outcomes | Limitations | Conclusion | Relevance |
| --- | --- | --- | --- | --- | --- | --- | --- | --- | --- |
| Yang et al.,  2018  Singapore  [46] | To explore the views and experience of  oncology and palliative care professionals on the co-rounding model compared to an inpatient consult service. | Pilot study with 1-on-1 in-depth interviews  1 Mar - 31 May 2016 | **N = 11**  Oncology and palliative healthcare professionals  - 8 physicians  - 3 nurses  Specialty:  - 9 medical oncology  - 2 palliative medicine  **In/exclusion:**  Not reported | **Discipline:** Medical oncology and palliative medicine  **Hospital size:** Large  (1.597 beds)  **Hospital type:** Singapore General Hospital with > 78.000 admissions/ year, of which about 5.000 under the Department of Medical Oncology | **SPARK co-rounding model:**  Specialist PC physician + APN + internal medicine resident joined the oncology rounding team to provide PC alongside the oncology doctors and nurses as integrated members. | Experiences and views on the SPARK co-rounding model of care in contrast with usual care:  **1) Efficiency of care delivery:**  - access to PC input  - team communications  - parallel workflow  **2) Quality of patient care**  - holistic approach to cancer care  - rapport building with patients and their families | - Limited sample, however interviews were conducted until data saturation.  - Resource implications of this alternative co-rounding model are unclear.  - Wider impact on PC manpower and resources is not addressed here.  - Small scale pilot study, limiting general-izability  - Voluntary nature of participation might have introduced selection bias. | the co-rounding model  improved efficiency of PC delivery as a result of  accessibility to PC expertise, effective team communications, and parallel workflow.  It increased the visibility of the PC team in  oncology care, rendering a holistic approach to cancer care and earlier opportunities to build rapport between the PCT  and patients and their families. | High |
|  |  |  |  |  | **Data collection** |  |  |  | **Rigour** |
|  |  |  |  |  | **1-to-1 in-depth interviews**  Interview guide with open-ended questions on participants’ experience of and views on the SPARK co-rounding model of care in contrast with usual care. |  |  |  | High |

| Author, country | Aim | Research design | Participants | Setting | Intervention | Outcomes | Limitations | Conclusion | Relevance |
| --- | --- | --- | --- | --- | --- | --- | --- | --- | --- |
| Zemplenyi et al., 2021, Hungary  [47] | To evaluate the healthcare costs of palliative care consult service to usual care | Retrospective cohort study  1 Jan 2014 - 31 Dec 2016 | **N = 1,516** (total)  Patients with metastatic cancer, admitted to the clinical centre.  **N = 197**  **(**intervention)  **N= 1319** (control)  **Inclusion:**  - All hospital admissions of cancer patients during the study period and under the care of oncology clusters.  **Exclusion:**  - Admissions with hospital LoS > 60 days  - Admissions across different time periods  - Admissions with change in cluster | **Discipline:**  Oncology  **Hospital size:**  400 beds, not in paper (found online)  **Hospital type:**  University hospital | **PCC service program:** established to provide PC to hospitalized patients with complex needs and to coordinate integrated care across providers.  **PCC:**  - Symptom control  - Psychosocial support  - patient pathway management across PC providers. | **No statistical differences observed in costs** across categories for the overall matched groups.  “**Early initiation**” of PC is associated with significant reduction in inpatient costs (difference 493€, p=0.013).  The **proportion of patients who died in the hospital was lower in the PCC service** group compared to the usual care group (66% vs. 85%, p = 0.022) | - Retrospective approach used to determine prognosis by estimating number of days prior to death - Informal care costs not included   Control group | Early palliative care of hospitalized patients is associated with lower costs for the healthcare system. | Low |
|  |  |  |  |  | **Data collection** |  |  |  | **Rigour** |
|  |  |  |  |  | **Control group**  - Routine hospital care  - Regular patient pathway management across providers   - **Matching** 197 pairs of PC group with usual care group   **Healthcare costs:**  Estimated from payer’s perspective, calculated as total expenditure of National Health Insurance Fund  **Patient level data:** electronic medical records |  |  |  | Low |

| Author, country | Aim | Research design | Participants | Setting | Intervention | Outcomes | Limitations | Conclusion | Relevance |
| --- | --- | --- | --- | --- | --- | --- | --- | --- | --- |
| Zemplenyi et al.,  2020, Hungary  [48] | To give a **comprehensive overview and gain insights on the PCC service programme** covering the six components of the conceptual framework for integrated care and to share the **barriers to the implementation** alongside strategies that were applied to overcome them. | **Qualitative analysis** of the PCCS programme, based on **care provider documents** and **interviews** with stakeholders | **N=15**  30-90 min per interview  Different stakeholders:   - Managers (n=4) - Physicians (n=3) - Non-physicians (n=4: 1 hospice nurse, PC psychologist ; 2 head nurses) - Informal caregivers (n=2)   Patients (n=2)  **In/exclusion:**  Not reported | **Discipline:**  Oncology  **Hospital size:**  400 beds, not in paper (found online)  **Hospital type:**  University hospital | **PCC service:**  Patient care is provided by a dedicated team working closely with other hospital professionals. The task of the team is to respond quickly to the needs of the patients palliative care coordinator is available 5 days a week for personal consultations (as requested by doctors from different departments) at any clinical departments and answers a hotline on the weekends | **Structure** of PCC service:  - Requesting consultation 🡪 Introductory assessment 🡪 Creating a treatment plan 🡪 Organizing transfer to other provider 🡪FU.  **Service delivery**  **Barriers:**  - Acceptance of the palliative care principles in the clinical departments  - Aligning the PC activities to working procedures of the department  **Facilitators:**  - PCC service team manager approached the heads of the clinical department  - Regular consultation and feedback from care providers, patients and family members  - Members of the PCT participate in the medical and nursing education and training  **Workforce**  **Barriers:**  - A high risk was identified (burnout, low income, lack of recognition) in terms of employee retention  - General shortage of physicians and nurses in Hungary  **Facilitators:**  - PCT members are very committed  - Close cooperation between providers is facilitated by the overlapping of human resources (members of the team work in parallel for more providers). | Limited number of personal views and experiences  No quantitative effect outcome | Relevant health policy regulations and financial schemes to support palliative care consultation services are still missing  Implementation of integrated care service delivery is feasible on institutional levels but the sustainability and enhancement of such programmes require a more systematic approach | Medium |
|  |  |  |  |  | **Data collection** |  |  |  | **Rigour** |
|  |  |  |  |  | Duration of 30-90 min per interview |  |  |  | Low |

# References

1. Alsirafy SA, Abou-Alia AM, Ghanem HM. Palliative care consultation versus palliative care unit: which is associated with shorter terminal hospitalization length of stay among patients with cancer? The American journal of hospice & palliative care. 2015;32(3):275-9.

2. Amano K, Morita T, Tatara R, Katayama H, Aiki S, Kitada N, et al. Assessment of Intervention by a Palliative Care Team Working in a Japanese General Hospital. American Journal of Hospice & Palliative Medicine. 2015;32(6):600-3.

3. Anandan S, Reyes A, Izard S, Magalee CJ, Lopez S. A Retrospective Study Analyzing a Palliative Care-Hospital Medicine Collaboration to Improve Quality of Care of Patients With Advanced Illness. The American journal of hospice & palliative care. 2023;40(3):299-310.

4. Artioli G, Bedini G, Bertocchi E, Ghirotto L, Cavuto S, Costantini M, et al. Palliative care training addressed to hospital healthcare professionals by palliative care specialists: a mixed-method evaluation. BMC Palliative Care. 2019;18(1):N.PAG-N.PAG.

5. Atayee RS, Sam AM, Edmonds KP. Patterns of Palliative Care Pharmacist Interventions and Outcomes as Part of Inpatient Palliative Care Consult Service. Journal of Palliative Medicine. 2018;21(12):1761-7.

6. Autor SH, Storey SL, Ziemba-Davis M. Knowledge of Palliative Care. Journal of Hospice & Palliative Nursing. 2013;15(5):307-15.

7. Barratt SL, Morales M, Spiers T, Al Jboor K, Lamb H, Mulholland S, et al. Specialist palliative care, psychology, interstitial lung disease (ILD) multidisciplinary team meeting: A novel model to address palliative care needs. BMJ Open Respiratory Research. 2018;5(1).

8. Beck KR, Pantilat SZ, O'Riordan DL, Peters MG. Use of Palliative Care Consultation for Patients with End-Stage Liver Disease: Survey of Liver Transplant Service Providers. Journal of Palliative Medicine. 2016;19(8):836-41.

9. Berglund K, Chai E, Moreno J, Reyna M, Gelfman LP. A Social Worker-Led Primary Palliative Care Model for Hospitalized Patients Admitted to the Hospital Medicine Service. Palliat Med Rep. 2020;1(1):234-41.

10. Böling S, Berlin JM, Berglund H, Öhlén J. No ordinary consultation - a qualitative inquiry of hospital palliative care consultation services. Journal of health organization and management. 2020.

11. Braus N, Campbell TC, Kwekkeboom KL, Ferguson S, Harvey C, Krupp AE, et al. Prospective study of a proactive palliative care rounding intervention in a medical ICU. Intensive Care Med. 2016;42(1):54-62.

12. Cannon ST, Gabbard J, Walsh RC, Statler TM, Browne JD, Marterre B. Concordant palliative care delivery in advanced head and neck cancer. American Journal of Otolaryngology - Head and Neck Medicine and Surgery. 2023;44(1).

13. Connolly M, Ryder M, Frazer K, Furlong E, Escribano TP, Larkin P, et al. Evaluating the specialist palliative care clinical nurse specialist role in an acute hospital setting: a mixed methods sequential explanatory study. BMC Palliative Care. 2021;20(1).

14. Courtright KR, Srinivasan TL, Madden VL, Karlawish J, Szymanski S, Hill SH, et al. “I Don't Have Time to Sit and Talk with Them”: Hospitalists’ Perspectives on Palliative Care Consultation for Patients with Dementia. Journal of the American Geriatrics Society. 2020;68(10):2365-72.

15. Coym A, Oechsle K, Kanitz A, Puls N, Blum D, Bokemeyer C, et al. Impact, challenges and limits of inpatient palliative care consultations - perspectives of requesting and conducting physicians. BMC health services research. 2020;20(1):86.

16. De Meritens AB, Margolis B, Blinderman C, Prigerson HG, Maciejewski PK, Shen MJ, et al. Practice patterns, attitudes, and barriers to palliative care consultation by gynecologic oncologists. Journal of Oncology Practice. 2017;13(9):e703-e11.

17. Economos G, Bonneville-Levard A, Djebari I, Van Thuynes K, Tricou C, Perceau-Chambard E, et al. Palliative care from the perspective of cancer physicians: a qualitative semistructured interviews study. Bmj Supportive & Palliative Care. 2023;13(1):95-101.

18. Firn J, Preston N, Walshe C. Ward social workers' views of what facilitates or hinders collaboration with specialist palliative care team social workers: A grounded theory. BMC Palliative Care. 2017;17(1).

19. Friedrichsen M, Hajradinovic Y, Jakobsson M, Brachfeld K, Milberg A. Cultures that collide: an ethnographic study of the introduction of a palliative care consultation team on acute wards. BMC Palliative Care. 2021;20(1).

20. Gatta B, Turnbull J. Providing Palliative Care in the Medical ICU: A Qualitative Study of MICU Physicians' Beliefs and Practices. American Journal of Hospice & Palliative Medicine. 2018;35(10):1309-13.

21. Hill RR, Willett EC, Manuel JD, Delate T. Impact of Palliative Care Clinical Pharmacists in an Inpatient Care Setting on Total Health Care Expenditures. Journal of Palliative Medicine. 2022;25(10):1518-23.

22. Huang LC, Tung HJ, Lin PC. Associations among knowledge, attitudes, and practices toward palliative care consultation service in healthcare staffs: A cross-sectional study. PLoS ONE. 2019;14(10).

23. Jackson White S, Minick P. The Perceptions of Non--Palliative Care Hospitalist Physicians Referring Patients to a Hospital Palliative Care Program. Journal of Hospice & Palliative Nursing. 2016;18(1):39-45.

24. Jacobsen J, Alexander Cole C, Daubman BR, Banerji D, Greer JA, O'Brien K, et al. A Novel Use of Peer Coaching to Teach Primary Palliative Care Skills: Coaching Consultation. Journal of Pain and Symptom Management. 2017;54(4):578-82.

25. Kawabata N, Nin M. Effect of continual quality improvement of palliative care consultation teams by iterative, customer satisfaction survey-driven evaluation. BMC Palliative Care. 2021;20(1).

26. Kennedy R, Abdullah N, Bhadra R, Bonsu NO, Fayezizadeh M, Ickes H. Barriers to Effective use of Palliative Care Services in the Acute Care Setting with Emphasis on Terminal Noncancer Diseases. Indian J Palliat Care. 2019;25(2):203-9.

27. Khateeb R, Puelle MR, Firn J, Saul D, Chang R, Min L. Interprofessional Rounds Improve Timing of Appropriate Palliative Care Consultation on a Hospitalist Service. American journal of medical quality : the official journal of the American College of Medical Quality. 2018;33(6):569-75.

28. Kyeremanteng K, Beckerleg W, Wan C, Vanderspank-Wright B, D'Egidio G, Sutherland S, et al. Survey on Barriers to Critical Care and Palliative Care Integration. The American journal of hospice & palliative care. 2020;37(2):108-16.

29. Lehn JM, Gerkin RD, Kisiel SC, O'Neill L, Pinderhughes ST. Pharmacists Providing Palliative Care Services: Demonstrating a Positive Return on Investment. Journal of Palliative Medicine. 2019;22(6):644-8.

30. Ma J, Chi S, Buettner B, Pollard K, Muir M, Kolekar C, et al. Early Palliative Care Consultation in the Medical ICU: A Cluster Randomized Crossover Trial. Critical Care Medicine. 2019;47(12):1707-15.

31. McDarby M, Carpenter BD. Barriers and Facilitators to Effective Inpatient Palliative Care Consultations: A Qualitative Analysis of Interviews With Palliative Care and Nonpalliative Care Providers. The American journal of hospice & palliative care. 2019;36(3):191-9.

32. Mertens F, Debrulle Z, Lindskog E, Deliens L, Deveugele M, Pype P. Healthcare professionals’ experiences of inter-professional collaboration during patient’s transfers between care settings in palliative care: A focus group study. Palliative Medicine. 2021;35(2):355-66.

33. Morikawa M, Shirai Y, Ochiai R, Miyagawa K. Barriers to the Collaboration Between Hematologists and Palliative Care Teams on Relapse or Refractory Leukemia and Malignant Lymphoma Patients' Care: A Qualitative Study. The American journal of hospice & palliative care. 2016;33(10):977-84.

34. Nevadunsky NS, Gordon S, Spoozak L, Van Arsdale A, Hou Y, Klobocista M, et al. The role and timing of palliative medicine consultation for women with gynecologic malignancies: Association with end-of-life interventions and direct hospital costs. Obstetrical and Gynecological Survey. 2014;69(7):400-2.

35. Nieder C, Tollåli T, Haukland E, Reigstad A, Flatøy LR, Engljähringer K. Impact of early palliative interventions on the outcomes of care for patients with non-small cell lung cancer. Supportive Care in Cancer. 2016;24(10):4385-91.

36. Oertel M, Schmidt R, Steike DR, Eich HT, Lenz P. Palliative care on the radiation oncology ward—improvements in clinical care through interdisciplinary ward rounds. Strahlentherapie und Onkologie. 2023;199(3):251-7.

37. Pan HH, Shih HL, Wu LF, Hung YC, Chu CM, Wang KY. Path modeling of knowledge, attitude and practice toward palliative care consultation service among Taiwanese nursing staff: A cross-sectional study. BMC Palliative Care. 2017;16(1).

38. Rocque GB, Campbell TC, Johnson SK, King J, Zander MR, Quale RM, et al. A Quantitative Study of Triggered Palliative Care Consultation for Hospitalized Patients with Advanced Cancer the results of Cohorts 1 and 2 were presented at the 2014 Annual Assembly of the American Academy of Hospice and Palliative Medicine (AAHPM) and the Hospice and Palliative Nurses Association (HPNA). Journal of Pain and Symptom Management. 2015;50(4):462-9.

39. Sarradon-Eck A, Besle S, Troian J, Capodano G, Mancini J. Understanding the Barriers to Introducing Early Palliative Care for Patients with Advanced Cancer: A Qualitative Study. Journal of Palliative Medicine. 2019;22(5):508-16.

40. Sasahara T, Watakabe A, Aruga E, Fujimoto K, Higashi K, Hisahara K, et al. Assessment of reasons for referral and activities of hospital palliative care teams using a standard format: A multicenter 1000 case description. Journal of Pain and Symptom Management. 2014;47(3):579-87.

41. Selvaggi KJ, Vick JB, Jessell SA, Lister J, Abrahm JL, Bernacki R. Bridging the gap: A palliative care consultation service in a hematological malignancy-bone marrow transplant unit. Journal of Community and Supportive Oncology. 2014;12(2):50-5.

42. Spalding R, Kozlov E, Carpenter BD. Words Matter. International journal of aging & human development. 2017;85(1):123-30.

43. van der Stap L, de Heij AH, van der Heide A, Reyners AKL, van der Linden YM. Barriers and facilitators to multidimensional symptom management in palliative care: A focus group study among patient representatives and clinicians. Palliative & supportive care. 2022:1-12.

44. van der Stap L, de Nijs EJM, Oomes M, Juffermans CCM, Ravensbergen WM, Luelmo SAC, et al. The self-perceived palliative care barriers and educational needs of clinicians working in hospital primary care teams and referral patterns: lessons learned from a single-center survey and cohort study. Annals of palliative medicine. 2021;10(3):2620-37.

45. Wysham NG, Hua M, Hough CL, Gundel S, Docherty SL, Jones DM, et al. Improving ICU-Based Palliative Care Delivery: A Multicenter, Multidisciplinary Survey of Critical Care Clinician Attitudes and Beliefs. Critical Care Medicine. 2017;45(4):e372-e8.

46. Yang GM, Yoon S, Tan YY, Liaw K. Experience and Views of Oncology and Palliative Care Professionals on a Corounding Model of Care for Inpatients With Advanced Cancer. The American journal of hospice & palliative care. 2018;35(11):1433-8.

47. Zemplényi AT, Csikós Á, Fadgyas‐Freyler P, Csanádi M, Kaló Z, Pozsgai É, et al. Early palliative care associated with lower costs for adults with advanced cancer: evidence from Hungary. European Journal of Cancer Care. 2021;30(6):1-10.

48. Zemplényi AT, Csikós Á, Csanádi M, Mölken MRV, Hernandez C, Pitter JG, et al. Implementation of palliative care consult Service in Hungary-integration barriers and facilitators. BMC Palliative Care. 2020;19(1).
