# Supplementary material for: Interprofessional collaboration between hospital-based palliative care teams and hospital ward staff: A realist review
Source: PLoS One. 2025 Dec 19;20(12):e0338132. doi: 10.1371/journal.pone.0338132 (PMC12716714; doi:10.1371/journal.pone.0338132)
Supplement: S5 File — (DOCX) [file pone.0338132.s005.docx]

| Supplementary file 5 |
| --- |
| Individual CMO configurations |

| Moons et al., 2025 |
| --- |

**Individual CMO configurations**

Contents

[High Relevance - High Rigour 3](#_Toc187251347)

[1. Böling et al., 2020 [24] 3](#_Toc187251348)

[2. Conolly et al., 2 – 2021 [36] 7](#_Toc187251349)

[3. Coym et al., 2020 [34] 9](#_Toc187251350)

[4. Firn et al., 2018 [45] 11](#_Toc187251351)

[5. Friedrichsen et al., 2021 [40] 14](#_Toc187251352)

[6. Jacobsen et al., [42] 16](#_Toc187251353)

[7. Khateeb et al., [28] 18](#_Toc187251354)

[8. Ma et al., [41] 20](#_Toc187251355)

[9. McDarby et al., [25] 22](#_Toc187251356)

[10. Rocque et al., [43] 24](#_Toc187251357)

[11. Selvaggi et al., [27] 26](#_Toc187251358)

[12. Van der stap 2 et al., [26] 28](#_Toc187251359)

[13. Yang 1 et al., [29] 30](#_Toc187251360)

[Medium Relevance – High Rigour 33](#_Toc187251361)

[14. Artioli et al., [23] 33](#_Toc187251362)

[15. Braus et al., [30] 35](#_Toc187251363)

[16. Courtright et al., [49] 37](#_Toc187251364)

[17. Economos et al., [35] 39](#_Toc187251365)

[18. Morikawa et al., [47] 41](#_Toc187251366)

[19. Pan et al., [53] 45](#_Toc187251367)

[20. Sarradon-Eck et al., [48] 48](#_Toc187251368)

[21. Van der Stap 1 et al., [22] 51](#_Toc187251369)

[Low Relevance - High Rigour 53](#_Toc187251370)

[22. Mertens et al., [46] 53](#_Toc187251371)

[High Relevance – Low rigour 53](#_Toc187251372)

[23. Oertel et al., [37] 53](#_Toc187251373)

[Medium Relevance – Low rigour 53](#_Toc187251374)

[24. Anandan et al., [31] 53](#_Toc187251375)

[25. Beck et al., [50] 53](#_Toc187251376)

[26. Berglund et al., [32] 53](#_Toc187251377)

[27. Cannon et al., [33] 53](#_Toc187251378)

[28. Gatta et al., [39] 53](#_Toc187251379)

[29. Kawabata et al., [38] 53](#_Toc187251380)

[30. Kennedy et al., [52] 53](#_Toc187251381)

[31. Kyeremanteng et al., [51] 53](#_Toc187251382)

[32. Zemplenyi 2 et al., [44] 53](#_Toc187251383)

# High Relevance - High Rigour

## 1. Böling et al., 2020 [24]

| **Context & Intervention:**  Participants included:  - PC specialists from PCTs (physicians, RNs, and social workers)  - ward staff receiving PCCs (physicians, RNs, social workers, and assistant nurses)  Staff from 4 public hospitals, 1 university hospital and 3 local hospitals in Sweden. Financial prerequisites differ between the PCCSs, affecting possibilities to staff the services.  PCCs is only described in the introduction and is seen as a means of knowledge translation of PC expertise to non-specialized services. It is a complex intervention taking place in complex social systems.  Focus groups were held with both participating groups (PC specialists and ward staff) separately to examine how these PCCs are practiced and perceived by both groups. 6 different ways of conducting a PCC were identified which formed the basis and catalyst for discussions:  1. Regular consultation rounds  2. Nurse led attendance  3. Telephone and paper referral 4. Educational training  5. Coaching and structured reflection  6. Multidisciplinary team meetings |
| --- |

| **Data excerpt 1:**  *“Table 3 Variations in consultation practice. A. Regular consultation rounds*   - ***What****? Regular palliative care rounds with wards* - ***How?*** *The PCCS visited wards regularly, often weekly, where they conducted a palliative round, discussing patients with palliative needs who were under care of the ward* - ***Who?*** *Providers of the consultations varied. Examples included a team comprising social worker, nurse and physician; one or two nurses; or a sole physician* - ***With whom?*** *Health care providers. Professional background varied* - ***Main purpose/function?*** *Discussion and recommendations regarding palliative care including treatment and planning for patients and ethical issues. Promoting the PCCS and making it known within the hospital. Promoting palliative care.* - ***Perceived pros and cons:*** *Meeting a perceived need - Being accessible - Enhancing collaboration - Time consuming - Challenges in calibrating what is fruitful to bring up during the consultation rounds - Sometimes challenging for ward nurses to participate in consultation rounds due to time constraints - In some cases, divergent thoughts about what the focus of the consultation should be - For some ward staff, challenging to remember the time of the palliative consultation. Could also coincide with other activities - Some mentioned the consultant was not sufficiently prepared - Not creating value”* |
| --- |

| **CMO #1:**  **Context : If** discussions and recommendations regarding PC are being held during regular consultation rounds by the PCCS on the wards  **Mechanism : then** the perceived needs among ward staff are being met  **Outcome :** / |
| --- |

| **Data excerpt 2:**  *“The perception of palliative consultations contributing a holistic approach to the patient was a prominent feature of the focus group discussions and such an approach appeared to be appreciated by health care professionals on the wards. They perceived the consultant as having wide knowledge, which supported the ward in a number of matters.”*  *“...* *This citation highlights the balance of being confident about having important knowledge and perspective to contribute, but still knowing that in order to gain access and the opportunity to share knowledge the consultant must be accepted – possibly by adopting a humble approach.”* |
| --- |

| **CMO #2:**  **Context**: If PC specialists are perceived as having a wide PC knowledge  **Mechanism**: this leads to the ward staff feeling supported in a number of ways.  **Outcome**: / |
| --- |

| **Data excerpt 3:**  *“Awareness and attention to palliative care was a shared belief as one function of the palliative consultation. Creating space for discussion about this patient group that otherwise risked being omitted was highlighted by one physician from a receiving ward, as described below. The discussion centred on how this patient group might not be receiving enough attention in ordinary care, but that this was redressed at least once a week because of the PCCS.”*  *“One PCCS and a receiving ward mentioned how by conducting symptom assessment with patients, the consultant shed light on symptoms that had been missed by the ward team. Moreover, conducting consultations was mentioned as influencing a need for more palliative knowledge among receiving staff, and making room for questions regarding palliative care. One nurse from a PCCS said:“In my view, the more we show up, the more it feels like the need is greater and greater, quite simply” …. Nevertheless, one receiving ward for whom the regular consultations were not perceived as useful, mentioned that they sometimes forgot to cancel the scheduled meeting and so raised the issue of a patient simply to have something to say. This highlights two sides of the same coin, where being present might highlight needs but may also create a feeling that wards ought to come up with questions.”*  *“All currently practicing PCCSs made regular visits to wards as a way of conducting their consultations, in this way making the PCCSs known, which was seen as a prerequisite to receiving referrals and to increasing demand for palliative care consultations over time. Moreover, the PCCSs considered a good relationship between the PCCS and the wards to be an important prerequisite so that the consultant would be recognised by name and face, and also because a collaborative and attentive approach would be more conducive to facilitating good relationships than an overbearing one.”*  *“A number of the PCCSs chose to practise recurrent palliative consultation rounds. This model was seen as a way of becoming known and fundamental to reaching out and receiving patients. As palliative consultations were rather novel in several of the contexts, it seemed logical to implement a visible model, reminding wards of their existence.”*  *“In the current study, appreciation was expressed in regards to having a regular model that “reminded” wards of this particular patient group and their needs… Comparing referrals only and regular consultations, it is possible that the repetitive component of the regular consultations drew attention to palliative care, which could possibly influence greater assistance to this patient group and their needs. Moreover, regular consultations could also be viewed as a pro-active approach, in contrast to a re-active response to referrals. Future research needs to examine how consultation practices impact patient care and palliative care knowledge use in receiving wards,”* |
| --- |

| **CMO #3:**  **Context**: When PCT is present in the care for hospitalized patients by regularly visiting hospital wards  **Mechanism**:   - Then this leads to awareness among ward staff for patient’s PC needs - Then this influences a need for more palliative knowledge among receiving ward staff - Then ward staff is reminded about PCT.   **Outcome**: / |
| --- |

| **Data excerpt 4:**  *(1) Staff turnover*  *“Some PCCSs included the definition and concept of palliative care in the educational activities they carried out. However, staff turnover was seen as an obstacle to reaching a consistent level of knowledge about palliative care, as well as awareness of the PCCS.”*  *“They highlighted the importance of palliative specialists and other health care services having a shared understanding of palliative care and how consultants can contribute to a learning process in this regard, as well as staff turnover being a barrier to learning”.*  *(2) Time constraints*  *“In demanding contexts where time is a limited resource, research has shown that palliative care may become less prioritised in comparison to acute and curative care (Lind et al., 2017; Chan et al., 2018). Indicators of similar behaviours were found in our focus groups, where staff showed appreciation for how the regular consultations put focus on patients that would otherwise not be so thoroughly discussed, and time constraints were said to hold back nurses on the ward from participating in the consultation rounds.”*  *(3) Structure of PC organization*  *“Factors affecting the structure, process and outcome of these consultations seemed to occur on different levels. Such factors included local structure of overall palliative care organisation, assigned resources and their purpose, identified needs within the hospital organisations and wards, commitment among managers, staff turnover, resources and commitment within wards and which professions participated in the consultations. Moreover, the services appeared to choose whatever method of practising the consultations they felt comfortable with or considered favourable from experience. In one focus group, a PCCS stated that the perceived absence of top-down management enabled creativity, but that it also made things somewhat unclear, and was perhaps the reason why their service had different approaches to practising consultations at different sites and over time”*  *(4) Resources, needs and commitment*  *= mentioned in data excerpt above but no other data excerpts available for extra information.* |
| --- |
| *“Factors affecting the structure, process and outcome of these consultations seemed to occur on different levels. Such factors included local structure of overall palliative care organisation, assigned resources and their purpose, identified needs within the hospital organisations and wards, commitment among managers, staff turnover, resources and commitment within wards and which professions participated in the consultations”*  ***🡪 Local PC organization, staff turnover, available resources, commitment, and PCT composition (C) influence the consultation process (M) and it’s outcomes (O)*** |

| **CMO #4:**  **Context**:   1. Staff turnover 2. If nurses do not have enough time   **Mechanism**:   1. leads to less awareness of the PCT among ward staff 2. then they are held back to participate in consultation rounds   **Outcome**: / |
| --- |

## 2. Conolly et al., 2 – 2021 [36]

| **Context + Intervention:**  Participants included staff nurses, clinical nurse managers, clinical nurse specialists, senior nursing managers, physiotherapists, medical and surgical doctors, and health and social care professionals from various departments (not specified) in an acute care hospital in Ireland.  They filled out a questionnaire to evaluate their knowledge of the Specialist Palliative Care (SPC) Clinical Nurse Specialist (CNS), afterwards a small part of the participants participated in focus groups or individual interviews to get a deeper understanding about their current perceptions of the SPC CNS role. |
| --- |

| **Data excerpt 1:**  “A key aspect of the role of a clinical nurse specialist is the provision of education to both patients and fellow clinicians about a particular condition and how best to manage it. Clinical Nurse Specialists can draw on their expert knowledge and clinical experience to provide this education. Education played an important and central role for the SPC CNS, with participants seeing this role as the most important in the context of support.”  “I suppose education is paramount, assisting them to assist the patient so giving them the tools they need to look after the patient in the absence of the team. The tools then need to I suppose identify symptoms and how to treat them effectively … ”  “A key component of this support focused on discharge planning particularly when arranging a rapid discharge, to facilitate a person’s wish to die at home” |
| --- |

| **CMO #1:**  **Context**: If PC clinical nurse specialists takes up staff training and education (provide education through their expertise based on their knowledge and clinical experience).  **Mechanism**: then ward staff feels supported in their care for patients.  **Outcome**: Ultimately leading to facilitation of patients’ wishes (such as a rapid discharge because of the patient’s wish to die at home) |
| --- |

| **Data excerpt 2:**  *“Overall respondents held positive attitudes towards the SPC CNS in relation to clinical care, education and patient advocacy. A minority of respondents (<10%) did not value audit and research as core activities of the role”*  *“I suppose education is paramount, assisting them to assist the patient so giving them the tools they need to look after the patient in the absence of the team. The tools then need to I suppose identify symptoms and how to treat them effectively … So there’s a huge emphasis or there should be a huge emphasis on education. (P5)”*  *“I think you know that MDT approach that they have is working well. (P5) … you know they will advocate for the needs or the you know the complexity that is going on with the family and what might be required or needed for them… it’s safe to say its interacting with the patient and their families, works extremely well. (P6)”*  *“Table 4:*  *The CNS in Palliative Care always plays an integral role in patient education (n=121): 86.0 (104) Strongly agree*  *The CNS in Palliative Care always plays an integral role in staff training and education (n=121): 68.6 (83) Strongly agree”* |
| --- |

| **CMO #2:**  **Context:** If a SPC CNS takes up roles such as, patient education, staff training and eduction, and is a patient’s advocate, this leads to  **Mechanism:** Good acceptance of the role with positive attitudes from the healthcare workers on the wards  **Outcome:** / |
| --- |

## 3. Coym et al., 2020 [34]

| **Context + Intervention:**  Participants included PC specialists of a multiprofessional PCT and requesting physicians from both oncology, gynecology, dermatology, nephrology and ICU departments of a university, maximum care hospital in Hamburg, Germany with 1700 beds.  PC physicians provided PCC on a regular basis within the last 12 months. Requesting physicians provided day-to-day care to inpatients with advanced, life-limiting diseases and requested PCC regularly within the last 12 months.  Inpatient PCC for patients with specialist PC needs is provided by a PCT consisting of physicians and nurses since 2017. They are available throughout the whole hospital via an online form in the patients’ EHR.  Semistructured interviews with PC specialists and regularly IPCC requesting physicians were conducted following an interview guide to identify triggers leading to IPCC, the perceived impact and the challenges and potential improvements for IPCC. 10 PC specialists and 9 IPCC requesting physicians were interviewed. |
| --- |

| **Data excerpt 1:**  *“A requesting physician associated IPCC support for symptom burden with team-based needs: “(...) the team is overwhelmed with patients in extreme pain (...)” … and a PC specialist confirmed: “(…) general wards are possibly overwhelmed by the needed time for patients and by the complexity of symptom control, psychosocial needs and the obligation of explaining that lifetime is limited (…)””*  *“Table 2: Issues leading to an IPCC request*  *- Physical symptom burden*  *- Patients’ quality of life*   - *When the treating team assumed that quality of life could be improved by IPCC*   *- Psychological distress*  *- Overstraining*   - *When family caregivers and the treating team were overwhelmed with dealing with pts*   *- …*  *- Limited staff resources*   - *Pts in need of specialized PC are often time consuming and treating teams cannot meet the needs and therefore ask for support”*   *“Table 2: Barriers on regular wards concerning treatment of patients with PC needs*  *- …*  *- Resources of the requesting team:*   - *Overstraining* - *No multidisciplinarity* - *Lack of knowledge* - *Lack of time*   *Requesting physicians can be overwhelmed by the complexity of symptoms and psycho-social needs of pts., and not competent to treat these, also regular wards lack the preferable extent of multidisciplinarity”* |
| --- |

| **CMO #1:**  **Context**: the complexity of caring for patients with PC needs + lack of time + lack of knowledge  **Mechanism**: ward staff becomes overwhelmed and then become aware they cannot meet patients’ needs  **Outcome**: therefore ask for support. |
| --- |

| **Data excerpt 2:** *“Table 2: Issues leading to an IPCC request*  *- Physical symptom burden*  *- Patients’ quality of life*  *- Psychological distress in patients and family caregivers*  *- Overstraining*  *- …”*  *“Table 2: Impact of IPCC*  *- Transfer of knowledge to the requesting team*   - *Through IPCC non-PC teams are educated in PC*   *- Relief for the requesting team*   - *Time consuming care and advice concerning palliative situations can be yield to the IPCC-team*   *- Relief for family caregivers*   - *IPCC teams include family care givers in their treatment approach which helps them to get about the situation*   *- Better patient coping*   - *IPCC supports pts. in coping with the disease/palliative situation*   *- Improvement of symptom burden*  *- Improvement of further care”*  *“PC specialists and requesting physicians likewise saw a benefit for the treating team in terms of relief by backing up the team and giving advice. As said by one of the requesting physicians: “we are very thankful for support from the IPCC with suggestions for further treatment (...) taking aspects into consideration we have not thought of. If there is no transfer option to the palliative care unit, one can always call and ask for help.” (participant number A003, female, 30–40 years old, 6 years of working experience).”*  *“Improvement of symptom management/ control (mostly perceived as an impact by PC specialists) was reported not only leading to a relief for the patient but also the treating team, as mentioned by a PC specialist: “we definitely reduce symptom burden and improve medical care. And I think the colleagues are thankful for our suggestions and support.” (participant number K008, male, 30–40 years old, 9 years of working experience, 5 years PC experience). This was also supported by requesting physicians.”*  *“Additionally, both groups, but mostly requesting physicians, observed a positive impact on patient’s coping with the situation: “after requesting IPCC, ( …) patients are less fearful concerning PC. They understand*  *that PC does not mean they have to die right away (…)”* |
| --- |

| **CMO #2:**  **Context**: /  **Mechanism**: If treating physicians see physical or psychosocial symptom burden of patients and family caregivers as an indication for PC  then they are motivated to request/involve the PC specialists.  Through their involvement advice and support is offered to the requesting team which leads to  **Outcome**:  Reduced symptom burden  Better patients’ coping with the disease  Including relatives in treatment approach helping them to get about the situation  Feeling relieved because care and advice for palliative situations can be delegated to the PCT  Transfer of PC knowledge to ward staff |
| --- |

## 4. Firn et al., 2018 [45]

| **Context + Intervention:**  In-depth interviews were carried out with **masters trained, ward social workers** to explore their perceptions of collaborating with palliative care social workers and what facilitates or hinders this collaboration.  Included in the study were English speaking ward social workers working with adult patients in both for-profit and not-for-profit hospitals with PCTs which included a palliative care social worker.  All areas of adult inpatient wards were represented in this study. And as much diversity was trying to be achieved both in setting and participants (e.g. years of working experience, hospital size, department). |
| --- |

| **Data excerpt 1:**  *“Instead, ability refers to the ward social workers’ perception of the specific clinical skills, team management competencies, and amount of experience needed to provide palliative care social work services. Perceptions of the palliative care social worker’s abilities impact the ward social worker’s willingness to collaborate.*  *“[For collaboration] trust is important about her clinical skills and abilities.” - P1.”*  *“Ward social workers perceive competent palliative care social workers to have the necessary skills to manage the ward team’s dynamics and needs, remain in a consultative role, and provide expert resources and recommendations to meet the complex needs of patients and families. These skills include: facilitate goals of care conversations, bereavement support, resource knowledge, and awareness of how to transition from standard care to palliative care, and from palliative care to hospice. Beyond these skills, ward social workers also think palliative care social workers need a good understanding of medical terminology and disease processes in order to properly guide patients and families. When the palliative care social worker is perceived to be a strong, capable clinician, trust is high and collaboration is facilitated.”*  *Other theorists, in addition to Mayer et al. (1995) [34] have also identified ability as a key component of trust [35–37]. These prior studies, again, focused on trust between superiors and subordinates where there is a clear, organisationally defined power differential between the two parties [34]. The results here add to the existing literature; indicating that even when no formally defined power differential exists, perceptions of ability are still important for collaboration with peers”* |
| --- |

| **CMO #1:**  **Context**: If ward social workers’ perceptions about the PC social workers’ abilities (skills, competencies, and experience in PC) are high  **Mechanism**:, then trust is established. This in turn leads to more willingness to collaborate.  **Outcome**: / |
| --- |

| **Data excerpt 2:**  *“Ward social workers trust palliative care social workers who demonstrate integrity. Palliative care social workers are viewed as having high integrity when they adhere to the principles of patient-centered care delineated in the professional ethics of social work.”*  *“When the palliative care social worker is perceived to value institutional needs above those of the patient, she is viewed as an agent of the institution rather than an advocate for the patient. The ward social worker does not trust or want to work with the palliative care social worker who fails to uphold patient-centered values. Lacking integrity, therefore, is a barrier to collaboration.”* |
| --- |

| **CMO #2:**  **Context**: If PC social workers adhere to principles of patient-centered care and act as a patient’s advocate,  **Mechanism**: then they are perceived by ward social workers as demonstrating integrity. This in turn leads to trust which is a prerequisite for collaboration.  **Outcome**: / |
| --- |

| **Data excerpt 3:**  *“Ward and palliative care social workers share information about cases through formal and informal verbal communication, in person or by phone, or through direct written communication via email or text-paging, as well as reading what is documented in the electronic medical record.”*  *“For communication to work well it must be two-way, timely, and consistent. Ward social workers report that collaboration is served when the ward and palliative care social workers take responsibility to each own communication and proactively communicate with one another.”*  *I think that communication is key [as a best practice] … and trying to make sure that everyone’s on the same page.” – P7.*  *When communication is lacking, delayed, inconsistent, or perceived as burdensome to the patient and family collaboration is obstructed.”*  *“… good information sharing by the palliative care social worker includes providing a summary of what occurred during the palliative care teams’ interactions with the patient, family, and ward team.”*  *“In interprofessional interactions sharing information consists of verbal, written, and non-verbal communication between team members and is demonstrated through listening, negotiating, consulting, interacting, discussing or debating with one another [51, 53]. More importantly, the findings show that the elements of good communication are similar for uni-disciplinary and inter-professional interactions. Like interprofessional teams, ward social workers from this study report experiencing higher levels of collaboration when they are given regular, formal opportunities to communicate, as well as have informal opportunities for communication.”* |
| --- |

| **CMO #3:**  **Context**: If both ward and PC social workers take responsibility in their communication and if communication is two-sided, timely, pro-active, and consistent  **Mechanism**:, then this leads to information-sharing.  **Outcome**: This process facilitates collaboration. |
| --- |

| **Data excerpt 4:**  *“Collaboration consists of three constructs: Trust, Information Sharing, and Role Negotiation (Fig. 1). Trust has three components: ability, benevolence, and integrity. All three components need to be present for Trust to occur. Positive interactions in role negotiation and information sharing can strengthen trust. However, if trust is lacking true role negotiation and information sharing cannot take place. Even when trust is present, ward social workers’ collaboration with palliative care social workers does not happen if the interaction lacks either effective information sharing or effective role negotiation. All three constructs need to be in place for collaboration to take place.”*  *“When the palliative care social worker is perceived to be a strong, capable clinician, trust is high and collaboration is facilitated… perceptions that the palliative care social worker lacks ability and is a weak clinician lead to mistrust. When trust is impaired collaboration is deterred ”*  *“When communication [information sharing] is lacking, delayed, inconsistent, or perceived as burdensome to the patient and family collaboration is obstructed.”*  *“When role negotiation does not take place or when it fails, collaboration cannot be achieved.”*  *“This is the first study to explore ward social workers’ perceptions of collaboration with their palliative care social work peers. The key constructs of collaboration are: Trust (comprised of ability, benevolence, and integrity), Information Sharing, and Role Negotiation. When all three constructs are in place and operating well, the ward social worker perceives interactions with the palliative care social worker as collaborative. When one or more pieces are missing the ward social worker does not experience interactions with the palliative care social worker as collaborative.”* |
| --- |

| **CMO #4:**  **Context**: /  **Mechanism**: If both TRUST, INFORMATION SHARING, and ROLE NEGOTATION are in place  **Outcome**: then interprofessional collaboration between ward and PC social workers takes place. If any of these three constructs is not present/limited available then their collaboration is hindered. |
| --- |

| **Data excerpt 5:**  *“With little organisational direction for either role, the challenge of time constraints, and pressures that arise from institutional mandates to facilitate discharge, a willingness to be flexible about which social worker does what task is essential for effective role negotiation. Flexibility is possible with trust. The lack of trust makes flexibility in role negotiation less possible and undermines collaboration.”*  *“However, because of the diversity of tasks and the unpredictability of patient and family care needs, ward and palliative care social workers may still need a degree of flexibility in their roles to respond to each case individually [12]. A more defined job description at an organisational level could be helpful for facilitating collaboration, as long as it allows for some flexibility, by adding additional functionality and efficiency to the ward and palliative care social work roles.”* |
| --- |

| **CMO #5: Role negotiation**  **Context**: If trust in each other is in place, then the willingness to be flexible about who does which task is strengthened  **Mechanism**: This leads to better role negotiation which in turn is favorable for collaboration.  **Outcome**: / |
| --- |

## 5. Friedrichsen et al., 2021 [40]

| **Context:**  Local hospital in the south of Sweden with 2400 employees in 2010-2012.  3 different wards participated: one internal medicine ward and two surgical wards, where a PCT was implemented and general internal medicine patients (cancer, lung diseases, kidney diseases) and general surgical patients (cancer, GI diseases, urological) in all phases of their disease were cared for. Each ward had 28 beds. Participating staff included: physicians, nurses, assistant nurses and 3 other occupations which were not specified.  **Intervention:**  PCT available for 1–8 daytime hours/week for 1 year (m=  6.5h/week). Their tasks included:  - visiting the wards and taking part in reports, rounds, and communications  - identifying patients at risk for poor outcomes, who may benefit from a PCC  - supporting primary healthcare team members.  An integrative bed-side education approach was used, where physicians and nurses specialized in PC, and experienced in PC, tried to embed PC principles and interventions into daily practice.  The primary team could also ask for specific education for all team members in the wards. Education in PC philosophy, pain management and mouth care were provided (total 48h).  The intervention was developed with the aim to enable the acute healthcare team members to make positive changes in their clinical practice. |
| --- |

| **Data excerpt 1:**  *“In the acute wards, a curative performing culture was in focus, where working fast and effectively was the goal of care with the intention of doing a good work.* ***Diagnosing and treatments were the first priorities*** *and then planning for patients discharge.* ***The value of fast work was obvious*** *in the hectic ward environment, where team members hurried between different rooms and patients,* ***indicating lack of time….***  *“…, but time was lacking in the acute culture. In communications between PCCT and the primary team members, it was clear that when primary team members were working with severely ill and dying patients,* ***they expected them to tell them if there were any problems****, without actively asking patients themselves.”*  *“The PCCT noticed the lack of time and the following values problem among all primary team members, and felt overwhelmed, especially as regards patient participation, patient autonomy, and the lack of ethical reasoning and notice in patients’ and their families’ needs.”*  *“The PCCT wanted planning and discussion with the whole team for the patients last days or weeks in life with the family present. But on rounds, there was seldom a consensus around the different primary team members, but rather divided views whether the patient was dying or not. On rounds, unpleasant decisions about PC were postponed or avoided, sometimes just an hour before the patient’s death, as the primary team did not have time for reflection around the patient’s whole situation and wanted to do as much as possible before “giving up”.*  *“There was also a fear among primary team members of being criticized for making the wrong decision regarding PC, of not having done enough and examined a patient fully.”*  *“Most primary nurses and assistant nurses showed an understanding of the PC. In confidence, they agreed that the major problems were the physicians’ lack of knowledge or skills in PC, and an inability to listen to them, of which, unfortunately, physicians remained unaware. Consequently, patients were not relieved of pain, no decisions about resuscitation were made, and no communication to patients and family members about the transition from curative to PC at end- of life was provided, which in turn led to patients dying alone. Therefore****, they expected that the PCCT would give them support in their thinking about PC****, especially in telling physicians how and when to decide about PC.*  *“Continually, the primary team members wanted to ensure the PCT that they could not practice specialised PC, since they did not have time to do so.”* |
| --- |

| **CMO #1:**  **Context**: Curative performing culture from primary team members who collaborate with PCCT: If primary care team members prioritize their care towards actively treating their patients,  **Mechanism**: this results in a feeling of lack of time to provide specialized PC which in turn leads to the expectation the PCT should give them support in their thinking and directly tell them if there are any problems.  **Outcome**: / |
| --- |

| **Data excerpt 2:**  *“The content of PC, and how to achieve good PC* ***was interpreted quite different*** *between the PCCT and primary team members,* ***which made the different teams to collide.”***  *“No one spoke with the patient and his/her family, and knew nothing about their preferences regarding death and dying.* ***This was not in line with the PCCTs’ view of good PC and made them frustrated****.”*  *“PCCT, consultant B (F): What you say now is very important;* ***how you see, what PC is will influence your decisions.***  *Primary consultant IMW 10 (M): Well, that is the dilemma here.*  *PCCT, consultant B (F): This man cannot be healthy again, he is incurably ill, but you still have not reach the breaking point.*  *Primary consultant IMW 10 (M): No* ***(firm).***  *PCCT, consultant B (F): Still you want to relieve his symptoms and improve quality of life.*  *Primary consultant IMW 10 (M): Yes, we use restricted, limited care, which is what it is (hesitates).*  *PCCT consultant B (F):* ***Then it is obvious (annoyed).***  *Primary consultant IMW 10 (M): Many patients is not in the terminal stage and do not get PC. PCCT, consultant B (F): But they ought to (determine). Observation from a sitting round”* |
| --- |

| **CMO #2:**  **Context**: When different views on content of PC and how to achieve good PC exists between ward staff and specialist  **Mechanism**:, this leads to misunderstandings, frustrations and poor communication.  **Outcome**: / |
| --- |

## 6. Jacobsen et al., [42]

| **Context & Intervention:**  A 6-week pilot peer coaching program delivered by a PC attending physician with more than 2 years of clinical practice in PC. The intervention took place in a 1000 bed urban academic medical center.  Participants included hospitalist attending physicians, residents, mid-level providers (non-physician practitioners), and medical students.  Over 6 weeks, 5 PC attendings completed 28 coaching encounters on 24 patients with 32 different learning goals about pain and symptom management, communication, and hospice.  Coachees could initiate a coaching encounter by requesting coaching through a pager or by asking the coach in person. During this coaching moments, participants had an exploratory discussion with the coach so he could better understand the participant’s (coachees’) perspective on the problem so a learning goal could be defined. Afterwards, the participant was taught the knowledge or skill he/she needed to solve the problem. Ideally, instruction focused on one teaching point. In case of communication questions, the coach did a joint visit to observe the participant and provide him/her with feedback.  Peer coaches routinely stopped by the intervention floors to remind clinicians of the pilot and to offer impromptu coaching. Standard palliative care consultation remained available to all clinicians throughout the pilot. |
| --- |

| **Data excerpt 1:**  *“Coachees articulated 32 different learning goals about pain and symptom management (44%), communication (34%), and hospice (22%).*  *🡫*  *“Fifty percent of coaching encounters resulted in symptom management medication recommendations, 39% of encounters resulted in teaching of communication skills, 29% of encounters resulted in assistance with care transitions”*  *“Through content analysis, we identified three elements of peer coaching that coachees found most useful: easy access to expertise, tailored teaching, and being in partnership.”*  *“Table 1:*  *- Easy access to expertise*  *‘‘Easily approachable, convenient, and quick to respond. I was easily able to talk my case through and get immediate feedback. Additionally, I got an email with references for future cases. Most helpful!’’*  *‘‘[the coach] was very knowledgeable and provided great ideas . It was also highly time efficient and convenient for our team to have her stop by in the morning.’’*  *- Tailored teaching*  *“I find it helpful to review symptom management with someone else to confirm whether there are additional suggestions. Over time, this is also helping expand my knowledge base and my own practice.’’*  *- Being in partnership*  *‘‘It’s probably pretty hard to track people down on the floors, but I do think that what you are doing (finding people in person) is greatly appreciated by the hospitalists.’’* |
| --- |

| **CMO #1:**  **Context**: If PC specialists (coaches) are easily accessible and respond quickly to the request by for example being present on the hospital ward during morning rounds,  **Mechanism**: then a feeling of partnership is created.  **Outcome**: This in turn leads to teaching and assistance opportunities for example in teaching communication skills and assistance with care transition. |
| --- |

| **Data excerpt 2:**  *“21% of encounters involved a joint visit with the coach, learner, and patient.”*  *“Unlike peer coaching in the education setting, our medical peer coaching model required that after talking to the coachee, the coach saw and examined the patient. These extra requirements highlight an everpresent tension between the need to create sustainable, scalable practice models through billing and the needs of clinicians, patients, and families. Meeting with the patient, however briefly, often added to the coaches’ conceptualization of the case and increased the sense of partnership between the coach and coachee. We also note that it is often hard to predict when seeing the patient will add key information to inform the plan of care. For these reasons, we believe that having the coach meet with the patient is critical to the success of this peer coaching model. We suggest that this blend between peer coaching and consultative care requires a new name, such as coaching consultation, which better acknowledges the peer coaching and consultative components of this approach.”* |
| --- |

| **CMO #2:**  **Context**: palliative care attending physician that works bedside with the patient: If PC specialists meet with patients during the coaching sessions,  **Mechanism**: then their understanding of the patient’s case is increased. In turn leading to an increased feeling of partnership between the specialist (coach) and ward staff (coachee).  **Outcome**: / |
| --- |

| **Data excerpt 3:**  *“Table 2:*  *Educational opportunity*   - *Coach is approached as an expert* - *Referring clinician wants teaching on a specific topic* - *Learning goals are identified by the referring* - *More likely to be residents* - *Includes questions on pain and symptoms, communication, or hospice*   *Collaborative Opportunity*   - *Coach is approached as a colleague* - *Referring clinician wants direction on the next step in clinical care* - *Learning goals identified by coach based on clinical care need* - *More likely to be attending physicians* - *Includes questions on pain and symptoms, communication, or hospice”*   “Coaches met weekly to reflect on their approach to coaching. Through these discussions, the coaches developed a hypothesis that coachees approach peer coaching in one of two ways, either as 1) a learning opportunity or 2) a collaborative opportunity (Table 2). Coachees who primarily view coaching as a learning opportunity understand their knowledge deficit and approach the coach for specific information. Coachees who primarily view coaching as a collaborative opportunity are trying to figure out what to do next in the clinical care of their patient and approach the coach as a colleague who might help think through the problem.” |
| --- |

| **CMO #3:**  **Context** : If ward staff knows they have a PC knowledge deficit  **Mechanisms** : they are open for learning during the interaction  **Outcome** : / |
| --- |

## 7. Khateeb et al., [28]

| **Context & Intervention:**  Study patients were cared for on the hospitalist service consisting of 11 teams, each staffed by an attending hospitalist without mid-level providers or residents, each team capped at 11 patients.. In addition, 25 hospitalists from the intervention group participated in a survey to evaluate perceived value and/or burden of the intervention.  Usual care included daily discharge rounds to discuss hospitalization goals and anticipated discharge needs. For the intervention, the scope of these discharge rounds was expanded by adding an attending and social worker from the PCT twice weekly. During these enhanced rounds, all patients on the hospitalists’ service were discussed and informal recommendations on symptom management to goals of care and advice on whether patients could benefit from a formal inpatient consultation were provided.  Prior to the intervention, PC consultation was requested ad hoc by hospitalists in reaction to patients’ symptoms or need for complex goals of care discussions. |
| --- |

| **Data excerpt 1:**  *“The intervention consisted of enhancing the scope of the discharge rounds by adding an attending and social worker from the PC team twice weekly. During enhanced rounds, all patients currently on the hospitalist’s service were discussed. PC consultants spent approximately 15 minutes of time per team meeting. If appropriate, the consultant offered informal recommendations concerning whether a patient would benefit from formal inpatient consultation. PC providers utilized clinical judgment to make recommendations about which patients could benefit from PC services rather than using a specific screening tool. Recommendations ranged from symptom management to goals of care. The recommendation to obtain a consult was not necessarily a self-referral; new PC consults are distributed across the 2 PC consultation teams through an intake pager, and in all cases, hospitalists made the final determination whether or not to pursue an inpatient consultation.”*  *“For the intervention teams, the unadjusted proportion receiving PC consultation prior to the intervention (in 2013) was 2.7%, which increased to 5.2% after implementation of the multidisciplinary team intervention (in 2014, p< .05 comparing intervention vs control)”*  *“The predicted proportion receiving a PC consult increased among non-cancer hospitalizations (n=13,356) by 1.38% over the study period for the intervention patients, compared to a 0.38% increased proportion receiving consults among the control patients”*  *“Fifteen of the 25 hospitalists participating in the intervention completed the survey (60% response rate). All respondent hospitalists reported that the intervention facilitated easier communication with PC consultants, with 83% agreeing that it added value to patient care. Only one hospitalist (out of 15) found the intervention to be too time consuming, whereas 55% said the intervention improved their own PC skills.”*  *“In conclusion, scheduled shared interprofessional rounds between hospitalists and PC specialists increased utilization and timeliness of PC consultations, particularly among noncancer patients. Regular discussions between hospitalists and PC providers improved appropriate use of PC services and hospitalist PC skills.”*  *“We hypothesize that this intervention successfully identified patients with unmet needs who would have been missed using a screening tool alone. Conversely, by applying interprofessional perspectives during rounds, we may have determined that formal PC consultation was unnecessary for patients who otherwise would have screened positive.”* |
| --- |

| **CMO #1:**  **Context**: When PC specialists are involved during discharge rounds communication among ward staff and PC consultants is facilitated. In addition, by actively being present during rounds the PC consultants can use their clinical judgement to make informal recommendations to the ward staff.  **Mechanism**: This in turn leads to a better understanding of the palliative care needs of (non cancer) patients among ward staff, in turn leading to a better decision-making among ward staff to pursue a formal PCC.  **Outcome**: increased utilization and timeliness of PCCs |
| --- |

## 8. Ma et al., [41]

| **Context:**  Participants were patients admitted to the medical ICU with high risk for morbidity and mortality based on a screening tool with 9 pre-determined PC criteria (=triggers). The teams are supervised by intensivists.  **Intervention:**  Early, triggered PCC.  = Patients who screened positive for at least one trigger criteria received a PCC within 48hours of admission.  The consultation itself consisted of regular visits by an interprofessional PCT to the primary care team. During a consultation patients’ and families’ needs were identified. Then afterwards a discussion with the primary care team was held on how to best meet those needs as well as communication between all parties with respect to goals, values, and treatment decisions took place. After an initial evaluation, a care plan for each consultation was discussed by the entire PCT on rounds, with additional team members participating as appropriate. Follow-up was carried out until discharge from the hospital.  The control arm received usual care where the palliative care physician could be consulted when requested by the medical ICU clinicians. |
| --- |

| **Data excerpt 1 :**  *“Patients in the intervention arm received a palliative care consultation within 48 hours of MICU admission. This consultation was comprised of regular visits by an interprofessional palliative care team including a physician board-certified in palliative care, nurse practitioners, a palliative care clinical fellow, a social worker, and a chaplain. A palliative care consultation included: chart review of the patient’s hospitalization, meeting with the patient and available healthcare proxies, identification of physical and emotional needs of the patient and family, discussion with the primary team on how best to meet those needs, and communication between all parties with respect to goals, values, and treatment decisions. Formal meetings including the palliative care team, primary team, and the patient or healthcare proxies were encouraged but not mandatory. A board-certified palliative care physician or nurse practitioner performed the initial evaluation, and a care plan for each consultation was discussed by the entire palliative care team at rounds, with additional team members participating as appropriate. The palliative care team continued to follow the patient until discharge from the hospital. The control arm received standard of care: palliative care could be consulted at the discretion of the MICU clinicians.”*  *“The primary outcome was the proportion of patients who transitioned to DNR/DNI resuscitation preference prior to hospital discharge, where DNR/DNI specifically refers to both Do-Not-Resuscitate and Do-Not-Intubate. Secondary exploratory outcomes included MICU LOS, hospital LOS, discharge to hospice, duration of mechanical ventilation, duration of vasopressors, tracheostomy, cardiopulmonary resuscitation, mortality, operating cost, post discharge emergency department visits, and hospital readmissions”*  Primary OC  *“The number of patients transitioned to DNR/DNI was significantly higher in the intervention group (50.5%) compared to the usual care group (23.4%) with a risk difference (RD) of +27.0% (95% CI, 13.6% to 39.1%; p<0.0001). Kaplan Meier curves showed that transition to DNR/DNI occurred earlier and more frequently over the thirty days from study enrollment in the intervention group compared to the usual care group (p<0.0001, Log-Rank Test) (Figure 1).”*  *“The interprofessional palliative care team interacted with the patient, family, and primary team on multiple occasions throughout the admission to facilitate symptom relief, set appropriate expectations, and address goals of care. Code status changes were significantly increased in the intervention group, even after controlling for potential confounders, and as expected there were improvements in multiple other clinically important metrics likely driven by effective goals of care discussions.”*  Secondary OCs  *Transfer to hospice care occurred significantly more often in the intervention group compared to the usual care group (18.6% vs 4.9%; p=0.0026) (Table 3). Median duration of mechanical ventilation was shorter by 2 days in the intervention group compared to the usual care group (4 vs 6 days; p=0.0415). Performance of a tracheostomy during the hospitalization was less in the intervention group compared to the usual care group (1.0% vs 7.8%; p=0.0354). There was no significant between-group difference for hospital mortality (21.7% vs 27.5%), 30-day mortality (35.1% vs 36.3%), ICU LOS (median 5 vs 5.5 days), or hospital LOS (median 10 vs 11 days) (p values>0.05)…. Of the patients that survived until discharge, fewer patients in the intervention group compared to the control group presented to an emergency department within 30 days after discharge (1.3% vs 12.5%; p=0.0067), were admitted to the hospital (17.3% vs 33.3%, p=0.0236), or required either hospital readmission or an emergency department visit over the same time period (17.3% vs 38.9%; p=0.0028).”*  *“The total operating cost per patient in the intervention group was $37,310 compared to $45,790 in the control, which was not significantly different (p=0.1353)…. When analyzing total operating cost by department, the intervention group had significantly lower MICU ($9,860 vs $15,660; p=0.0038) and pharmacy ($3,430 vs $5,850; p=0.0158) costs per patient compared to the control group (Figure 2, Supplemental Table 2).”* |
| --- |

| **CMO #1:**  **Context:** patients admitted to a medical ICU with a positive trigger for high risk at morbidity and mortality, who receive an early PCC (within 48hours of admission).  **Mechanism:** If the PCT is consulted early on in the admission of the patient, then they visit the patient regularly. This leads to a regular interaction between PCT, patients and primary care team through which appropriate expectations are set and goals-of-care discussions are held. These discussions are (most likely) the drivers leading to …  **Outcome:** increased patients with DNR codes, an increase in hospice referral, a decrease in certain costs (MICU and pharmacy costs) and a decrease in medical resource utilization (less longer duration of mechanical ventilation, less performance of tracheostomy, fewer ED visits and hospital (re)admissions). |
| --- |

## 9. McDarby et al., [25]

| **Context & Intervention:**  Participants included 19 PCT providers (physicians, nurses, nurse practitioners, social workers, and 1 chaplain) and 29 nonpalliative specialist providers (28 physicians and 1 nurse practitioner) who regularly or moderately consulted the PCT.  Semi-structured interviews were held with questions designed to gather information about team interactions, the qualities of “successful” and “unsuccessful” PCCs, reasons providers might consult the PCT, responsibilities performed by a PCT, and types of recommendations requested from and made by the PCT.  With this study, researchers wanted to consider the extent to which professional interactions influence specific aspects of the consultation process in order to gain a better insight into factors that may influence successful team collaboration. |
| --- |

| **Data excerpt 1:**  *“Both PCCT and non-PCCT providers discussed the key role of visibility of the PCCT team, in both*  *facilitating new consultations and maintaining positive relationships between providers.”*  *“One provider described her colleague’s strategy of “chart[ing] in the area where she was more visible” to reinforce the PCCT’s presence (Table 4). Providers also discussed unintentional visibility (eg, passing each other in the hallway) as unplanned, but equally important.*  *Table 4:*  *“She would sit out and chart in the area where she was more visible, because ... if we’re walking by and somebody sees us, it prompts [them]. When you’re in front of them, on the floor, we’re more apt to pick up another one.’ (PCCT registered nurse)*  *“Because obviously if you see Dr X [PCCT physician] up there talking about palliative care, it keeps it in your mind which one of your patients might benefit, more in real time.” (Colorectal surgeon)*  *“Participants in this study described other strategies that could facilitate the receipt of new consultations and the consultation process, including strategic visibility, which has also been identified by providers in previous research.13 Palliative care providers might consider using this technique more intentionally in order to maximize contact with other providers and maintain a presence throughout the hospital.”* |
| --- |

| **CMO #1:**  **Context**: if PCT makes itself **visible (through certain actions** either intentional or unintentional),  **Mechanism**: then ward staff is prompted/reminded about PC  **Outcome**: this leads to the request of new consultations and the maintenance of a positive relationship between the PCT and ward staff |
| --- |

| **Data excerpt 2:**  *“Providers described patient and family confusion about palliative care as an impediment to successful collaboration, both in terms of requesting an initial consultation and during collaboration. A family’s active resistance to the PCCT’s involvement in care could prevent providers from seeking out a consultation altogether or affect involvement of the PCCT after a consultation is requested.* |
| --- |

| **CMO #2:**  **Context**: : If patients and families are confused about or show resistance to PC  **Mechanism**, then this prevents ward staff from getting into contact with the PCT or it affects PCT involvement after a consultation was already requested.  **Outcome**: / |
| --- |

| **Data excerpt 3:**  *“Palliative care consultation teams providers also described the value of demonstrating their relevance across specialties: They tend to receive consults from providers who understand how palliative care can support their patients’ needs. They stated that cultivating positive views about the service and its utility for different specialties via exposure could facilitate the consultation process.”*  *“Participants also suggested that collaboration is enhanced when providers have a clear understanding of the utility of palliative care. Beyond targeted educational interventions, providers cited the value of “marketing and promoting,” via case presentations and in-services, so that colleagues better understand how the PCCT could complement their specialty practice. However, …”* |
| --- |

| **CMO #3:**  **Context**: If PCT demonstrates their relevance  **Mechanism**: then this cultivates positive views about the PC service and better understanding about the utility of PC  **Outcome**: which enhances collaboration. |
| --- |

| **Data excerpt 4:**  *“Both PCCT and non-PCCT providers’ descriptions of the roles and responsibilities of the PCCT highlighted discrepancies in perspective that may not only obstruct providers from seeking the input of the PCCT but could also reduce meaningful collaboration once the consultation process is underway. For example, almost all PCCT and non-PCCT providers agreed that facilitating communication is a key role of the inpatient PCCT. However, there was a divergence in responses between providers in terms of what communication with families should entail, as well as* ***ambiguity about requesting providers’ true reasons for consulting and the PCCT’s perceived reality about the services they can and should provide****.”* |
| --- |

| **CMO #4:**  **Context**: If roles and responsibilities of the PCT are unclear,  **Mechanism**: then this hinders ward staff from seeking contact with the PCT and could also reduce meaningful collaboration once the consultation process is going.  **Outcome**: / |
| --- |

## 10. Rocque et al., [43]

| **Context & Intervention:**  A quality improvement initiative was set up in this study, in which a triggered palliative care consultation was implemented for patients with advanced cancer and an unplanned admission, regardless of performance status. Advanced cancer was defined as having incurable disease or distant organ-based metastases.  **The triggered PCC** were conceptually an abbreviated consultation focused on illness understanding, prognostic awareness, physical symptoms, and goals of care. On completion of triggered PCC, the lead physician on the PC team provided an abbreviated written summary of the discussion to both the patient and the patient’s primary outpatient oncologist. The primary oncology team managed symptoms, and the PC staff member intervened only when the oncology team requested assistance or when patients raised specific symptom concerns.  The study consisted out of three different cohorts:  - Cohort 1 = control cohort  - Cohort 2 = first cohort after implementation of triggered PCC  - Cohort 3 = cohort that received a modified consultation based on identified barriers.  During Cohort 2, the inpatient PC physician staffed the triggered oncology consults in addition to the inpatient unit and any PC consults requested throughout UW hospital. In Cohort 3, staffing was increased such that one physician was responsible for the TPCC and the inpatient unit, whereas a second physician staffed non oncology consults.  In Cohort 3, one staff member, typically the attending physician, performed the consults. This staff member attended oncology multidisciplinary rounds with nursing staff, social workers, and case managers when available to assist in identification of eligible patients and enhance routine communication between the oncology and PC teams.  Content of the consult differed between cohort 2 en 3:  In Cohort 2, the consult heavily emphasized symptom management and quality of life.  In Cohort 3, triggered PCC focused on disease understanding, goals of care, and the importance of having a health care power of attorney (POA). |
| --- |

| **Data excerpt :**  *“In Cohort 1, 12% of patients had a PC consult requested by the oncology team. Consults were performed in 60% of patients in Cohort 2 and 62% in Cohort 3, short of our goal of 100%.”*  *“Disease understanding during index admission was improved in Cohorts 2 and 3, with 94% (46 of 49) of patients correctly identifying that their cancer was incurable compared with 65% (15 of 23) in Cohort 1 (P ¼ 0.002). This difference persisted at eight weeks (83% vs. 63%); however, it was not statistically significant (P > 0.05)… Although disease understanding was improved, there was no change in health-related quality of life, symptom burden, or satisfaction (P > 0.05).”*  *“Despite the abbreviated and focused nature of these consultations, the percent of patients able to identify that their disease was incurable increased from 65% to 94%. These surveys could be completed at any time during their index hospitalization including after the patient completed the TPCC; therefore, we believe the increase is because of the consult resulting in enhanced communication between the patient and both the PC provider and the oncologist because, in many instances, the PC consultant would work with the oncologists to inform them about existing misunderstandings so they could address those with the patient.* *The integration of PC into regular oncology care supports an effective partnership between PC providers and oncologists, which may enhance the oncologist’s skills and comfort with communicating the seriousness of a patient’s illness.”* |
| --- |

| **CMO #1:**  **Context**: During the consultation communication between patients, oncology team and PC staff is enhanced,  **Mechanism**:, which leads to a partnership that in turn may enhance oncologists’ skills and communication.  **Outcome**: patient’s improved understanding of their illness. |
| --- |

## 11. Selvaggi et al., [27]

| **Context & Intervention:**  The intervention took place at a hematological oncology inpatient unit. The unit staff includes 6 attending physicians, 12 fellows, 30 nurses, 1 care coordinator, 1 social worker, and 2 transplant coordinators.  Quality improvement program in which PC providers were embedded into a hematological malignancy unit in a tertiary care hospital were no previous PC program existed.  Participants included both:  - patients with hematological malignancies in which pain, hospice referral and GoC discussions were evaluated  - physicians from the HM-BMT unit which filled out a satisfaction survey to assess acceptability, usefulness and effectiveness of the PC program  The PC program consisted of:  - education sessions  - clinical consultations in which PCT frequently (not specified how much) joined on rounds, attended the weekly interdisciplinary meetings, had discussions with primary physicians, made recommendations and communicated these to the assigned professional. In addition one PCT member was always present for 2h every day.  Once clinicians were aware of the existence of the PC service, oncologists began referring patients to the PC clinicians and consultations were performed. |
| --- |

| **Data excerpt 1:**  *“**Once clinicians were aware of the availability of PC services, oncologists began referring patients to the PC clinicians and standard palliative care consults were performed. Frequently, the PC team joined the attending HM-BMT physician and fellows who made rounds on these patients. Each patient who had been referred for conversations regarding goals of care was discussed with the primary hematology physician to understand the trajectory of the patient’s illness, to determine the patient’s understanding of the prognosis, and to know if any goals of care discussions had been initiated in the outpatient setting. Members of the HM-BMT team were invited to participate in discussions with patients and families regarding end-of-life decision making, referrals to hospice, and goals of care.”*  *“Overall, 41% (n = 104) of the 256 unique patients seen by our program had a DNR/DNI order entered over the course of this initiative. Of the 165 patients who had consults and who died during the period of program implementation, 41% (n = 67) were referred to hospice programs (Figure 3); the decision was always made with both the PC service and the HM-BMT services collaborating, so it is impossible to say who initiated the referral. Prior to the implementation of the program, fewer than 5% of HM-BMT unit patients who died were referred to hospice by the hematology physicians. (At the time, a consultative PC service did not exist.)”*  ***“****In all, 70% (n = 194) of the 278 consultations done by the PC team for pain control were for patients who had unacceptable or very unacceptable pain at baseline. Of these 194 consults, 66% (n = 129) reported pain levels that were acceptable or very acceptable within 48 hours of consultation. Twenty percent (n = 39) of these patients referred for pain control because they initially had unacceptable or very unacceptable pain, later had reports of pain levels that were acceptable within 72 to 96 hours of the initial consultation. Of these, 13% (n = 26) had difficult pain syndromes that were not well controlled within 96 hours.”*  *Overall, by creating a PC consultation service in an HM-BMT unit of a tertiary care hospital in Pittsburgh, we increased the number of hospice referrals on the unit, conducted the first discussions of goals of care with a majority of patients seen, and succeeded in controlling the pain of the great majority of patients seen by the PC providers.”* |
| --- |

| **CMO #1:**  **Context**: PCT who is embedded in the HM-BMT unit, carrying out clinical consultations together  **Mechanism**: they make shared decisions regarding patient care which leads to  **Outcome**: an increase in DNR/DNI orders, more hospice transfers, and a better pain management. |
| --- |

| **Data excerpts 2:**  *“… and through intensive upfront educational efforts such as ours, they may raise the bar of generalist PC competency. Indeed, this appears to be what took place during our program; by the end of the period of program implementation, hematological oncologists consulted and trusted the palliative care nurse practitioner (SAJ) – who had no oncology-specific training – just as they did the clinician with highly specific HM-BMT and PC training.”*  *“Our PC program was well received by the HM-BMT physicians, as revealed by the satisfaction surveys, especially in the areas of communication skills, psychosocial support, and end-of-life care discussions.”*  *“The researchers concluded that close ties between PC and hematology resulted in earlier referrals and more time for clinicians to address complex issues”* |
| --- |

| **CMO #2:**  **Context**: PCT who is embedded in the HM-BMT unit, carrying out clinical consultations together  **Mechanism**: leads to trust in PC nurse practitioners among oncologists  **Outcome**: / |
| --- |

| **Data excerpts 3:**  *“Once clinicians were aware of the availability of PC services, oncologists began referring patients to the PC clinicians and standard palliative care consults were performed.”*  *“Our PC program was well received by the HM-BMT physicians, as revealed by the satisfaction surveys, especially in the areas of communication skills, psychosocial support, and end-of-life care discussions.”*  *“The researchers concluded that close ties between PC and hematology resulted in earlier referrals and more time for clinicians to address complex issues”* |
| --- |

| **CMO #3:**  **Context**: /  **Mechanism**: If clinicians are aware of the availability of PC services  **Outcome**: oncologists refer patients to PC clinicians and PCC are performed |
| --- |

## 12. Van der stap 2 et al., [26]

| **Context + intervention:**  The Leiden University Medical Center introduced a PCCT In 2012 consisting of nurses, nurse practitioners and physicians specialized in palliative care. Inpatients and outpatients can be referred by physicians and nurses of primary care teams, and by self-referral of patients and their family members. All referred patients are discussed in weekly multidisciplinary team meetings involving PCCT consultants, clinicians from the patient’s primary care team, social workers, liaison nurses, medical psychologists, pharmacologists, spiritual counsellors, medical oncologists, radiation oncologists and pain specialists.  Study contains 3 parts:  Part A: Survey on barriers, educational needs an awareness of palliative support options as perceived by nurses and physicians of the hospital departments (2012).  Part B: Cohort study to evaluate timing of referral, observed survival of referred patients to identify early palliative care (≥3 months before death), late palliative care (≥2 weeks to <3 months before death) or care in the dying phase (<2weeks before death) (2012-2017).  Part C: Clinical prediction of survival at referral (physicians were asked at referral if they would be surprised if their patient would die within 1 year, 3 months or 2 weeks to assess their ability to prognosticate). |
| --- |

| **Data excerpt 1:**  *“When our PCCT started in 2012, efforts were made to design a comprehensive nonclinical strategy. Core components were educational activities in each hospital department, marketing the added value of the PCCT and implementing palliative care support options for primary care teams.”*  ***“Multiple choice questions on awareness of palliative care support options.***  *Familiarity with the PCCT increased from 56% in 2012* *to 85% in 2016 (P<0.001). In 2012, 18% of the respondents consulted the PCCT frequently and this increased to 35% in 2016. Likewise, the number of respondents who rarely or never consulted the PCCT decreased from 56% in 2012 to 32% in 2016 (P<0.001).* *In 2012, 8% of respondents appraised their experiences with the PCCT as excellent and 71% as good, and this increased to 40% and 49% of respondents in 2016 (P<0.001).”*  *“****Part B: cohort study - Palliative care referral characteristics.***  *Referrals increased by a mean of 28% per year (Figure 1).”*  *“Both barriers and educational needs were largely similar in the two surveys with 5 years in between despite increased awareness and use of palliative support options and a steady yearly increase of PCCT referrals.”* |
| --- |

| **CMO #1:**  **Context:** (Time of existence of PC) How longer a PCT exists  **Mechanism:** the more awareness about PCT increases, leading to  **Outcome :** more familiarity with PCT, increase in positive experiences with PCT, increase in ward staff who consulted the PCT frequently, decrease in ward staff who rarely or never consulted the PCT and an increase in PC referrals. |
| --- |

| **Data excerpt 2:**  *“The proportion of early palliative care referrals (OS after referral ≥3 months) increased over time only in referrals made by medical oncology clinicians (P=0.016) (Figure 2).”*  *“Evaluation of our referral characteristics confirmed that clinicians tend to seek less PCT support for non-cancer patients than they do for cancer patients (21,36-41). It is easier to identify palliative care needs in cancer patients because they usually have steady disease progression with a distinct terminal phase. In contrast, patients with organ failure usually decline gradually with acute exacerbations that may or may not lead to death, and death is often seemingly unexpected (53,56-58).”* |
| --- |

| **CMO #3:**  **Context:** If PC needs among patients are difficult to identify  **Mechanism:** then ward staff tend to seek less support from PCT  **Outcome : /** |
| --- |

## 13. Yang 1 et al., [29]

| **Context & Intervention:**  **the SPARK co-rounding model** was piloted in a large hospital in Singapore. The model consisted of a specialist PC physician + APN + internal medicine resident who joined the oncology rounding team to provide PC alongside the oncology doctors and nurses as integrated members. (= PC specialists are an integrated member of the primary care team)  This study describes the findings of one-on-one interviews with both oncology and palliative healthcare professionals. Participants included 8 physicians and 3 nurses of which 9 specialized in medical oncology and 2 in palliative medicine. They were asked about their experience of and views on the SPARK co-rounding model of care in contrast with usual care. |
| --- |

| **Data excerpt 1:**  *Accessibility*  *“Oncology doctors involved in the SPARK corounding model described a typical lag time to getting palliative care input in the usual consult service and how this was shortened in the SPARK corounding model.”*  *“Having a palliative care nurse present during the ward rounds meant that there was someone immediately available* *to guide ward staff in the administration of drugs or education of patients and caregivers.* *However, some oncology participants expressed concerns that the accessibility to the palliative care team may create a potential risk of overreliance on the palliative care services…. Nonetheless, it was mentioned by many other participants that the ready availability of palliative care advice did* *provide an opportunity to share palliative specialist knowledge.”*  *“We don’t need to wait for several hours before we get our palliative colleagues to come over. When we have our palliative colleagues there during the rounds, sometimes we can actually address the issues almost immediately. (Oncology doctor 1)”*  *The presence of the palliative care nurse was also thought to facilitate swifter palliative care interventions.”*  *“In addition to providing patients with concurrent care by oncology and palliative teams, accessibility of palliative care expertise during daily clinical work in the corounding model served as an avenue for oncology doctors and nurses to gain palliative care knowledge and skills.”*  *Communication*  *“Consequently, miscommunication created discordance between teams and subsequently “a lot of back and of forth” with repeated queries and clarifications between both teams. In contrast, the SPARK corounding model utilized a more “direct” form of communication, and there was improved clarity about the treatment plans. Questions could be clarified “there and then,” instead of having to “second guess” the other team’s likely response. Both the palliative care and oncology participants expressed that rounding together increased mutual understanding and respect between teams.”*  *“However, other participants expressed that the difference in opinions was not a major issue and could be resolved satisfactorily with face-to-face communication in a corounding. Recognising that both teams have valid perspectives, discussing the differing views was regarded as part of the process to “really get to the crux of what’s happening with the patient”.”*  *Parallel workflow*  *“The corounding model was seen as something that offered an avenue to “work in parallel instead of a sequential workflow” (Oncology doctor 2), facilitating early identification and review of patients who needed palliative care input. Face-to-face discussions about cases between 2 teams as well as brief introductory conversations with patients and families during corounding reduced the time needed to review patients’ history and further allowed the palliative care team to use their time to reach more patients. By and large, most participants acknowledged the positive impact of concurrent palliative care on the care delivery in the SPARK corounding model.”*  *Outcome*  *“The corounding model improved efficiency of palliative care delivery as a result of accessibility to palliative care expertise, effective team communications, and parallel workflow.”* |
| --- |

| **CMO #1:**  **Context**: PC specialists who are integrated in the primary care oncology team using a co-rounding model  **Mechanism**: If a co-rounding model is in place, then this leads to three mechanisms:  - It improves the accessibility to PC for ward staff which is a way for ward staff to gain more PC knowledge and skills.  - In addition, through co-rounding a more direct form of communication is established which leads to improved clarity, mutual understanding and respect between team.  - Lastly it creates a parallel workflow which facilitates early identification of patients with PC needs  All these three mechanisms together are perceived as contributing to  **Outcome**: an improved efficiency of PC delivery. |
| --- |

| **Data excerpt 2:**  *“Overall, participants felt that the SPARK co-rounding model improved quality of patient care as a result of holistic approach to cancer care and rapport building with patients and their families. Holistic approach to cancer care. The SPARK co-rounding model was felt to help facilitate the management of the broader problems experienced by patients with advanced cancer. The “different perspectives” (Oncology doctor 1) of palliative care professionals helped to “add another dimension” (Oncology doctor 5) to the patient’s care.”*  *“In addition, daily input of palliative care expertise during the oncology ward rounds meant that patients who would not have been referred in the usual inpatient consult service could now access specialist advice by the palliative care team in the co-rounding model.*  *“Sometimes we [oncologists] don’t refer patients to palliative medicine because we think the symptoms are fairly well controlled, but since the palliative doctor or nurse is rounding with us, we can get feedback on other things, for example resources [after hospital discharge] that may be available to patients... these [palliative care advice given during ward rounds] may make a small but meaningful impact on patient outcomes. (Oncology doctor 2)”* |
| --- |

| **CMO #2:**  **Context**: PC specialists who are integrated in the primary care oncology team using a co-rounding model  **Mechanism**: If PC specialists are integrated team members, then access to specialized PC is improved and management of broader patient problems is facilitated which contributes to a holistic approach in (cancer) care and rapport building. This ultimately leads to…  **Outcome**: improved patient outcomes/quality of patient care |
| --- |

| **Data excerpt 3:**  *“Early introduction to palliative care, as a result of a visible collaboration between teams, was perceived to have helped rapport building with patients. The oncology doctors spoke of how the SPARK co-rounding model allowed the palliative care doctor and nurse have a bit of a relationship with patients and their families early on, “when they are relatively well instead of being interested only when they become very sick” (Oncology doctor 2). This view was echoed by the palliative care nurse who felt that in the co-rounding model, she could identify “patients who need palliative care and start to build rapport with patients and family members early.” This in turn enabled her to address patients’ problems before they become insurmountable.”* |
| --- |

| **CMO #3:**  **Context**: If a visible collaboration between teams is established,  **Mechanism**: then early relationship-building occurs between PC specialists and patients and their families.  **Outcome**: This in turn creates the possibility to address patients’ problems earlier contributing |
| --- |

# Medium Relevance – High Rigour

## 14. Artioli et al., [23]

| **Context + intervention :**  The evaluation of a PC training program directed to generalist health professionals.  The training program lasted 4 hours and was given by PC specialist to health professionals of hospital departments (radiotherapy service, geriatrics service, and nephrology/dialysis service). The training focused on the vision of PC by the World Health Organization (WHO), the purpose of a PC unit in the hospital and the sharing of PC needs in hospital wards.  In agreement with Schenker and Arnold, to improve PC for patients with chronic illness, specific training to develop I-level PC skills in HPs to improve the quality of care in hospital was used. |
| --- |

| **Data excerpt :**  ***Knowledge improvement:***  *“Improvement of patient QoL”, which evidenced a statistically significant difference (p-value = < 0.001) between pre- and post-training, with an approximately three-fold increase. Two other domains also showed significant enrichment with training: D14 “Early applicability in illness path” (p-value = < 0.001) and D08 “Addressing patient and family needs” (p-value = < 0.001). The domains that concern addressing the patient’s holistic conception and taking charge of all its dimensions (D06 “Psychosocial aspects of patient care” and D07 “Spiritual aspects of patient care”) had significantly higher representation after the training intervention (p-value = < 0.001). These results also confirm that these domains had been properly learned during the course. These domains were followed in representation by D05 “Treatment of physical symptoms” (p-value = 0.001), which doubled after training; D12 “Team approach in addressing needs” (p-value = 0.006); and D10, referring to extending care to patients’ families, “Helping family to cope with their bereavement” (p-value = 0.016).*  *… The significant differences between pre- and post-intervention were suggestive of changes in the understanding of the topics, increased awareness or acquisition of new notions.*  *… to improve PC for patients with chronic illness, we used specific training to develop I-level PC skills in HPs to improve the quality of care in hospital.”*  ***Organization of PC:***  *“Once the hospital professionals received training on PC, they became aware of the broader picture and organization of PC, the paths for pursuing its integration within daily hospital activity and its implementation outside hospital structures. Additionally, having a better comprehension of the roles and profiles of the professionals working in this service allowed the other hospital professionals to be more attentive and sensitive in facilitating palliative doctor consultancies.*  *Having clarified that PC needs can actually be met by the PC specialist once a PC plan is activated led the participants to feel a sense of synergy with colleagues that seemed to facilitate inter-professional relations. The presence of the palliative specialist helped trainees to achieve a new perspective.*  *“Now, after the training, there is a lot more awareness (on PC), and we can work in line with the medical staff,…”*  *The participants’ understanding of the organization within the PCU seemed to have facilitated the involvement of the palliative doctor’s consultancies within the care. Participants became aware of the practical possibility of activating PC and showed the need for greater integration of PC specialists within the examined operating units (OUs).”* |
| --- |

| **CMO #1:**  **Context :** Healthcare workers from radiotherapy, geriatrics, nephrology and dialysis wards who received a 4 hour training to develop palliative care skills.  **Mechanism :** developed a better understanding of palliative care as defined by the WHO. This leads to   1. specific awareness of the organization of palliative care within their hospital 2. a better comprehension of the specific roles which allows hospitalists to be more attentive and sensitive in facilitating palliative doctor consultancies   This leads to a sense of synergy which ultimately causes  **Outcome :** more involvement of palliative care specialists |
| --- |

| **Data excerpt 2:**  *“… The significant differences between pre- and post-intervention were suggestive of changes in the understanding of the topics, increased awareness or acquisition of new notions.*  *The participants discovered the importance of EoL treatments, as they reported a higher sensitivity and attention to the patient at the EoL after training. Nurses recognized that physicians, after the training, were more likely to involve palliative doctors, when before they felt more embarrassed. All the HPs gained a clear understanding of the area of intervention of PC, namely, the response to the needs of the person, even when they could no longer manage the treatment of the disease.*  *“Surely our doctors have become confident enough to call the palliative doctor, and this alone is an important step ahead”.* |
| --- |

| **CMO #2:**  **Context:** Healthcare workers from radiotherapy, geriatrics, nephrology and dialysis wards who received a 4 hour training to develop palliative care skills.  **Mechanism** developed a better understanding of palliative care as defined by the WHO. This leads to a higher sensitivity for end of life care  **Outcome :** therefore leads to more involvement of palliative care physicians. |
| --- |

## 15. Braus et al., [30]

| **Context + Intervention:**  Participants included patients with high risk of mortality, morbidity or unmet PC needs admitted to a 24-bed **ICU** at a 566-bed academic medical center.  Investigators screened the ICU admissions on weekdays to identify patients with 1 or more prespecified **clinical trigger criteria** indicating their high risk of mortality, morbidity or unmet PC needs. In the usual care phase for recruiting control patients, in the intervention phase to receive the intervention.  The intervention was a **novel, proactive intervention** aimed at improving ICU physician team’s attention to PC need. A PC clinician was relocated from the PCU to the ICU and when a patient was screened positively for 1 or more of the trigger criteria the **PC clinician interacted with ICU physicians on daily rounds**. On subsequent days, the PC clinician would **make suggestions** about addressing PC needs, including **recommending interdisciplinary family meetings when appropriate.**  The PC clinician was:  - on most days a **PC clinical nurse specialist** with many years of experience in both hospice and PC practice.  - on other days (less than 10%), a PC medicine fellow or faculty member. |
| --- |

| **Data excerpt 1:**  *“Every weekday morning the investigators (WJE or TL) informed the PC clinician about patients meeting trigger criteria. The PC clinician then reviewed the electronic medical record (EMR) of each of these patients and participated in interdisciplinary morning bedside ICU rounds with the critical care medicine team. On the first day that a patient was identified, the PC clinician informed the medical team that the patient met one or more of the trigger criteria, and which trigger criteria or criterion the patient met. On subsequent days, the PC clinician would make suggestions about addressing PC needs, as appropriate, including recommending that interdisciplinary family meetings be held in a timely fashion.”*  *“Only 35 % of the subjects in the usual care period had documentation of an interdisciplinary family meeting during their ICU stay, and for those patients for whom such a meeting was documented, only 35 % of these meetings took place within the first 3 days in the ICU (Table 3). In adjusted analysis, the intervention was associated with a 63 % higher likelihood of a documented family meeting occurring during the ICU stay [RR 1.63, 95 % confidence interval (CI) 1.14–2.07, p = 0.01]. The adjusted time between ICU admission and the occurrence of a family meeting in the ICU was 41 % shorter in the intervention period (95 % CI 52–28 % shorter, p<0.001).*  *“Total family satisfaction, satisfaction with the care their loved one received, and satisfaction with decision-making during the ICU stay were similar between the intervention and usual care groups (83.7 in usual care versus 81.7 in the intervention, p = 0.52).”*  *“straightforward trigger criteria leading to a simple intervention—a PC clinical nurse specialist interacting with the critical care team on rounds—was associated with earlier and more frequent interdisciplinary family meetings in the ICU.”*  *“It is not known what aspect of the intervention may have been effective. For example, simply identifying high-risk patients through the use of trigger criteria and informing the ICU physicians about this identification may account for the findings observed in this study, independent of the presence of a clinical nurse specialist on rounds. Indeed, awareness of the ongoing study and of being observed may alone have altered ICU physicians’ behavior regarding family meetings for high-risk patients. If this is the case, an even simpler and less costly intervention might have demonstrated similar effects. On the other hand, it may be that the presence of a PC specialist on morning rounds prompted greater consideration of patients’ and families’ PC needs and nudged critical care providers towards meeting those needs, beyond what the trigger criteria alone could have accomplished.”* |
| --- |

| **CMO #1:**  **Context**: Screening for unmet PC needs (with use of trigger criteria) + PC specialist involved in daily bedside rounds  **Mechanism**: this intends to prompt the critical care team to consider patient’s and families’ PC needs.  **Outcome**: leading to earlier and more frequent family meetings + similar family’s satisfaction |
| --- |

## 16. Courtright et al., [49]

| **Context + Intervention:**  Participants included hospitalists from seven hospitals (two teaching and five community) across seven states. All study hospitals had an established palliative care program ranging in duration from 5 to 18 years.  A telephone interview was conducted with the participants to understand their decision-making about PCCs for patients with dementia and to describe **factors that influence their likelihood of referral**. |
| --- |

| **Data excerpt 1:**  *“Hospitalists generally acknowledged the family’s burden of caregiving and decision-making for a loved one with dementia. When they perceived that necessary medical decision-making was impeded by a family’s lack of understanding of the disease trajectory or intra-family disagreement about goals of care, most were inclined to involve palliative care. However, hospitalists reported they were less likely to consult palliative care for patients with limited family presence or involvement in caregiving. Similarly, most were unwilling to consult palliative care if they perceived that families would be resistant to it.”*  *“Hospitalists also perceived the family as a barrier to referral. Specifically, hospitalists did not feel comfortable consulting palliative care if they sensed the family would be unwilling to have conversations about serious illness.”* |
| --- |

| **CMO #1:**  **Context**: : (1) If hospitalists sense a lack of understanding about the disease trajectory or intra-family disagreements  (2) if family members were less present or involved in the patient care or the hospitalist had a sense of resentment for PC among the relatives  **Mechanism**: (1) then they feel more inclined to consult the PCT, thus facilitating collaboration.  (2) then they were less inclined to consult the PCT, thus hindering collaboration.  **Outcome**: / |
| --- |

| **Data excerpt 2:**  *“Relatedly, some hospitalists indicated that their palliative care team often seemed busy, making them more inclined to attempt to address patients’ and families’ needs on their own when they might otherwise have consulted first. Others commented that their palliative care teams always responded in a timely manner, thereby increasing hospitalists’ inclination to request a consult.”* |
| --- |

| **CMO #2:**  **Context**: (1) If the PCT respond to consultation requests in a timely manner,  (2) If the PCT seems busy to the hospitalists,  **Mechanism**:   1. then hospitalists are more inclined to contact them 2. then they are more inclined to address patients’ and families’ needs on their own   **Outcome**: / |
| --- |

| **Data excerpt 3:**  *“Many hospitalists opined that there was not enough time to accomplish their daily patient care tasks and also have a presumed lengthy goals of care conversation with families of patients with dementia. Additionally, several hospitalists commented that palliative care specialists had superior skills to navigate the challenging prognostic and goals of care discussions with families. For example, when hospitalists disagreed with a family’s preference for aggressive care, they felt palliative care consultation was appropriate to facilitate communication. Even among the few hospitalists who expressed high confidence in their abilities to address the palliative care needs of their patients and families, insufficient time remained a major reason to consult palliative care.”*  *“Hospitalists’ limited time was a strong driver of consultation. Confidence, or lack thereof, in serious illness communication skills also impacted the consultation decision. Similar to our findings, a national survey revealed that most hospitalists feel confident in their ability to discuss goals of care or prognosis but less so for improving understanding of disease or resolving conflict.20 However, a recent simulation study suggests that physician’s self-assessment of communication skills may not correlate with adherence to best practices in family meetings.21 More research is needed to understand the impact of hospitalists’ confidence in palliative care skills on the quality of care and patient outcomes”* |
| --- |

| **CMO #3:**  **Context**: If hospitalists feel they do not have enough time or a lack of (confidence in their) PC skills,  **Mechanism**: then they have a bigger need to consult the PCT  **Outcome**: / |
| --- |

## 17. Economos et al., [35]

| **Context + Intervention:**  Participants included 18 physicians (medical oncologists and medical specialists working in oncology) from 2 tertiarty university hospitals and one regional cancer center in France with access to a specialist PCT.  Physicians who were not clinically involved (researchers) or paediatric oncologists, haematologists, surgical oncologists or radiotherapists were excluded. Access to PC is available through a mobile PCT in the institution.  Semi-structured, face-to-face, individual interviews were held to explore physicians’ perceptions of PC and their opinion on how to improve collaboration between clinicians in PC and oncology. |
| --- |

| **Data excerpt 1:**  *“They [oncologists] highlighted the importance of a distressing symptom, especially refractory pain, which was used as a trigger for referring the patient to palliative care. The occurrence of a new symptom was often considered as a turning point in the disease trajectory reflecting a worsening of the clinical status and a short-term prognosis.”*  *“Most of the participants acknowledged that they referred patients to palliative care for their greater experience and specialised skills in managing complex burdensome symptoms.*  *We are glad to have the support from specialized teams when we have little knowledge in the field […] we need overspecialization. (I2)”*  *“Some participants referred patients to palliative care when there was no new specific treatment, or when they felt overwhelmed by the situation and were faced with limited specific treatments. For oncologists, the aim was then to allow the continuity of care.*  *When I start chemotherapies with an uncertain effectiveness […] when I am limited in my prescriptions, because, beyond morphine I will not know what to do […] at this time I feel that things are going to end […] and I refer him (the patient) to palliative care. (I10)”* |
| --- |

| **CMO #1:**  **Context**: If oncologists are confronted with distressing symptoms (reflecting the worsening of the clinical status and short-term prognosis),  **Mechanism**: they feel overwhelmed and need support of the PCT for their greater experience and specialized skills,  **Outcome**: therefore more easily refer their patients to the PCT |
| --- |

| **Data excerpt 2:**  *Psychosocial support*  *“This support was also appreciated in supporting difficult end-of-life decision-making Participants described the need of having an ‘outside look’ when discussing the limitations of life-sustaining therapeutics, especially for younger oncologists. To allow this, participants argued that an absence of judgement and real dialogues were key points in the collaboration.*  *We are not alone to take the decision and to say “ok, we stop everything and we start the Midazolam” It allows (us) to have an outside look to take difficult decisions. (I15).*  *At these times, we are glad and relieved that someone else stands by our side (I13).”*  *“Finally, some practitioners also used palliative care as a support to relieve the burden that caregivers experience when facing distressing situations.”*  *“Palliative care referral was perceived as helping in addressing communicational and decision-making issues, as well as in helping to manage refractory symptoms.”* |
| --- |

| **CMO #2:**  **Context**: If PC specialist uses an absence of judgement approach and real dialogues  **Mechanism**: then ward staffs feel supported in taking difficult decisions  **Outcome**: a burden relief among primary caregivers when facing distressing situations |
| --- |

| **Data excerpt 3:**  *“This support was also appreciated in supporting difficult end-of-life decision-making. Participants described the need of having an ‘outside look’ when discussing the limitations of life-sustaining therapeutics, especially for younger oncologists.”*  *“Besides a lack of dedicated training, the short medical experience results in fewer experiences in facing and managing palliative care. This explains that these physicians are less likely to engage in managing palliative care by themselves and more prone to refer to palliative care.”* |
| --- |

| **CMO #3:**  **Context**: Oncologists with shorter work experience  **Mechanism**: feel the need of having an outside look  **Outcome**: / |
| --- |

## 18. Morikawa et al., [47]

| **Context + Intervention:**  Participants included hematologists and PC specialists who are board certified in their specialty and work for a hospital providing hematology medicine services or a hospital-based PCT. All participants had been working in the areas of expertise for a minimum of 5 years. Included hospitals were one university hospital, one general hospital and one cancer center.  In-depth, face-to-face, semi-structured interviews were held with the participants about the roles and expectations of the PCT, and the barriers to collaboration between hematologists and PCTs on relapse or refractory leukemia and malignant lymphoma patients’ care.  No information on PCC process provided |
| --- |

| **Data excerpt1:**  *“Both hematologists and palliative care specialists mentioned that hematologists often do not require much intervention by the HPCT. Five hematologists indicated H-1 (not expecting treatment and care in a way preferred by hematologists) and 7 palliative care specialists indicated P-1 (hematologists take pride in their symptom management skills) or P-2 (hematologists tend to only consider treatment of the primary disease).”*  *“Table 3: Barriers for collaboration 1. Don’t feel the need to refer*  *- Not expecting treatment and care in a way preferred by hematologists*  *- Hematologists take pride in their symptom management skills*  *- Hematologists tend to only consider treatment for the primary disease*  *- Hematologists tend to carry patient’s concerns by themselves*  *- If support cannot be provided as desired by the hematologist, referrals will stop*  *- Patients do not fully understand the role of the HPCT and thus do not ask for intervention”* |
| --- |

| **CMO #1:**  **Context**: If hematologists expect treatment and care by the PCT not to be in their preferred way OR  If hematologists don’t receive support as desired  **Mechanism**: Then they do not feel the need to refer their patients to the PCT  **Outcome**: / |
| --- |

| **Data excerpt 2:**  *“Both hematologists and palliative care specialists mentioned that hematologists often do not require much intervention by the HPCT. Five hematologists indicated H-1 (not expecting treatment and care in a way preferred by hematologists) and 7 palliative care specialists indicated P-1 (hematologists take pride in their symptom management skills) or P-2 (hematologists tend to only consider treatment of the primary disease).”*  *“Table 3: Barriers for collaboration 1. Don’t feel the need to refer*  *- Not expecting treatment and care in a way preferred by hematologists*  *- Hematologists take pride in their symptom management skills*  *- Hematologists tend to only consider treatment for the primary disease*  *- Hematologists tend to carry patient’s concerns by themselves*  *- If support cannot be provided as desired by the hematologist, referrals will stop*  *- Patients do not fully understand the role of the HPCT and thus do not ask for intervention”*  *“Both hematologists and palliative care specialists identified the timing of referral as a barrier to collaboration between hematologists and the HPCT. More than half of the hematologists interviewed indicated H-3 (the treatment of primary disease is given priority over palliative care from the HPCT until the last stage). Similarly, more than half of the palliative care specialists indicated P-6 (hematologists and patients resist HPCT input when they hope to be cured)”*  *“In this study, hematologists and palliative care specialists felt it was difficult to refer patients to the HPCT during intent-to-cure treatment.”* |
| --- |

| **CMO #2:**  **Context**: (1) If hematologists take pride in their own symptom management skills OR  If hematologists take ownership over the patient and the treatment OR If hematologists only consider treatment as an option for the primary disease/give priority to primary disease treatment/focus on intent-to cure treatment  (2) If hematologists and patients hope for a cure,  **Mechanism**: (1) Then they do not feel the need to refer  (2) Then resistance occurs towards PCT input.  **Outcome**: / |
| --- |

| **Data excerpt 3:**  *“Five hematologists indicated H-5 (the HPCT does* *not aggressively approach hematologists with input).”*  *“Table 3: Barriers to collaboration*  *3. Lack of an aggressive approach by the HPCT*  *- The HPCT does not aggressively approach hematologists with input*  *- Not trying to approach hematology staff actively owing to insufficient human resource”*  *“7. Others: Furthermore, 4 hematologists indicated H-12 (concern about the lack of HPCT manpower, so hesitation to refer) and 7 palliative care specialists indicated P-16 (difficult to collaborate with hematologists who have a lack of knowledge of palliative care).”* |
| --- |

| **CMO #3:**  **Context**: If there is a lack of manpower in the PCT/ If there is insufficient human resources within the PCT,  **Mechanism**:   1. then hematologists are hesitant to refer. 2. then they do not actively approach hospital staff   **Outcome**: |
| --- |

| **Data excerpt 4:**  *“4. Negative image of the HPCT: A few hematologists identified the image of the HPCT as a barrier to HPCT referral by indicating H-8 (concern that patients will associate referral to the HPCT with imminent death).”*  *“Table 3: Barriers to collaboration*  *4. Negative image of the HPCT*  *- Concern that patients will associate referral to the HPCT with an imminent death.*  *- Hesitation to refer when hematologists would like the HPCT to just listen to patients’ distress.”* |
| --- |

| **CMO #4:**  **Context**: If hematologists are concerned about patient’s negative image of the PCT,  **Mechanism**: then they are hesitant for referral.  **Outcome**: / |
| --- |

| **Data excerpt 5:**  *“6. Lack of communication: A lack of communication was mentioned more often by hematologists than by palliative care specialists. Seven hematologists indicated H-11 (lack of discussion with the HPCT about ways of interacting with patients).”*  *“Table 3: Barriers for collaboration*  *6. Lack of communication:*  *- Lack of discussion with the HPCT about interacting with patients. (7)*  *- Lack of discussion with hematologist owing to their busy schedule (1)*  *- Difficult to act when hematologists do not respond to the HPCT’s suggestions (1)”*  *“Furthermore, although more than half of the participating hematologists also mentioned the lack of communication with the HPCT about ways to interact with patients, only one palliative care specialist did otherwise, suggesting that palliative care specialists consciously communicated with hematologists. On the other hand, hematologists recognized the need to discuss with the HPCT about patients’ condition but felt there was not enough communication.”*  *“These results suggest that it is necessary for a good collaboration that hematologists improve future discussion with the HPCT on patients’ condition and that the hematology team staffs communicate with the HPCT to compensate for the lack of information. Moreover, palliative care specialists indicated the lack of hematologists’ knowledge of palliative care, which further suggests the importance of communication to overcome the differences from each other.”* |
| --- |

| **CMO #5:**  **Context**: If hematologists have a busy schedule or do not respond to PCT’s suggestions,  **Mechanism**: then a lack of communication occurs (*which may contribute to a lack of knowledge about PC and lack of information*), which in turn hinders collaboration  **Outcome**: / |
| --- |

| **Data excerpt 6:**  *“7. Others: Furthermore, 4 hematologists indicated H-12 (concern about the lack of HPCT manpower, so hesitation to refer) and 7 palliative care specialists indicated P-16 (difficult to collaborate with hematologists who have a lack of knowledge of palliative care).”* |
| --- |

| **CMO #6:**  **Context**: If hematologists have a lack of PC knowledge  **Mechanism**: then collaboration is perceived as difficult by PCT  **Outcome**: / |
| --- |

## 19. Pan et al., [53]

| **Context + Intervention:**  Participants included 284 registered nurses working on hospital wards or ICUs in a medical center in Taiwan. They filled out the KAP-PCCSI questionnaire to evaluate their knowledge, attitude and practice regarding the PCC service. At the same time path modelling was carried out to examine causal patterns among variables.  The PCC service is described as a model of PC that has provided holistic EOL care for terminally ill patients in acute ward settings since 2005. The PCT is made up of the following members: doctors, nurses, psychologists, social workers, pharmacists, and spiritual care workers. They collaborate with primary health professionals to mainly provide advice, support, and guidance regarding PC. |
| --- |

| **Data excerpt 1:**  *“The K-PCCSI was used to measure the level of understanding regarding the concept, purpose, significance, consultation process, and referral of patients. It contains 15 items; each item was rated on a 1–5 score with 1 being “no understanding” and 5 being “full understanding.” The total score ranges from 15 to 75,* ***with a higher score indicating better knowledge in PCCS.”***  *“the significant predictors of the K-PCCS among nurses were ward, participation in education related to palliative care, and frequency of contact with PCCS, after adjustment for potential confounders. Nurses from medical wards, surgical wards, and ICU had lower mean scores for the K-PCCS than those from hematology and oncology wards by 7.2 points [95% CI (−10.2, −4.1)* *p<0.001], 5.0 points [95% CI (-8.1, -1.7), p<0.01], and 4.3 points [95% CI (-8.2, -0.3), p<0.05]. Nurses who have participated in education related to palliative care had a higher mean score for the K-PCCS than those who had never participated in relevant education by 2.0 points [95% CI (0.1, 3.9), p<0.05]. Compared with nurses who have never had contact with PCCS, those with 1–4 times and 5 or more times had a higher mean score for the K-PCCS by 4.5 points [95% CI (1.9, 7.1), p<0.001].”* |
| --- |

| **CMO #1:**  Context:  RNs who work in an oncology or hematological ward  RNs who had previous education in PC  RNs who have a frequent contact with their PCT *(1–4 times and 5 or more times)*  Mechanism: Develop a greater understanding about the consultation process and why to refer their patients.  Outcome: / |
| --- |

| **Data excerpt 2:**  *“The A-PCCSI is composed of 10 items measuring attitudes toward PCCS team intervention and improving the quality of life of terminally ill patients. Items 9 and 10 were negatively worded. All items are rated on a 1–5 score with 1 being “strongly disagree,” and 5 being “strongly agree.” The total score ranges from 10 to 50, with a* ***higher score indicating a more favorable attitude toward PCCS****.”*  *“Ward, experience of the death of friends or relatives, and frequency of contact with PCCS were important predictors for the A-PCCS after adjustment for potential confounders. Nurses in the obstetrics and gynecology wards, medical wards, surgical wards, and ICU had a lower mean score in the A-PCCS than those in the hematology and oncology wards by 3.5 points [95% CI (−6.5, −0.4), p<0.05], 4.1 points [95% CI (−6.3, −1.9), p<0.001],* *3.6 points [95% CI (−5.9, −1.3, p<0.01],* *and 4.5 points [95% CI (−7.3, −1.6), p<0.01].* *Nurses who have experienced the death of friends of family had higher mean scores than those who have not by 1.9 points [95% CI (0.1, 3.7), p<0.05].* *Compared with the nurses who have never had contact with PCCS, those with 1–4 times and 5 or more times showed an increased mean score for the A-PCCS by 1.9 points [95% CI (0, 3.8), p<0.05] and 3.2 points [95% CI (1.0, 5.4), p<0.01].”* |
| --- |

| **CMO #2:**  **Context**:  RNs who work in an oncology or hematology ward  RNs who have a frequent contact with their PCT (*1–4 times and 5 or more times)*  **Mechanism**: develop a better attitude regarding the PCT and their interventions.  **Outcome**: / |
| --- |

| **Data excerpt 3:**  *“The P-PCCSI is composed of 10 items measuring the nurse’s ability to determine whether a patient meets the admission criteria, clearly explain and provide relevant information regarding PCCS to patients and relatives, discuss with the PCCS team regarding the patient’s condition or medical procedures, proactive learning of comfort care and terminal care techniques provided by the PCCS team, proactive communication with PCCS practitioners, and proactive sharing of personal thoughts. The items were rated on a 1–5 score with 1 being “never,” and 5 being “always.” The total score ranges from 10 to 50,* ***with a higher score indicating more favorable practices regarding PCCS.”***  *“Participation in education related to palliative care and frequency of contact with PCCS were significant predictors of the P-PCCS among nurses. Nurses who have participated in education related to palliative care had a higher mean score for the P-PCCS than those who have never participated in relevant education by 1.7 points [95% CI (−0.04, 3.4), p<0.05].* *Compared with the nurses who have never had contact with PCCS, those with 5 or more times had a higher mean score for the P-PCCS by 6.0 points [95% CI (3.3, 8.7), p<0.001].”* |
| --- |

| **CMO #3:**  **Context**: If RNs had education in PC in the past or if they have a frequent contact *(5 or more times)* with their PCT, then  **Mechanism**:  - their abilities to have discussions with the PCT increases  - they are more able to learn proactively from the PCT  - they have a better proactive communication with the PCT  - they are more able to proactively share their personal thoughts with the PCT  **Outcome**: / |
| --- |

| **Data excerpt 4:**  *The path modeling demonstrated that having a master’s degree significantly affected practice through knowledge and attitude compared to colleagues with an undergraduate degree (Coefficients = 0.169, p = 0.004). The model also showed that educational level, marital status, ward, and the frequency of contact with PCCS directly affected practice….. Working in a medical ward (Coefficients = −0.324, p < 0.001), surgical ward (Coefficients = −0.232, p < 0.001), or ICU ward (Coefficients = −0.293, p < 0.001) were also significantly negative in relation to practice compared to working in a hematology or oncology ward. In addition, comparison of nurses who had never contacted PCCS, with those who had contacted PCCS for 1–4 times showed a positive* *significant correlation with practice (Coefficients = 0.220, p < 0.001).”*  *“The results of our path analysis indicated that bachelor degree or above, being single, working in a haematology and oncology ward, and frequency of contact with PCCS had positive direct effects on liaison with PCCS. Having a master’s degree had a positive, although indirect effect, on nurse practice through the influence on knowledge and attitude. This implies that nursing staff with a higher educational level would have more knowledge about PCCS, which indirectly resulted in more favorable attitude and better practice toward PCCS.*  *“Nurses working in a haematology and oncology ward were influenced in their practice more by PCCS than those work worked in medical and surgical wards and the ICU. It is likely that nurses working in a haematology and oncology ward had more opportunities to experience how to take care of terminal patients in their daily practice, which may help them to refer patients to PCCS specialists. The frequency of contact with the PCCS also positively influenced practice and interaction with the PCCS. In our clinical practice, the primary nurse who contacted the PCCS team members could discuss the terminal patients’ condition with them directly.* *Non-specialist ward nurses reported a lack of training in relation to cancer care which could impede the ability to provide high quality nursing care to patients with cancer diagnoses. In addition, emotional support and communication ability with patients and their families could also distress non-specialist nurses. Therefore, it is important that the practice of contacting PCCS team members to learn and develop clinical ability is encouraged.”* |
| --- |

## 20. Sarradon-Eck et al., [48]

| **Context + Intervention:**  Participants included 13 oncologists working in a comprehensive cancer center and 19 PC specialists working in 10 hospitals all over France.  According to the latest data, France (67 million inhabitants) has 157 Acute PCUs, 5057 dedicated PC beds (distributed among 835 institutions), 107 PC networks (ensuring continuity between the hospital and home care provided by primary care professionals), and 428 PCTs. All French Comprehensive Cancer Centers and University Hospitals have one PCT as well as dedicated PC beds. The aim of PCTs is to support hospitals and home care teams and to disseminate information about the palliative culture. PCTs have a consulting role and advise specialist units only at their request.  To implement early PC at French hospitals and promote the ‘‘integrated care model’’, that is, providing palliative care as part of oncological care, the EPIC trial—a randomized multicenter clinical trial – was launched in France in 2017. The oncologists (n = 13) and most of the PCSs (n = 11) interviewed, worked at four of the Comprehensive Cancer Centers involved in the EPIC trial. To obtain various viewpoints about referral to PC specialists, interviews were also conducted with five PC specialists working at three other Comprehensive Cancer Centers and University hospitals involved in the EPIC trial and three PC specialists working at University Hospitals not involved.  All participants participated in semi-structured, individual, face-to-face interviews to unravel social and cultural factors underlying oncologists’ attitudes to early referral and working together with PC specialists in French hospital settings. These interviews were conducted before the EPIC trial was launched. |
| --- |

| **Data excerpt 1:**  *“How physicians’ perception of patients’ understanding of palliative care affects their referral practices*  *Both oncologists and PCSs stated that palliative care is thought by patients and their families to mean imminent death, hospice care, and end-of-life care. These results show how physicians’ perception of patients’ understanding of palliative care impacts the process of communication and patients’ referral to palliative care services…..*  *Because of the patients’ perception of PC as end-of-life care, the oncologists interviewed feared that announcing referral to a PCS might have negative effects on patients by causing a loss of compliance with their palliative chemotherapy or a loss of hope and feelings of distress. According to the respondents, that is why some oncologists are reluctant to refer patients for EPC:*  *The Head of a thoracic oncotherapy ward just told me, ‘‘If I inform people that they have small cell cancer or metastases, and then go on to say I am going to call in the mobile palliative care team, they are going to throw themselves under a train!’’ (P12, a PCS).*  *In the physicians’ discourse, referral to a PCS appears to be equivalent to announcing a poor prognosis:*  *EPC means referring patients at an early stage in their disease. But we are already trying to tell them they have cancer, and I feel it’s a bit much to have to also say, ‘‘We are going to transfer you to palliative care because there is nothing else we can do’’ because palliative care has strong connotations. And while I am saying that, I realize that since we are talking about a serious disease, we should really go on to mention the prognosis straight away. but to me, that seems to be a difficult thing to do . Announcing at a single consultation ‘‘you have cancer, and the cancer is at a very advanced stage’’. amounts to announcing a lot of things all at the same time (P4, an oncologist)*  *This situation may be a barrier to patients’ access to EPC, even in the context of the EPIC trial, not only because it is distressing and painful for both patients and oncologists but also because announcing a short lifetime scenario also seems to run counter to the medical culture focusing on curing patients, especially at CCCs (which are called ‘‘Cancer-fighting Centres’’ in France)”*  *“The first definition of palliative care, which was put forward by Therese Vanier (‘‘When there is nothing more that can be done, everything can still be done’’), is still being frequently quoted in French palliative care training courses and handbooks. Although this definition was intended to save end-of-life patients from being abandoned, it reinforces the idea that palliative care is last-resort care, and made it difficult for some of the oncologists interviewed to regard it as being complementary to active cancer care.”* |
| --- |

| **CMO #1:**  **Context**: If oncologists have the perceptions patients do not understand what PC is  **Mechanism**: then they fear to announce a PC referral to these patients because they fear to cause a loss of compliance with therapy or loss of hope among their patients This fear then leads to a reluctance to refer patients to PC specialists.  **Outcome**: / |
| --- |

| **Data excerpt 2:**  *“EPC: a poorly understood concept*  *Some PCSs thought the value of palliative care is not properly recognized:*  *They (the oncologists) accept us .they feel it’s useful but they don’t think it’s proper medicine. Proper medicine is cancerology, the medicine which treats people. When we* *have no choice, we turn to palliative care but it’s not in the same league (P23, a PCS).*  *In view of the above statements, one can say that PCSs are regarded as service providers rather than delivering care as part of integrated care projects:*  *We have to serve some purpose, in other words, they use us because we help to solve their problems, but (.) they do not regard us as being an integral part of integrated care (.) We help them to get over a difficult patch or to sort out tricky situations.(.) but as long as this is not an obligatory procedure, our role is difficult to. I mean we are still working in parallel, as stand-bys (P19, a PCS).*  *Finally, some of the oncologists interviewed depreciated palliative care practices, describing them as not always evidence-based practices. They objected that palliative care is not properly defined or only ‘‘in very vague terms’’, ‘‘not terribly exactly’’: ‘‘I have never been able to understand what this so-called whole person care means’’. Others mentioned criticisms made by colleagues: that palliative care is ‘‘just hot air’’ and consists of ‘‘holding patients’ hands until they die’’. Some of the oncologists interviewed stated that EPC is the same thing as supportive care. From their point of view, oncologists are specialized in treating cancer and PCSs in treating symptoms. That is why they found it difficult to refer asymptomatic patients to a PCS at an early stage.*  *…*  *The latter excerpt highlights the fact that in France, medical oncologists often have to provide both supportive and palliative care, either because they are working at private clinics with no PCSs or because some of them (especially the oldest ones) feel they are responsible for providing cancer patients with all the care they need, including their end-of-life care. PCSs find it difficult to work together with these practitioners, especially since the specialization of PCSs is not unanimously recognized at French hospitals:*  *It’s funny because when doctors ask a cardiologist for his opinion, he will say ‘‘do this and do that’’: nobody will question his advice (.) and his prescriptions will be applied. But when patients need palliative care, especially end-of-life care, everybody thinks they have been properly trained for this. They tell themselves ‘‘anyone can handle the end of life, anyone can handle pain’’ (.). And we often get the impression that they think our intervention means they lack ability (.) We can provide expertise which can be helpful or even sometimes support the medical teams, but this assistance is underestimated and it is not yet recognized as a specialty (P20, a PCS).*  *This lack of legitimacy can generate conflicts over professional boundaries, which block recourse to EPC.”*  *“Oncologists’ picture of PCSs’ work is often restricted to managing the physical symptoms and providing psychosocial support”* |
| --- |

| **CMO #2:**  **Context**: If PC specialists (and its specialization) do not feel recognized by oncologists  **Mechanism**: then PCT specialists find it difficult to work together with them  **Outcome**: / |
| --- |

## 21. Van der Stap 1 et al., [22]

| **Context + Intervention:**  This study involved focus groups with 51 participants consisting of stakeholders involved in PC symptom management, being:  - 6 patient representatives (informal caregivers of deceased patients)  - 12 community nurses  - 8 hospital nurses  - 8 general practitioners  - 9 hospital physicians  - 8 PC specialists  By conducting these interviews researchers wanted to obtain a broad range of views and experiences of stakeholders with diverse backgrounds who are usually involved in PC symptom management about their views on facilitators and barriers for multidimensional symptom management.  Clinicians were considered PC specialists if they had completed 1 of the 3 dedicated Dutch PC training programs for nurses or physicians and/or were a member of a PCT. |
| --- |

| **Data excerpt 1:**  *“Theme 3: Multidisciplinary collaboration*  *Generalist and specialist clinicians felt that collaboration of generalist clinicians with other disciplines, such as specialist palliative care teams, psychologists, spiritual caregivers, and social workers, facilitated multidimensional symptom management. Generalist community and hospital nurses mentioned scheduling multidisciplinary meetings with generalist and specialist clinicians as a solution for improving the clinical practice of generalist clinicians because it helps them to address all dimensions of palliative care. This was also the experience of palliative care specialists.”*  *“As of recently, we’ve been doing case discussions every 2 weeks with someone from the palliative care consultation team, according to the decision-making in palliative care method and palliative reasoning. We do this with a group of people and discuss the 4 dimensions, and then you often encounter things that you haven’t thought of before. Even though you’ve been taking care of the patient for 3 days, you still missed them. (Hospital nurse 2)”*  *“Our participants suggested that multidisciplinary team meetings are a potential solution for improving multidimensional symptom management….. Improving the awareness among generalist physicians about the importance of considering nonphysical problems during symptom management may allow palliative care specialists to discuss these problems during consultations.”* |
| --- |

| **CMO #1:**  **Context**: If PC specialists and ward staff have systematic case discussions,  **Mechanism**: then ward staff becomes more aware of all dimensions of PC.  **Outcome**: This in turn leads to an improvement in their clinical practice which facilitates patient’s multidimensional symptom management |
| --- |

| **Data excerpt 2:**  *“Palliative care specialists indicated that they feel that their multidimensional approach is not always appreciated by generalist physicians. This may prevent palliative care specialists from addressing nonphysical aspects of symptom burden and non-pharmacological interventions during consultations.”*  *“It’s dragging, pushing, luring, to make your consultation valuable. You often aren’t even allowed to ask additional questions. […] You aren’t always valued as a consultant in my experience. (Palliative care specialist 1)”*  *“Palliative care specialists indicated that their multidimensional approach is not always appreciated by generalist physicians, which prevents them from encouraging multidimensional symptom management.”* |
| --- |

| **CMO #2:**  **Context**: If the specialists feel their way of working (multidimensional approach) is not appreciated by generalist physicians  **Mechanism**: then it feels dragging, pushing and luring for the specialist to make a consultation valuable  **Outcome**: this prevents the specialists from encouraging multidimensional symptom management among ward staff |
| --- |

# Low Relevance - High Rigour

## 22. Mertens et al., [46]

| **Context + Intervention:**  Participants included 53 healthcare professionals with a diverse professional background: 10 physicians, 31 nurses, 4 psychologists, 4 social workers, 4 other (dietician or spiritual workers) which were all involved in PC and/or palliative patients’ transfers.  The research project involves all care settings: the home and nursing home care settings, as well as the hospital setting, which includes the hospital’s PCT and the PCU (with nine beds).  This was a focusgroup study exploring healthcare professionals’ experiences regarding the communicative aspects of inter-professional collaboration and the involvement of patient and family members within and across settings. |
| --- |

| **Data excerpt 1:**  *“Regarding the method, the absence of a shared electronic health record to exchange information between professionals within or across care settings was a perceived barrier.*  *“Overall, the availability of an efficient, digital platform, allowing for information sharing between professionals within and between settings, was deemed essential to overcome barriers of timely, relevant and complete information exchange”* |
| --- |

| **CMO #1:**  Context: If an efficient, shared, digital platform (EHR) is availiable  Mechanism: then this allows for information-sharing between professionals.  Outcome: timely, relevant, and complete information exchange |
| --- |

# High Relevance – Low rigour

## 23. Oertel et al., [37]

# Medium Relevance – Low rigour

## 24. Anandan et al., [31]

## 25. Beck et al., [50]

## 26. Berglund et al., [32]

## 27. Cannon et al., [33]

## 28. Gatta et al., [39]

## 29. Kawabata et al., [38]

## 30. Kennedy et al., [52]

## 31. Kyeremanteng et al., [51]

## 32. Zemplenyi 2 et al., [44]

|  |
| --- |
