# Supplementary material for: Interprofessional collaboration between hospital-based palliative care teams and hospital ward staff: A realist review
Source: PLoS One. 2025 Dec 19;20(12):e0338132. doi: 10.1371/journal.pone.0338132 (PMC12716714; doi:10.1371/journal.pone.0338132)
Supplement: S6 File — (DOCX) [file pone.0338132.s006.docx]

|  |
| --- |
| Supplementary file 6 |
| Recurrent mechanisms and corresponding CMO-configurations |

| Moons et al., 2025 |
| --- |

**Patient**

**PCT**

**OUTCOME**

**MECHANISM**

More familiarity with PCT [36]

More positive experiences with PCT [36]

Improving own clinical practice [34]

Requests of new PCC [33]

More involvement of PCT [35, 36, 37]

Facilitating multidimensional symptom management [34]

**CONTEXT**

**PCT**

**Ward staff**

Having high turnover of ward staff [24] (-)

Having systematic case discussions with PCT [34] (+)

Receiving PC training [35] (+)

Being visible/present [24, 33] (+)

Years of being active [36] (+)

Having a high ward staff turnover [1]

Having systematic case discussions with PCT [21]

Receiving PC training [14]

**Ward staff**

**Ward staff’s awareness for PC or PC services**

**OUTCOME**

**Ward staff**

Transfer of PC knowledge [44]

Feeling relieved because care and advice for palliative situations can be delegated to the PCT [44]

Increased utilization of PCC [38]

Increased timeliness of PCC [38]

Improved efficiency of PC [39]

Increased involvement of PCT [35]

Reduced symptom burden [44]

Better coping with disease [44]

Increased documented GoC [42]

Patient care in harmony with patients’ and families’ wishes [43]

Being included in treatment approach helping them to get about the situation [44]

Increased involvement

Increased utilization

Increased timeliness

Improved efficiency of PC

**CONTEXT**

**MECHANISM**

Receiving PC training [35]

**Ward staff**

**PCT**

Being present [24]

Being actively involved during rounds
[38, 39, 40, 41]

Making informal recommendations to ward staff [38]

Screening for unmet PC needs among hospitalized patients [40, 41]

Facilitating goals of care discussions between ward staff, relatives and patients [42, 43]

**PCT**

**Patient**

**Relatives**

**MECHANISM**

**OUTCOME**

Transfer of PC knowledge [44]

Feeling relieved because care and advice for palliative situations can be delegated to the PCT [44]

Reduced symptom burden [44]

Better coping with disease [44]

Being included in treatment approach helping them to get about the situation [44]

Earlier and more frequent meetings with relatives [40]

Satisfaction with care [40]

**PCT**

**CONTEXT**

**Ward staff**

Being actively involved during rounds [40]

Screening for unmet PC needs among hospitalized patients [40]

**Ward staff’s awareness for relatives’ PC needs**

**Patient**

**Relatives**

**MECHANISM**

**Ward staff**

**CONTEXT**

**OUTCOME**

Having a lack of time [44]

Having a lack of PC knowledge [44]

Being confronted with complexity of caring for patients with PC needs [44]

Being confronted with worsening of patient’s clinical status [45]

Asking for support from PCT [44]

Doing more referrals to PCT [45]

**Ward staff**

**Ward staff feeling overwhelmed**

**MECHANISM**

**OUTCOME**

**CONTEXT**

**Patient**

**Ward staff**

Burden relief when facing distressing situations [45]

Facilitation of patients’ wishes [46]

**Perceiving that PC specialists have in-depth PC knowledge [24]**

**Ward staff**

**Providing education to ward staff through their expertise [46]**

**Using a no-judgement approach [45]**

**PCT**

**CONTEXT**

**PCT**

**MECHANISM**

**Ward staff**

Being exposed to PC [33]

Having weekly ward rounds with PCT [47]

Believing primary PC is part of their job [30]

Receiving a workshop about PC [48]

Taking up patient education [46]

Taking up staff training and education [46]

**OUTCOME**

**Ward staff**

Enhanced collaboration [33]

**Ward staff cultivating positive perspectives on PCT**

**PCT**

**PCT**

**Patient**

**CONTEXT**

Focus on curative performing culture [49]

Regular interaction [50]

Early consulting [50]

**MECHANISM**

**Ward staff**

**OUTCOME**

Decrease in ICU costs and pharmacy costs [50]

Decrease in medical resource utilization [50]

More patients with DNR codes [50]

Increased hospice referral [50]

**Hospital**

**Ward staff developing expectations regarding PC and the role of PCT**

**Patient**

**MECHANISM**

**CONTEXT**

**PCT**

**Ward staff**

**OUTCOME**

Receiving PC training [35]

Enhanced communication during PCC between all parties [52]

Being easy accessible [51]

Responding quickly [51]

PC physician working bedside with the patient [51]

Being visible/present [33, 39, 51]

**Ward staff**

Teaching opportunities for communication skills [51]

Assistance opportunities with care transitions [51]

More involvement of PCT [35]

Improved understanding of illness [52]

Addressing patient’s problems earlier [39]

**Cultivating feelings of partnership**

**Patient**

**Patient**

**PCT**

**MECHANISM**

**CONTEXT**

Availibility of efficient, shared electronic health record [54]

Taking responsibility in communication [25]

Adhering to principles of patient-centered care [25]

Being embedded within hospital ward [37]

Regular consultation rounds [53]

Having positive perceptions about PC specialists [25]

Willing to be flexible about tasks [25]

**Hospital**

**Ward staff**

**OUTCOME**

**PCT**

**Ward staff**

**PCT**

**Fostering trust**

Facilitation of collaboration [25]

**Ward staff**

**PCT**

**CONTEXT**

**MECHANISM**

Having a lack of PC knowledge [27]

Not feeling recognized by oncologists [28]

Feeling their multidimensional approach is not appreciated [34]

Insufficient human resources within PCT [27]

**Ward staff**

**OUTCOME**

**PCT feeling less motivated to collaborate**

Preventing PCT to encourage ward staff for multidimensional symptom management [34]

**PCT**

**PCT**
