# Supplementary figures and images for: Interprofessional collaboration between hospital-based palliative care teams and hospital ward staff: A realist review
Source: PLoS One. 2025 Dec 19;20(12):e0338132. doi: 10.1371/journal.pone.0338132 (PMC12716714; doi:10.1371/journal.pone.0338132)

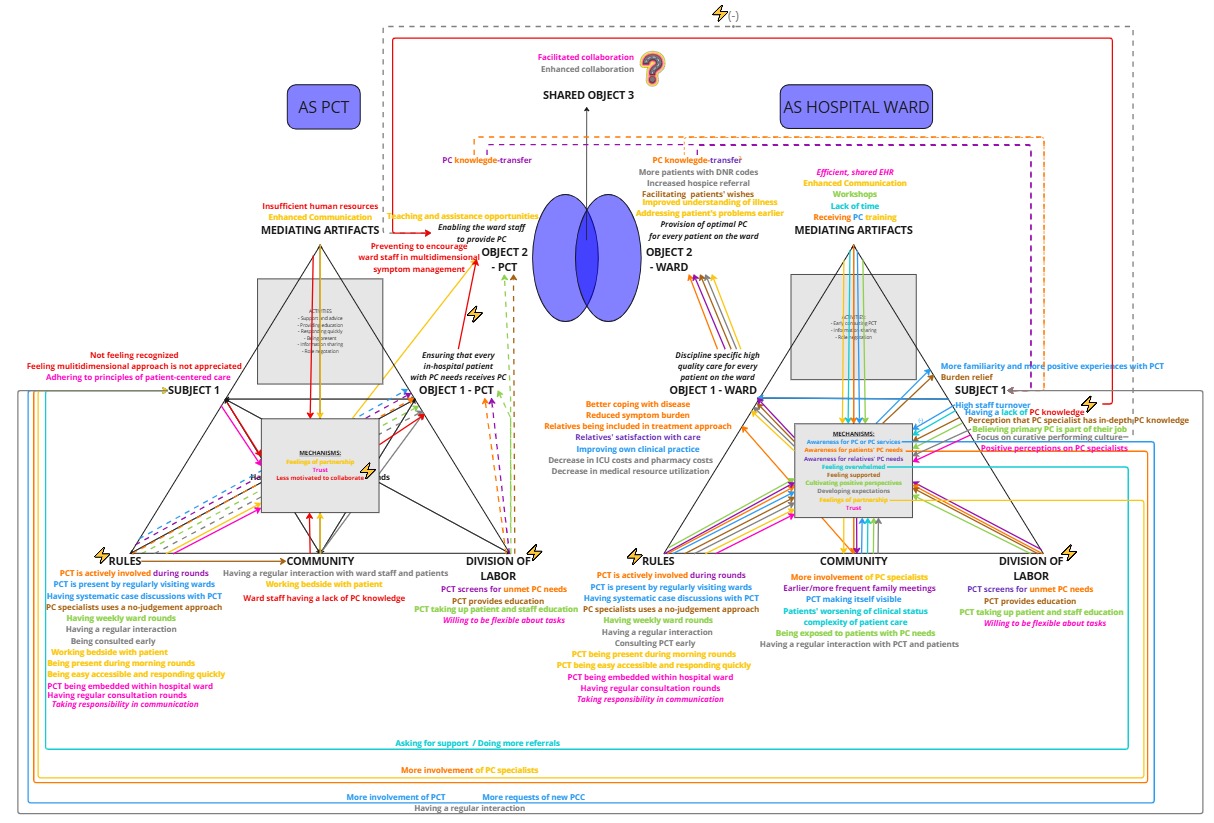

Supplement: S7 Fig — (JPG) [file pone.0338132.s007.jpg]
