# Supplementary material for: Interprofessional collaboration between hospital-based palliative care teams and hospital ward staff: A realist review
Source: PLoS One. 2025 Dec 19;20(12):e0338132. doi: 10.1371/journal.pone.0338132 (PMC12716714; doi:10.1371/journal.pone.0338132)
Supplement: Attachment — Submitted filename: Response to Reviewers.docx [file pone.0338132.s009.docx]

PLOS ONE

Editorial office

***22 October 2025***

**R****ebuttal letter manuscript – “Interprofessional collaboration between hospital-based palliative care teams and hospital ward staff: a realist review”**

Dear Editor-in-Chief, dear Prof. Dr. Chenette,

Dear members of the Editorial Board,

Thank you for the opportunity to revise and resubmit our manuscript entitled *Interprofessional collaboration between hospital-based palliative care teams and hospital ward staff: a realist review* for publication in the Journal of PLOS ONE.

We have carefully considered each point raised by the academic editor and reviewer. On the following pages, we provide a detailed response to each comment and outline the changes we have made to improve our manuscript

We believe that the revisions and clarifications provided in response to the reviewers' comments have strengthened our manuscript. We hope that it now meets the standards for publication in the Journal of PLOS ONE.

We hope you find our revised manuscript suitable for publication and look forward to hearing from you at your earliest convenience.

Yours sincerely,

On behalf of all authors,

Mieke Deschodt

Journal requirements

1. Please ensure that your manuscript meets PLOS ONE's style requirements, including those for file naming**.** The PLOS ONE style templates can be found at:

<https://journals.plos.org/plosone/s/file?id=wjVg/PLOSOne_formatting_sample_main_body.pdf> 
<https://journals.plos.org/plosone/s/file?id=ba62/PLOSOne_formatting_sample_title_authors_affiliations.pdf>

*Thank you for bringing this to our attention. We have adjusted necessary style requirements, including those for file naming and text styles.*

2. If the reviewer comments include a recommendation to cite specific previously published works, please review and evaluate these publications to determine whether they are relevant and should be cited. There is no requirement to cite these works unless the editor has indicated otherwise.

*The reviewers’ comments did not include a recommendation to cite specific previously published
works. Hence, our reference list remained unadjusted.*

Editor Comments:

None.

Reviewers’ comments:

Reviewer #1:

**In general**: This is a well executed realist evaluation of palliative care team interaction with ward staff. Over all this was interesting to read and made some good recommendations. The CMOs are nuanced and were supported extremely well by the figures and diagrams in the Supplementary files. Interesting choice of initial program theory but it works quite well.

*Dear reviewer, thank you for taking the time to review our manuscript. We appreciate your positive feedback and are pleased you found the manuscript satisfactory. Thank you for your considerate questions and suggestions. Please find below some clarifications and adjustments we have carried out based on your feedback.*

**Introduction:** More information about how the palliative care team supports the ward team should be given here. I thought initially they took over full care of the patient. A few extra sentences giving an example of a scenario where the PCT operated as intended would be helpful for readers like me who have not worked in a hospital for many years. Whose call is it to bring in the PC team? What happens? Do they keep monitoring them? How many people in the PC team?

*Thank you very much for your thoughtful comment. We have revised the introduction to include additional detail on the composition of PCTs and added a brief example to clarify their consultative role. While PCTs typically support ward teams rather than assume full responsibility for patient care, their structure and involvement vary considerably across countries and institutions. As such, questions like “Whose call is it?” or “Do they continue monitoring?” are difficult to generalize and were not fully addressed in the introduction. Our primary aim with this paragraph was to emphasize the importance of interprofessional collaboration between PCTs and ward staff, which is central to our study.*

*Page 4, lines 60 – 66:*

“PCTs are multidisciplinary, often including physicians, nurses and psychologist. Rather than taking over primary patient care, PCTs provide consultative support and advise to hospital ward staff regarding palliative care. For example, in a case of a patient with advanced cancer and escalating symptoms, the PCT might support hospital ward staff in advance care planning conversations, adjusting medication prescription, and coordinating family support, while hospital ward staff remains responsible for daily care delivery. This underscores the importance of effective interprofessional collaboration (IPC) to ensure optimal PC delivery [9].”

**Methods**: Can you include some information about the team, elaborating on what expertise and experience was represented?

*We have added a description of the research team to clarify the range of expertise represented,
including clinical experience in palliative care, as well as academic expertise in implementation science, palliative care research, realist review methodology, and interprofessional collaboration.*

*Page 5, lines 88 – 91:*

“The research team included academic experts in implementation science (MD), nursing research (LM), palliative care research (MDR), and realist review methodology and interprofessional collaboration (PP) as well as clinical experts in palliative care (EO, IB).”

**Results:** I liked that you identified the prerequisites for collaboration, rather than trying to shoehorn them into the CMOs. The Supplementary files are very useful.

*Thank you for this positive feedback.*

**Discussion:** Line 501ff “To foster double-loop learning, …. By implementing such changes, interprofessional collaboration (IPC) could be improved, and ward staff would no longer feel burdened by time constraints.” **Not sure this suggestion makes sense. Seems to increase the work of the ward staff rather than lessen it. Can you reconsider this paragraph?** Maybe I have misunderstood and it needs clarifying. **Can you add a comment about other IPC based on this. Perhaps some elements could apply for other specialist teams? If not, can you spell out why PCT interactions are different. Just a sentence or two would do it but I was curious to see what you think**. Figure 2 is very helpful and certainly conveys the complexity of the moving parts.

*Thank you for your thoughtful comment. We agree that the original paragraph may have unintentionally implied an increased workload for ward staff. We’ve revised the section to clarify that perceived time constraints might stem from limited PC confidence rather than actual task volume. The revised paragraph now emphasizes double-loop learning as a systemic solution to reduce this sense of overwhelm. We believe that by integrating structural changes like provision of in-hospital PC training, double-loop learning could be achieved. In our opinion, this approach could empower ward staff in their generalist PC knowledge and skills, making them feel more comfortable in providing PC and feeling less overwhelmed or constrained by time, without adding to their burden.*

*Page 22, lines 508 – 516:*

“Friction also arises when staff is confronted with a patient’s worsening clinical status but are constrained by lack of time. However, this perceived time pressure may be rooted in limited PC knowledge, leaving ward staff to feel overwhelmed, causing them to ask support from their PCT—a response characteristic of single-loop learning. To foster double-loop learning, hospitals should address these underlying causes of experiencing lack of time due to limited PC knowledge by investing in systemic solutions, for example a yearly PC training for ward staff. Such initiatives can gradually shift norms within the system and increase comfort with PC tasks among ward staff, reducing the sense of being overwhelmed even if the workload remains unchanged. While this approach may initially require additional effort, it can enhance efficiency and interprofessional collaboration (IPC) in the long term.”

**A few typos or minor grammar issues:**

- Line 89: “… as a considerate number of papers” should be “considerable”
- Line 114: “…for a mechanism to get triggered” should be “for a mechanism to be triggered.”
- Line 175: “(physicians, nurses, social 9 176 workers…).” Better to say (e.g., physicians, nurses, social workers)
- Line 255: “non-judgement approach” should be non-judgemental

*Thank you for bringing these to our attention. We have adjusted the typos and minor grammar issues based on your feedback. In addition, I also adjusted some typos at lines 301 and 302.*
